# Mechanistic Insights into G Protein-Biased κ‑Opioid Receptor Signaling Using Dual-Charged Naltrexamine Amides

**DOI:** 10.1021/acs.jmedchem.5c02135

**Published:** 2026-02-05

**Authors:** Niklas Piet Doering, Kristina Puls, Marta Diceglie, Anja Meraner, Axel Hentsch, Siriwat Hongnak, Armin Wurzer, Helmut Schmidhammer, Mariana Spetea, Marc Nazare, Gerhard Wolber

**Affiliations:** † Department of Biology, Chemistry and Pharmacy, 9166 Institute of Pharmacy, Molecular Design Group, Königin-Luise-Str. 2 + 4, Berlin 14195, Germany; ‡ Medicinal Chemistry, 28417 Leibniz-Forschungsinstitut für Molekulare Pharmakologie (FMP), Campus Berlin Buch, Robert-Roessle-Str. 10, Berlin 13125, Germany; § Department of Pharmaceutical Chemistry, Institute of Pharmacy and Center for Molecular Biosciences Innsbruck (CMBI), 27255 University of Innsbruck, Innrain 80-82, Innsbruck 6020, Austria

## Abstract

Opioids remain a cornerstone of pain management, but currently used therapeutics are associated with serious side effects. While κ-opioid receptor (KOR) agonists offer an alternative to classical μ-opioid receptor (MOR) agonists, their clinical potential is limited by severe adverse effects. G protein-biased KOR agonists are a promising strategy for developing safer analgesics. In this study, we used virtual screening to develop novel dual-charged naltrexamine amide derivatives as tool compounds for investigating biased agonism at the KOR. All of the predicted ligands demonstrate low-nanomolar activity and G protein bias at both the KOR and MOR. Molecular dynamics simulations revealed a key allosteric communication involving TM4, TM5, and ICL2. These compounds achieve their effects through interactions with residues E209^ECL2^, D223^5.35^, E297^6.58^, and K227^5.39^. These findings provide insight into the structural mechanisms of KOR signaling bias and inform the rational design of improved KOR therapeutics.

## Graphical abstract

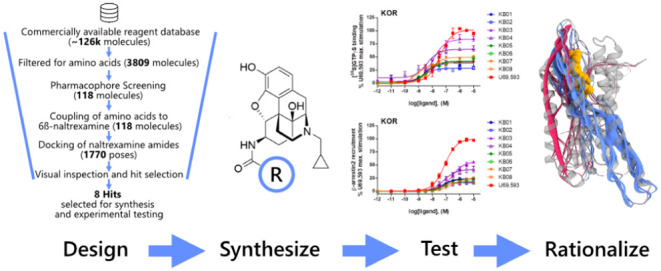

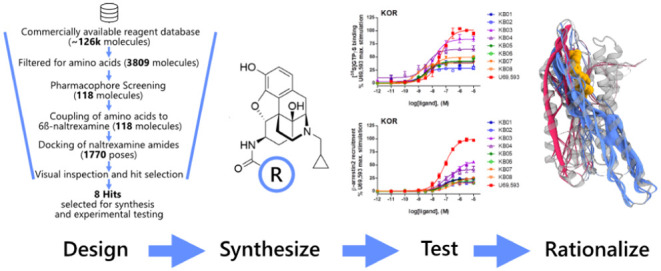

## Introduction

Opioids have long been central to pain management,^1^ yet widely used μ-opioid receptor (MOR) agonists such as morphine and fentanyl are associated with serious adverse effects, including addiction and respiratory depression.^2,3^ This has intensified the search for safer analgesics, with the κ-opioid receptor (KOR) emerging as a compelling target due to its potential to provide pain relief without MOR-associated liabilities.^4–7^ However, clinical application of KOR agonists is limited by their own side effects, including dysphoria, sedation, psychotomimesis, and anxiety in humans.^8–10^ A promising strategy to overcome these challenges involves the development of functionally selective (biased) KOR ligands that preferentially activate analgesic pathways while sparing those responsible for undesirable effects.^7,11–13^

Despite growing interest in biased agonism, the structural mechanisms underlying G protein bias at the KOR remain elusive. Uprety and colleagues^14^ proposed that ligand occupancy of distinct receptor regions may differentially regulate signaling pathways: engagement of the upper transmembrane helix (TM) 5/extracellular loop (ECL) 2 region favors G protein signaling, whereas occupancy of the lower TM2/TM3 interface promotes β-arrestin recruitment.^14^ Their hypothesis is based on a combination of *in silico* modeling and *in vitro* pharmacological assays using the amidoepoxymorphinan ligand MP1104 (Figure 1) and derivatives. The study revealed a correlation between the conformation of the morphinan C-ring and the spatial orientation of the ligand’s substituent either toward TM5/ECL2 or TM2/TM3.^14^ Ligands predicted to adopt a chair conformation oriented toward TM5/ECL2 exhibited G protein bias, whereas those favoring a boat conformation, such as MP1104, extended toward TM2/TM3 and elicited robust arrestin signaling.^14^ Notably, this structure–function relationship was proposed to be conserved across both the KOR and MOR systems.^14^

**Figure 1:**
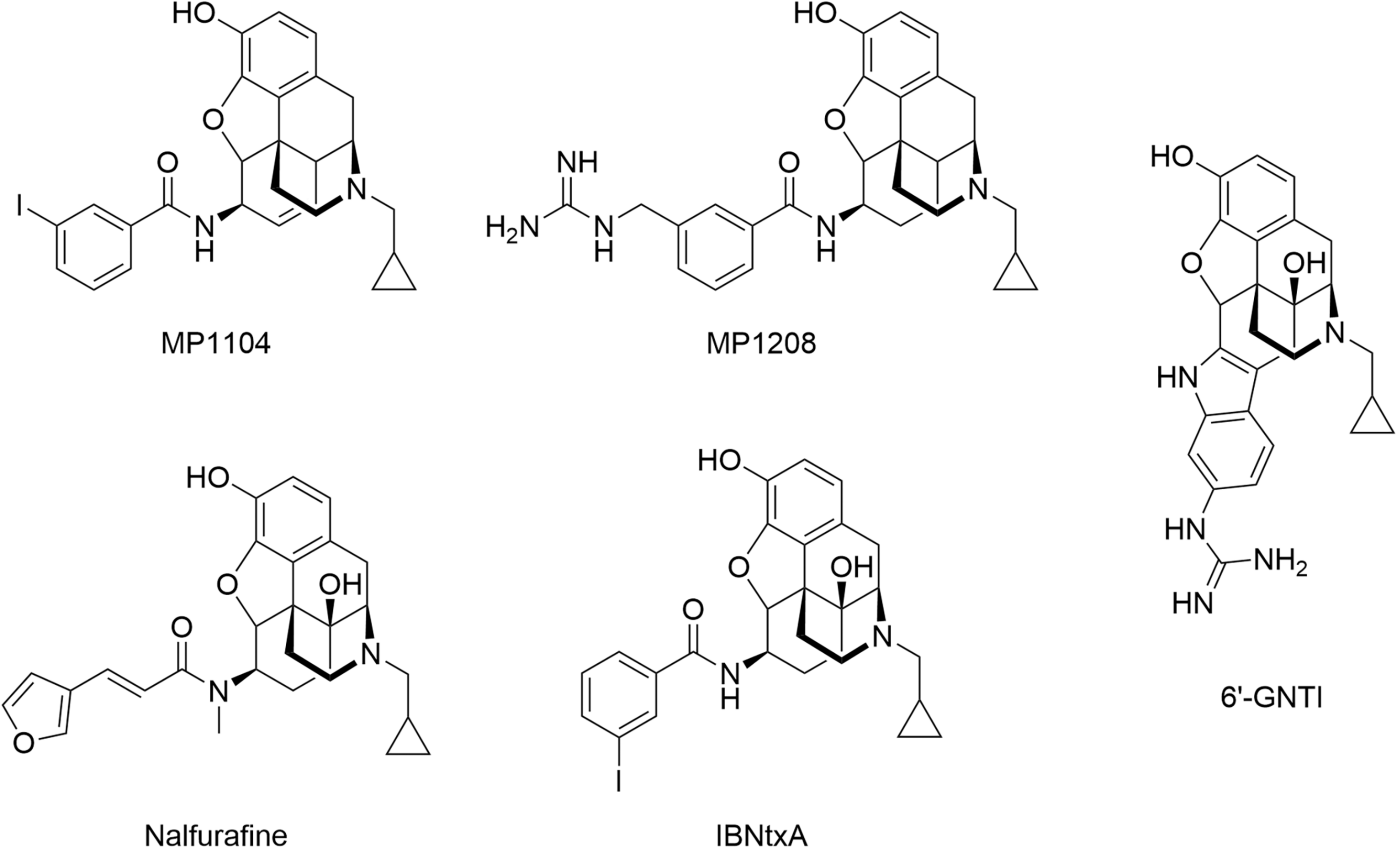
Representative examples of morphinan KOR and MOR ligands evaluated for signaling bias. The unbiased ligand MP1104 was derived from the KOR crystal structure (PDB ID: 6B73).^15^ Biased ligands include MP1208 and 6‘-GNTI, both are proposed to exhibit the TM5/ECL2 interaction hypothesis by Uprety et al.^14^ Additional ligands include the G protein-biased agonists nalfurafine^16^ and IBNtxA^15^ characterized as MOR-biased and KOR-unbiased, respectively.

El Daibani and colleagues^16^ recently reported the crystal structure of nalfurafine bound to the KOR (PDB ID: 7YIT^16^), a ligand shown to exhibit G protein bias.^16,17^ To elucidate the molecular basis of its bias, the authors conducted extensive molecular dynamics (MD) simulations of three KOR ligands: the G protein-biased agonist nalfurafine, the balanced agonist U50,488, and the arrestin-biased agonist WMS-X600. Their analysis identified three key structural features associated with G protein bias at the KOR. First, disruption of the intramolecular salt bridge between K227^5.39^ and E297^6.58^ was found to correlate with G protein signaling. The loss of this interaction increased the distance between the extracellular regions of TM5 and TM6, a change that may propagate conformational shifts to the intracellular interface.^16^ Notably, E297^6.58^ has been previously implicated in ligand-specific activation profiles, including in studies of KOR ligands 5′-GNTI and 6’-GNTI (Figure 1), where interactions with this residue are thought to modulate TM6 rotation and receptor signaling.^18–20^

Second, ligand interactions with Q115^2.60^ appear to promote G protein bias. In MD simulations, nalfurafine engaged Q115^2.60^ and stabilized its orientation toward TM3, whereas balanced and arrestin-biased ligands did not interact with this residue. Instead, Q115^2.60^ rotated toward TM1, facilitating a counterclockwise rotation of the extracellular portion of TM7. Analogous behavior has been reported for the MOR, where the G protein-biased mitragynine pseudoindoxyl (MP) interacts with Q124^2.60^ to disrupt a hydrogen bond with Y326^7.43^, a bond thought to promote arrestin recruitment.^21^

Third, the emergence of an “occluded” receptor conformation was uniquely associated with G protein bias. In MD simulations, only the nalfurafine–KOR complex adopted this conformation, characterized by a clockwise rotation and inward displacement of the intracellular end of TM7 toward TM2.^16^ Additionally, the residue W287^6.48^, part of the conserved CWxP motif critical for GPCR activation,^22–24^ was implicated in bias signaling. Mutation of W287^6.48^ to alanine impaired arrestin recruitment more than G protein activation, suggesting a preferential role in arrestin pathway engagement. Notably, the arrestin-biased ligand WMS-X600 displaced the W287^6.48^ side chain further downward compared to balanced or G protein-biased ligands in simulation.^16^

An additional residue implicated in biased signaling at the KOR is Y312^7.35^, located at the upper portion of TM7.^15^ The morphinan derivative IBNtxA (Figure 1) functions as an agonist at both the KOR and MOR, yet exhibits G protein bias exclusively at the MOR.^15^ Mutation of Y312^7.35^ to tryptophan, mirroring the corresponding MOR residue, resulted in reduced arrestin signaling at the KOR, suggesting that Y312^7.35^ plays a role in functional selectivity.^15^ One proposed explanation for IBNtxA’s selective G protein bias at the MOR lies in differences in the physicochemical properties of the TM2/TM3 region between the two receptors. Specifically, this region is more hydrophobic in the KOR than in the MOR.^14^ As a result, IBNtxA’s hydrophobic 3-iodobenzoyl moiety may preferentially orient toward the TM2/TM3 region in the KOR, correlating with enhanced arrestin signaling. In contrast, the less hydrophobic MOR environment may favor alternate orientations associated with G protein bias. These findings suggest that receptor-specific differences in the TM2/TM3 microenvironment could serve as critical determinants of functional selectivity.^14^

Building on the hypothesis by Uprety et al.^14^ which implicates the TM5/ECL2 region in G protein-biased signaling at the KOR, this study aims to rationalize the development of biased KOR agonists. Using *in silico* methods, we designed novel morphinan analogs targeting this region, which were subsequently synthesized and pharmacologically evaluated for efficacy and signaling bias. To further elucidate the underlying mechanisms, we performed structure–activity relationship (SAR) analysis *in silico*, aiming to establish molecular mechanisms for biased agonism at the KOR and advance ligand design beyond serendipitous discovery.

## Results

### *In Silico* Design of Novel Dual-Charged Naltrexamine Amides for G Protein-Biased KOR Agonism

MP1208 (Figure 1), one of the lead compounds identified by Uprety and coworkers^14^ is a morphinan ligand with a 3-guanidinomethylbenzamide moiety proposed to interact with D223^5.35^ and E209^*ECL2*^ of KOR. MP1208 shows good KOR and MOR affinity (*K*_*i*_ nM: mKOR: 0.28; mMOR: 0.34), potency (*EC*_50_ nM: mKOR: 1.36; MOR: 1.13) and G protein bias (bias factor­(cAMP/Tango): hKOR: 22) together with a beneficial side effect profile.^14^ MP1208 showed neither reward nor aversive behavior in mice.^14^ In-house performed docking experiments of MP1208 confirmed ionic interactions to D223^5.35^ and E209^*ECL2*^ of the KOR. MD simulations of the MP1208-KOR complex revealed that additional ionic interactions to E297^6.58^ exist in 30.68% of simulation frames. Interestingly, the disruption of an intramolecular salt bridge between E297^6.58^ and K227^5.39^ is thought to promote G protein bias.^16^ Thus, protein–ligand interactions with E297^6.58^ likely promote G protein bias. Furthermore, previous publications postulated that the G protein-biased KOR agonist 6’-GNTI directly interacts with E297^6.58^ via its guanidine moiety.^19,20^ In-house docking experiments of 6’-GNTI confirmed this hypothesis with 6’-GNTI’s guanidine moiety interacting with E209^*ECL2*^ and E297^6.58^. From the evidence provided, we hypothesized interactions toward the negative triad of D223^5.35^, E209^*ECL2*^, and E297^6.58^ in the upper TM5/ECL2/TM6 region could be beneficial for G protein bias and therefore be interesting for further exploration.

We aimed to assess the role of the negative triad for G protein bias at the KOR by insertion of a basic moiety that stabilizes the ligand in the TM5/ECL2/TM6 region. For that, we derivatized the already known morphinan ligand 6β-naltrexamine at its 6-position to ensure that differences in the pharmacological evaluation of this compound series originates from the differences in the substitution pattern. 6β-Naltrexamine is a valuable starting point for derivatization for a number of reasons: (i) 6β-Naltrexamine neither introduces nor prevent G protein bias. It is part of the known G protein biased KOR ligand nalfurafine^16^ but also of IBNtxA that does not show G protein bias at the KOR.^14^ (ii) 6β-Naltrexamine is synthesizable in larger amount from the readily available compound naltrexone.

For the rational design of 6β-naltrexamine derivatives we performed a 3D pharmacophore-based virtual screening for basic fragments fitting into the TM5/ECL2/TM6 region. These fragments were subsequently linked to the 6β-naltrexamine scaffold to generate our dual charged naltrexamine amides used as tool compounds. In the following the generation and selection process of the dual charged naltrexamine amides shall be described (Figure 2A). A 3D pharmacophore for the virtual screening of suitable 6β-naltrexamine substituents was generated using LigandScout 4.4 ^25^ and derived by modification of the 3D pharmacophore of MP1208’s 3-guanidinomethylbenzamide docked to the KOR. Particularly, an additional exclusion volume coat was added for size restriction during the virtual screening, the feature size tolerance of the positive ionizable (PI) feature was increased to 2.0, the hydrogen bond donator features of MP1208’s guanidine group were deleted to allow for the replacement of the guanidine moiety by other basic moieties, and an additional negative ionizable feature at the position of MP1208’s amine linker was added (Figure 2B). The negative ionizable feature was added to filter for carboxylic acid moieties that can be reacted with the amine moiety at the 6-position of 6β-naltrexamine to synthesize the final KOR ligands.

**Figure 2:**
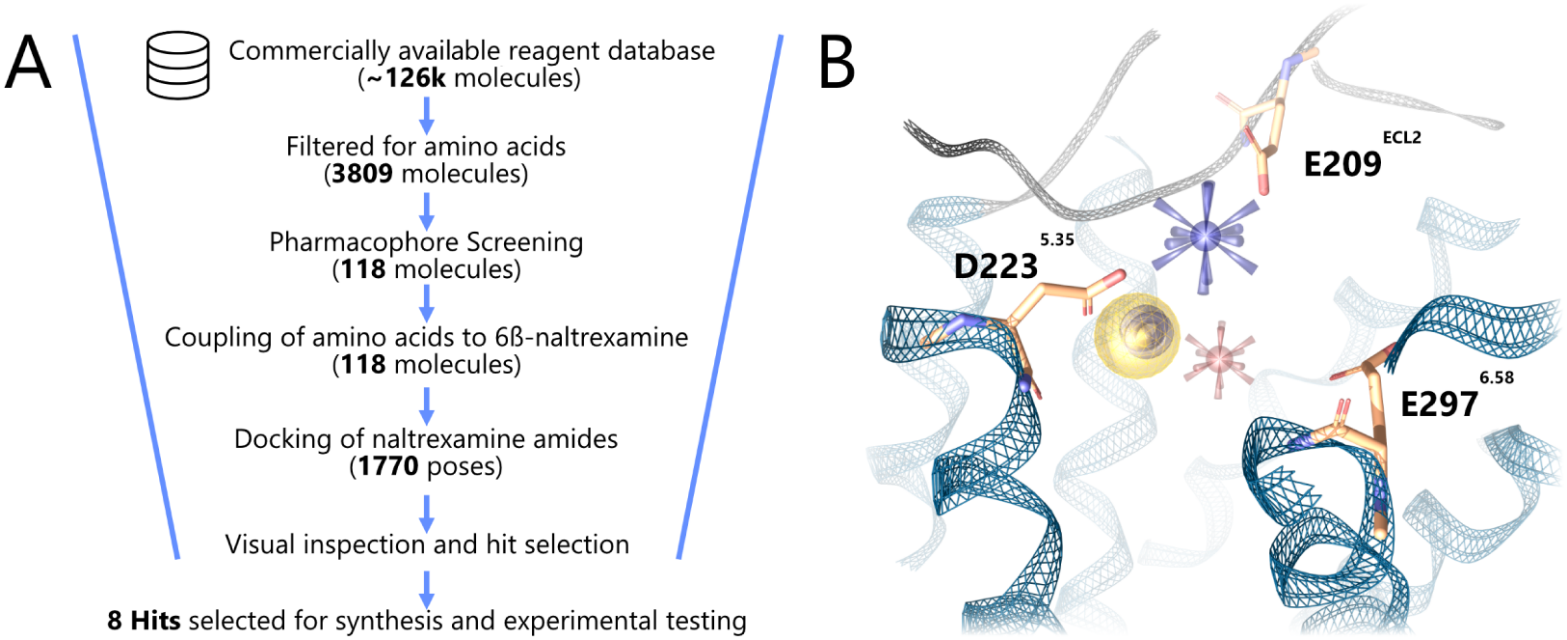
(A) Schematic workflow of the virtual screening campaign for biased dual charged naltrexamine amides, including the number of compounds remaining after each step. (B) Representation of the screening pharmacophore within the context of the KOR binding pocket (PDB ID: 6B73 ^15^). The negative triad is shown in orange. Positive ionizable: blue star; negative ionizable: red star; aromatic interaction: blue circle; hydrophobic contact: yellow sphere.

Prior the virtual screening to identify suitable naltrexamine substituents a database consisting of unnatural amino acids needed to be generated to ensure the presence of a basic substituent after amino acid-coupling to 6β-naltrexamine. The library chosen for amino acid filtering was the’Enamine REAL reagents’ from 2021 ^26^ (∼126k compounds) with the filter criteria of the presence of at least one basic moiety and one carboxylic acid resulting in 3,809 retrieved amino acids. These amino acids represent the amino acid database used in the following paragraph. The amino acid filtering process was conducted in KNIME v4.5.2 ^27^ using RDKit.^28^

The virtual screening for those amino acids within the amino acid library that fulfill the previously described MP1208-derived 3D pharmacophore and therefore represent suitable naltrexamine substituents was conducted in LigandScout 4.4.^25^ A total number of 118 hit molecules fulfilled the geometrical and electrostatical screening criteria and therefore were selected for in silico linkage to the 6-position of 6β-naltrexamine. The linkage was conducted via the formation of an amid bond between the primary amine of 6β-naltrexamine and the carboxylic acid of the hit. The newly substituted naltrexamine amides carrying two positive ionizables (one in the naltrexamine core, one in the linked substituent) were docked into the active-state KOR crystal structure (PDB ID: 6B73 ^15^) to identify the derivatives with the best interaction pattern to the TM5/ECL2/TM6 region and its negative triad (D223^5.35^, E209^*ECL2*^, E297^6.58^). The final ligand series chosen for synthesis was selected according multiple parameters in a visual inspection. First, the 3D pharmacophore similarity toward docked MP1208, the shape overlay to MP1208, and the number of interaction features were calculated in LigandScout. Ligands were deemed meaningful only when they met all three criteria in a favorable manner. Second, meaningful ligands needed to form interactions to the negative triad (D223^5.35^, E209^*ECL2*^, and E297^6.58^). On the contrary, ligands with interactions toward Y312^7.35^ were discarded as this interaction likely promotes β-arrestin2 recruitment.^15^ Finally, plausible ligand geometries, rigidity, and ligand diversity were considered. Through this selection process, eight molecules qualified for organic synthesis and subsequent pharmacological evaluation.

### Synthesis of Novel Dual-Charged Naltrexamine Amides

Following the *in silico* design of the tool compound series (**KB** series), the identified hits were synthesized by adapting the reported procedure from Ghirmani et al.^29^ (Scheme 1). The key intermediate 6β-naltrexamine **3** for all final amide functionalized compounds **KB01-KB08** was obtained from commercially available naltrexone **1** using a short oximation-reduction sequence.^29^ First, **1** was converted into the corresponding oxime **2** by treatment with Naltrexone, NH_2_OH·HCl and NaOAc in ethanol followed by subsequent reduction to the corresponding amine **3** using BH_3_·THF, yielding pure 6ß-naltrexamine diastereomer **3** in 57% yield.

**Scheme 1:**
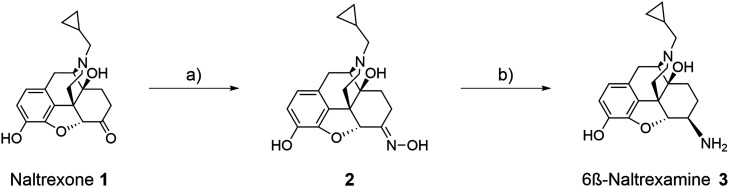
Reaction Scheme for the Synthesis of 6*β*-Naltrexamine **3** [Fn sch1-fn1]

i Reagents and conditions: (a) Naltrexone **1** (1 equiv), NH_2_OH·HCl (1.5 equiv), NaOAc (2.5 equiv), EtOH, 78 °C, 2.5 h. (b) BH_3_·THF (18 equiv), THF, 66 °C, 48 h.

The 6β-naltrexamine building block **3** was then coupled with the respective eight carboxylic acids using standard amide coupling conditions (Scheme 2). The respective carboxylic acid derivative was activated using EDCI and HOAt in the presence of 4 Å molecular sieves and TEA, followed by the addition of the 6β-naltrexamine **3**. Here, we observed concomitant esterification of the 3-hydroxy position of 6β-naltrexamine **3**, resulting in the intermediate **4** as a crude. Intermediate **4** was then treated with K_2_CO_3_ in methanol to selectively hydrolyze the ester group to obtain the final compounds **KB01-KB08** (12–57% yield).

**Scheme 2:**
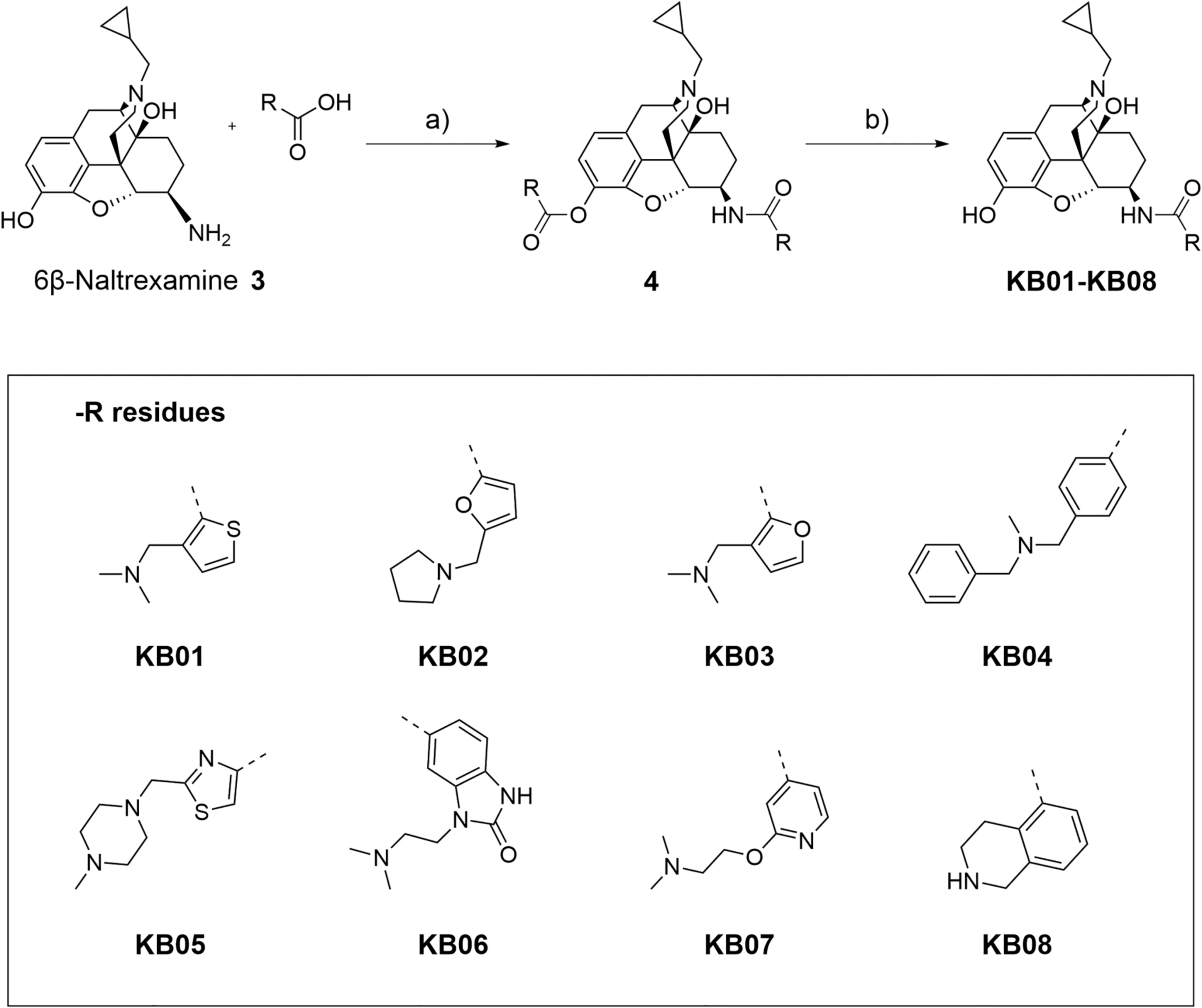
Reaction Scheme for the Synthesis of Final Compounds **KB01-KB08**, with the Eight Different Substituents[Fn sch2-fn2]

i (a) 6*β*-Naltrexamine **3** (1 equiv), carboxylic acid (2 equiv) EDCI (3 equiv), HOAt (3 equiv), TEA (4 equiv), DMF, RT, 5 h. (b) K_2_CO_3_ (3 equiv), MeOH, RT, 16 h.

### *In Vitro* Pharmacological Evaluation of Novel Dual-Charged Naltrexamine Amides

The synthesized morphinan compounds of the **KB** series (**KB01-KB08**) are 6β-amidoepoximorphinans with a hydroxyl group in position 14, a phenolic hydroxyl group in position 3 and a cyclopropylmethyl group at the N17 position (Scheme 2). The compounds **KB01-KB08** were structurally modified by targeting different substitutions in position 6.

The *in vitro* pharmacological evaluation of the eight morphinans **KB01-KB08** was initially performed using a radioligand-based competitive binding assays with membranes from Chinese hamster ovary (CHO) cells stably expressing one of the recombinant human opioid receptor, KOR, MOR, or δ-opioid receptor (DOR).^30^ Their binding properties were compared to the profile of the parent epoxymorphinan 6β-naltrexamine, and other known epoxymorphinan ligands, including morphine, nalfurafine^31^ and naltrindole^32^ with selectivity for the MOR, KOR, or DOR, respectively. Based on the measured binding affinities (*K*_*i*_), all compounds bound with high affinity to the human KOR, ranging from 0.44 nM to 1.73 nM (Table 1 and Table S1). They also displayed increased or comparable binding at the KOR to 6ß-naltrexamine, U69,593, and reduced affinity than nalfurafine. Notably, the compounds exhibited a marked increase in binding affinity at the human MOR than 6ß-naltrexamine and morphine, with potency in the picomolar to low nanomolar range (*K*_*i*_ values from 0.071 to 1.49 nM). In contrast, binding at the human DOR was substantially reduced compared to MOR and KOR (Table 1 and Table S1), consistent with the distinct electrostatic profile of the targeted TM5/ECL2/TM6 binding region. While this region contains multiple negatively charged residues in the KOR and MOR, it is comparatively neutral in the DOR and thus is missing the negative triad. Compared to 6β-naltrexamine, all compounds in the **KB** series except **KB03** displayed increased affinities at the DOR, but have considerably reduced binding than naltrindole. Based on these findings, most ligands in the **KB** series exhibited a dual KOR/MOR binding profile. We should also mention the *K*_*i*_ value in the picomolar range of **KB01** at the human MOR (*K*_*i*_ = 0.071 nM), revealing a ligand with MOR selectivity (16-fold vs KOR and 356-fold vs DOR). A comparable profile was observed for the **KB04**, with a *K*_*i*_ value of 0.10 nM at the MOR, and being 17- and 346-fold more selective for the MOR vs KOR and vs DOR, respectively (Table 1).

**Table 1:** Binding Affinities of **KB** Series at the Human Opioid Receptors

	Binding *K*_*i*_ (nM)^a^		
Ligand	KOR	MOR	DOR
**KB01**	1.13 ± 0.56	0.071 ± 0.009	25.3 ± 8.7
**KB02**	1.73 ± 0.69	0.56 ± 0.01	83.5 ± 14.4
**KB03**	1.14 ± 0.34	1.49 ± 0.15	282 ± 90.3
**KB04**	1.73 ± 0.48	0.10 ± 0.05	34.6 ± 11.3
**KB05**	0.59 ± 0.05	0.39 ± 0.06	104 ± 38.6
**KB06**	1.23 ± 0.40	0.17 ± 0.02	139 ± 80.9
**KB07**	0.92 ± 0.09	0.49 ± 0.05	142 ± 79.5
**KB08**	0.44 ± 0.16	0.20 ± 0.03	147 ± 60.1
**6β-Naltrexamine**	1.11 ± 0.32	1.20 ± 0.20	214 ± 40
**Nalfurafine**	0.043 ± 0.016	-	-
**Morphine**	-	3.61 ± 0.28	-
**Naltrindole**	-	-	0.30 ± 0.06
**U69,593**	1.71 ± 0.42	-	-
**DAMGO**	-	1.46 ± 0.37	-
**DPDPE**	-	-	3.08 ± 0.14

a Determined in radioligand competitive binding assays with CHO cell membranes stably expressing the one of the recombinant human opioid receptors. - Denotes not tested. Values are means ± SEM of at least three independent experiments.

Next, the *in vitro* functional activities on G protein activation and β-arrestin2 recruitment of the eight morphinan compounds of the **KB** series (**KB01-KB08**) at the human KOR and MOR was evaluated. Each analogue was screened for its ability to induce G protein activation at the KOR and MOR using the [^35^ *S*]­GTPγS functional assay with membranes from CHO cells expressing the human KOR or MOR, performed as described.^30^ The β-arrestin2 recruitment profiles to the human KOR and MOR were determined using a cell-based assay, the PathHunter β-arrestin2 recruitment assay with U2OS cells coexpressing the human KOR or MOR, and the enzyme acceptor tagged β-arrestin2 fusion protein.^30,33^ In both functional assays, test compounds were examined in parallel with U69,593 or DAMGO, which served as reference KOR and MOR agonists, respectively. Nalfurafine^31^ HS665 ^34^ and HS666 ^34^ were included as positive controls in the functional assays at the KOR, whereas the PZM21 ^35^ and TRV130 ^36^ were used as positive controls in the functional assays at the MOR. Agonist potency (*ED*_50_) and efficacy (*E*_*max*_) were calculated for concentration–response curves (Figure S1), and values are listed in Tables 2 and 3.

**Table 2:** Functional Activity of **KB** Series on G Protein Activation and *β*-arrestin2 Recruitment at the Human KOR

	G Protein^a^		β-arrestin2^b^		
Ligand	*EC*_50_ (nM)	*E*_*max*_ (%)	*EC*_50_ (nM)	*E*_*max*_ (%)	Bias factor^c^
**KB01**	3.85 ± 0.22	38.6 ± 6.0	61.4 ± 20.8	24.6 ± 1.8	9.8
**KB02**	4.10 ± 1.66	29.8 ± 1.9	46.2 ± 3.1	17.3 ± 3.7	7.6
**KB03**	10.2 ± 1.5	82.7 ± 6.3	297 ± 60	57.3 ± 2.7	17
**KB04**	4.05 ± 1.08	64.5 ± 3.3	79.3 ± 21.1	41.4 ± 5.4	12
**KB05**	3.26 ± 0.75	41.1 ± 1.1	74.2 ± 16.4	23.9 ± 2.8	14
**KB06**	5.78 ± 0.70	44.7 ± 2.9	121 ± 31	21.2 ± 3.7	17
**KB07**	6.13 ± 0.93	49.7 ± 6.5	404 ± 90	26.2 ± 3.2	49
**KB08**	1.14 ± 0.61	39.2 ± 2.4	45.8 ± 17.0	16.0 ± 3.3	39
**Nalfurafine**	0.045 ± 0.007	96.4 ± 2.4	3.59 ± 0.67	85.8 ± 0.5	35
**HS665**	4.34 ± 1.0	87.4 ± 4.2	572 ± 111	59.3 ± 1.8	76
**HS666**	29.7 ± 7.2	48.4 ± 1.9	570 ± 107	28.7 ± 2.4	12
**U69,593**	32.7 ± 9.0	100	83.2 ± 12.7	100	1

a Determined in the [^35^ *S*]­GTPγS binding assay.

b Determined in the PathHunter ß-arrestin2 recruitment assay. Within each assay, potency (*EC*_50_) and efficacy (*E*_*max*_) values were calculated by nonlinear regression.

c Bias factors were determined by the operational model, relative to U69,593.^40^ Values represent means ± SEM of at least three independent experiments.

**Table 3:** Functional Activity of **KB** Series on G Protein Activation and *β*-arrestin2 Recruitment at the Human MOR

	G Protein^a^		β-arrestin2^b^	
Ligand	*EC*_50_ (nM)	*E*_*max*_ (%)	*EC*_50_ (nM)	*E*_*max*_ (%)
**KB01**	0.18 ± 0.05	48.1 ± 9.6	-	-
**KB02**	0.94 ± 0.24	28.5 ± 7.8	-	-
**KB03**	2.87 ± 0.98	48.7 ± 5.8	-	-
**KB04**	0.076 ± 0.023	32.9 ± 5.0	-	-
**KB05**	3.19 ± 0.71	44.0 ± 8.2	-	-
**KB06**	1.98 ± 0.63	19.7 ± 3.7	-	-
**KB07**	0.19 ± 0.04	37.4 ± 3.9	-	-
**KB08**	1.14 ± 0.54	30.9 ± 2.1	-	-
**PZM21**	10.4 ± 3.1	74.5 ± 2.6	-	-
**TRV130**	8.14 ± 1.64	88.5 ± 5.0	-	-
**DAMGO**	20.7 ± 9.5	100	350 ± 42	100

a Determined in the [^35^ *S*]­GTPγS binding assay.

b Determined in the PathHunter ß-arrestin2 recruitment assay. Percentage stimulation (*E*_*max*_) is presented relative to reference MOR agonist, DAMGO. - Denotes no measurable activity up to 10 μM values represent means ± SEM of at least three independent experiments.

In contrast to U69,593, all compounds in the **KB** series were much more potent (3- to 29-fold) in inducing KOR-mediate G protein activation as assessed in the [^35^ *S*]­GTPγS functional assay (Table 2)). The most potent KOR ligand was **KB08** with an *EC*_50_ value of 1.14 nM, which also presented the highest KOR binding affinity (*K*_*i*_ = 0.44 nM) in competition binding studies (Table 1 and Table S1). Additionally, all ligands, except of **KB03**, were partial agonists at the human KOR, based on the reduced efficacies (29.8–64.5% of U69,593 stimulation) in G protein activation. Only analogue **KB03** displayed high efficacy (82.7% of U69,593 stimulation) profiling it as full agonist at the human KOR (Table 2).

To investigate the capability to promote KOR-mediated β-arrestin2 signaling, **KB01-KB08** were profiled for their potency and efficacy in a β-arrestin2 recruitment assay. All eight morphinan analogues produced concentration-dependent effect in ß-arrestin2 recruitment (Figure S1) with different levels of potencies (*EC*_50_ vales from 45.8 nM to 404 nM) (Table 2). Two compounds, **KB03** and **KB07**, displayed lower potencies (4- and 5-fold) in inducing ß-arrestin2 recruitment than U69,593 (*EC*_50_ = 83.2 nM). The rest of the analogues showed higher or comparable potencies in promoting ß-arrestin2 recruitment, when compared to U69,593. The highest potency was determined for **KB08**, with an *EC*_50_ value of 45.8 nM. In contrast to U69,593, which was very efficaciously recruited β-arrestin2 to the KOR upon ligand binding, all compounds were much less effective based on the calculated *E*_*max*_ values ranging from 16% (for **KB08**) to 57.3% (for **KB03**) of U69,593 stimulation, thus confirming them as partial agonists.

To examine whether the compounds in the **KB** series (**KB01-KB08**) display bias at the human KOR toward the activation of G protein- over β-arrestin2-mediated signaling, we compared their functional activity across the two functional assays that measure G protein coupling and β-arrestin2 recruitment. The G protein-biased KOR agonists nalfurafine,^17,37^ HS665,^38,39^ and HS666 ^38,39^ were used as positive controls. Based on the calculated biased factors^40^ all **KB** compounds are G protein-biased agonists at the KOR with values ranging between 7.6 and 49 (Table 2). The highest bias factor was calculated for **KB07**, followed by the analogue **KB08**. The potency and bias effects for the compound series in Table 2 are made even more apparent, when comparing full concentration–response curves, as shown in Figure S2. A rightward shift in the concentration–response curves of **KB01-KB08** in the PathHunter ß-arrestin2 recruitment assay compared to the [^35^ *S*]­GTPγS binding assay is observed, paralleled by a substantial reduction in efficacy.

Based on the good binding to the human MOR measured for the compounds in the **KB** series (**KB01-KB08**) in radioligand competitive binding assays, we further aimed in evaluating their functional activity at this receptor in the [^35^ *S*]­GTPγS binding and β-arrestin2 recruitment assays. The G protein-biased MOR agonists PZM21 ^35^ and TRV130 ^36,41^ were used as positive controls. As shown in Table 3, they displayed very high potencies in inducing G protein activation after ligand binding to the MOR, with *EC*_50_ values ranging between 0.076 nM and 3.19 nM, being up to 7-fold more potent than DAMGO (*EC*_50_ of 20.7 nM). The most potent compound was **KB04**, with a 270-fold increased potency that DAMGO in G protein activation at the MOR, which also exhibited a very high MOR binding affinity (*K*_*i*_ = 0.10 nM) in competition binding studies (Table 1 and Table S1). Regarding efficacy, all compounds were partial agonists at the MOR based on the calculated *E*_*max*_ values (range 19.7–48.7% of DAMGO stimulation). At the human MOR, none of the compounds in the **KB** series (**KB01-KB08**) induced β-arrestin2 recruitment upon receptor activation, whereas the reference MOR agonist, DAMGO, effectively recruited β-arrestin2 with an *EC*_50_ value of 350 nM (Table 2). Similar to PZM21 and TRV130, all KB compounds exhibit efficacy at the MOR for G protein activation in the [^35^ *S*]­GTPγS binding assay (Figure S3). Therefore, there is evident bias in favor of G protein signaling. However, the β-arrestin2 recruitment signal was too low within the tested concentration range (up to 10 μM) to allow the bias factors to be formally determined.

### *In Silico* Insights into Molecular Mechanisms of Bias at the KOR

Following the *in vitro* pharmacological evaluation, we conducted further *in silico* investigations to explore the underlying mechanisms of bias and partial agonism of our compounds. Specifically, we examined **KB05**, the compound with the highest binding affinity; **KB07**, which exhibited the greatest G protein bias; and **KB03**, which showed the least partial agonism within the context of the KOR. For comparison, we also included the unbiased ligand MP1104 ^14^ and the known biased ligand nalfurafine^16^ both of which have previously been structurally characterized in complex with the KOR.^15,16^ Furthermore, we investigated **KB07** compared to the unbiased full agonist morphine in the context of the MOR.

Interaction analysis of the binding site during MD simulations was performed using Dynophores^42–46^ a toolkit that allows the identification of protein–ligand interactions over the course of an MD simulation. The analysis revealed that the morphinan core remained relatively stable across all simulations of the **KB** series compounds. However, the attached moieties exhibited distinct interaction patterns throughout the simulations. All three compounds, **KB03**, **KB05**, and **KB07**, tended to deviate from their initial binding modes, which were primarily characterized by PI interactions with D223^5.35^ and E297^6.58^, as previously described for MP1207 and MP1208.^14^ Instead, the positively charged amine groups were observed to move across the central binding cavity, forming PI interactions with E209^*ECL2*^ and E297^6.58^ located on opposite sides of the cavity (Table 4). Additionally, all three compounds contained aromatic moieties that engaged in π-cation interactions with K227^5.39^ (Table 4), a feature that may contribute to the disruption of interactions between K227^5.39^ and E297^6.58^, which is associated with the promotion of G protein bias.^16^ Notably, the most G protein-biased ligand, **KB07**, exhibited the highest frequency of interactions with K227^5.39^, underscoring the potential importance of this residue in G protein-biased signaling. It is important to note that the high standard errors of within the dynophore analysis stem from energy minima only being overcome in certain simulations, alowing unique binding modes in different simulations.

**Table 4:** Interaction Frequencies of the Upper Positively Charged Moiety of the **KB** Series Compounds Identified by *Dynophores* ^42–46^ ^a^

Compound	E209^*ECL2*^	D223^5.35^	E297^6.58^	K227^5.39^
**KB03**	14.6 ± 10.9%	0.0 ± 0.0%	88.0 ± 15.8%	2.4% ± 0.6%
**KB05**	57.4 ± 19.0%	10.3 ± 7.3%	43.9 ± 35.7%	12.4% ± 6.6%
**KB07**	24.8 ± 23.3%	11.1 ± 4.1%	15.2 ± 7.70%	32.5% ± 25.6%

a PI interactions were evaluated for the negative triad D223^5.35^, E209^*ECL2*^, and E297^6.58^, while aromatic interactions were evaluated for K227^5.39^. Interaction frequencies are shown as the percentage of frames where the interaction was observed. ± denotes the standard error calculated by treating each replica as a unique sample.

As ligand-induced G protein bias is expected to produce shifts in long-range allosteric coupling, we examined the allosteric communication paths in the different ligand-bound KOR systems using MDPath^47,48^ a toolkit that identifies allosterically stabilized networks by analyzing correlations of protein backbone movements between residues and linking them into communication paths. To identify baseline unbiased active state allosteric coupling, the KOR crytalized with MP1104 (PDB ID: 6B73 ^15^) was used, which was then compared to the KOR structures with docked **KB03**, **KB05**, and **KB07**. Additionally, we investigated the nalfurafine-bound KOR structure (PDB ID: 7YIT^16^) to see if we can identify consistent allosteric communication paths between bias KOR ligands, even with different putative ligand binding modes. Figure 3

**Figure 3:**
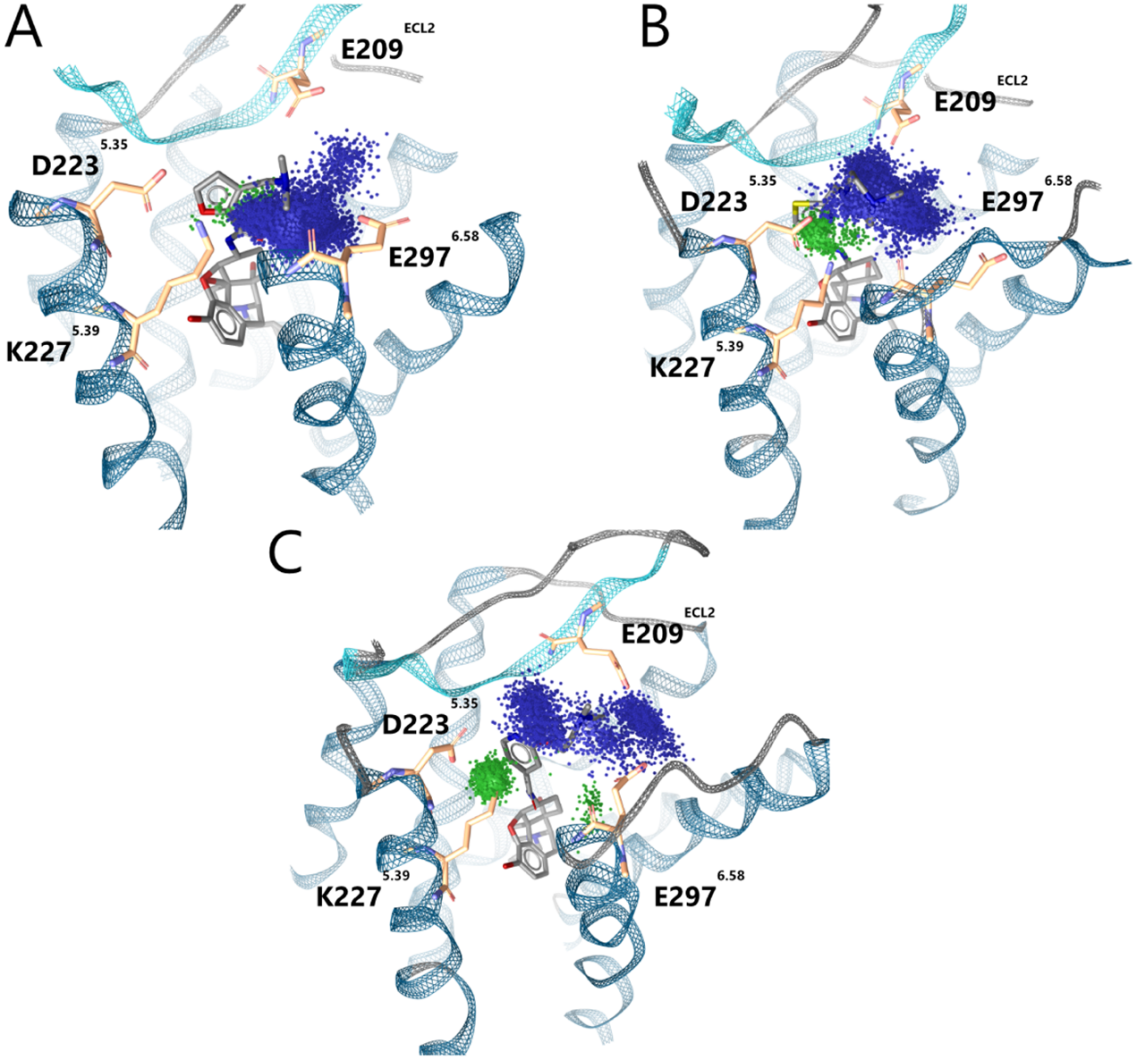
Dynophore analysis^42–46^ of PI (blue cloud) and aromatic (green cloud) interactions formed by the amide moieties of the three simulated **KB** series compounds docked into the KOR (PDB ID: 6B73 ^15^). (A) Dynophore of **KB03** bound to the KOR. PI interactions are limited to E297^6.58^ and E209*^ECL2^*, with only minimal aromatic interactions observed. (B) Dynophore of **KB05**. A more distributed PI interaction cloud is observed, and aromatic interactions are now clearly present. (C) Dynophore of **KB07**. The PI interaction cloud is split into 2 main interaction spaces, and overall less dense than **KB03** and **KB05**. However, an even more well-defined and stable aromatic interaction with K227^5.39^ is observed.

In simulations of the **KB** series and nalfurafine, we observed signaling paths involving K227^5.39^ (number of paths including K227^5.39^: **KB03**: 148; **KB05**: 54; **KB07**: 72; nalfurafine: 40). These paths traversed from the extracellular region of TM5, extended through TM6, and terminated in intracellular loop (ICL) 3, suggesting that K227^5.39^ plays a role in modulating ICL3 movement. Additionally, across all simulations, a strong path connection was consistently observed between F235^5.47^ (TM5) and F283^6.44^ (TM6), indicating a significant influence of TM5 on TM6 movement within these systems. In contrast, simulations with MP1104 revealed signaling paths along TM5 that largely bypass K227^5.39^ (number of paths including K227^5.39^: MP1104:10). This finding aligns with the study by El Daibani et al.^16^ which reported that disruption of the K227^5.39^–E297^6.58^ salt bridge by nalfurafine increases the distance between the extracellular ends of TM5 and TM6 and affects the positioning of ICL3, compared to non-G protein-biased ligands such as U50,488 or WMS-X600.^16^ Furthermore, mutations of K227^5.39^ to alanine showed an enhanced G_i3_ coupling, while ß-arrestin signaling seemed to be largely unaffected.^16^

A striking difference between MP1104 and the **KB** series compounds was the emergence of dominant allosteric communication paths along TM4 (Figure 4A,B). Interestingly, a similar TM4-centric signaling pattern was also observed with nalfurafine (Figure 4C), leading to the hypothesis that TM4 plays a critical role in G protein-biased signaling at the KOR. Although nalfurafine and the **KB** series compounds shared similar paths toward the intracellular region of the receptor, they appeared to induce these paths via distinct mechanisms within the binding pocket, consistent with their different binding modes. For the **KB** series ligands, allosteric signaling paths originated in ECL2 and followed two primary routes: either descending directly from ECL2 through TM4, or initially transitioning from ECL2 to TM3 before shifting to TM4 via W183^4.50^ (Figure 4D). W183^4.50^ appeared to act as a critical relay point, funneling paths through TM4 and likely playing a central role in facilitating TM4 movement. In contrast, TM4-associated paths induced by nalfurafine originated from a hydrophobic pocket formed by TM2 and TM3 beneath ECL2, where its furan moiety resides. Unlike the **KB** series, no signaling paths emerged from the extracellular end of TM4. Instead, paths from the hydrophobic pocket converged near the upper regions of TM2 and TM3, crossing over to TM4 via Y140^3.34^ (TM3) and N141^3.35^ (TM2), ultimately funneling through W183^4.50^ and continuing downward along TM4. Additionally, multiple paths were observed traversing TM2 and connecting to TM4 at various intracellular points, further suggesting a regulatory influence of TM2 on TM4 dynamics.

**Figure 4:**
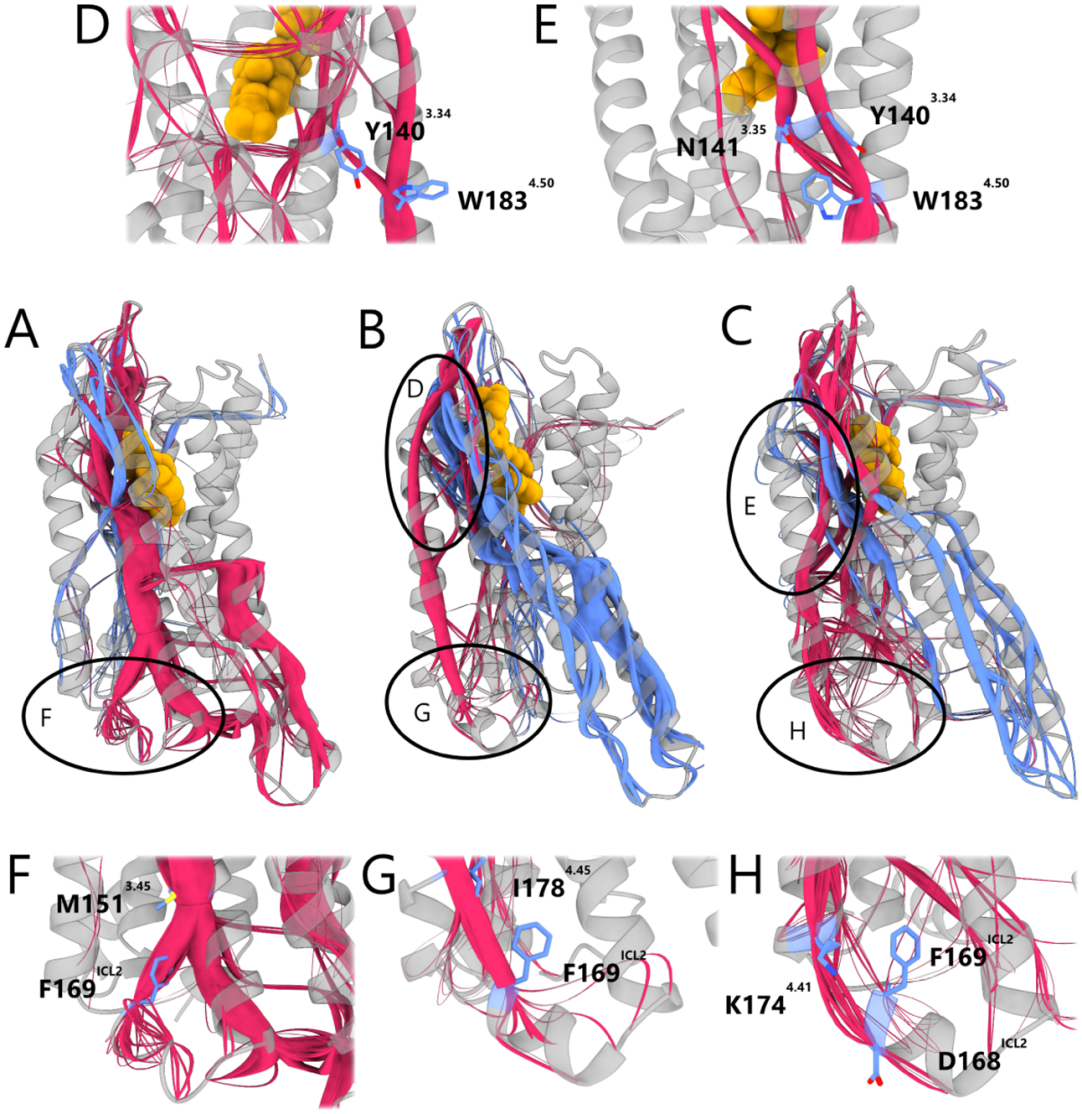
Allosteric communication paths in KOR bound to biased and unbiased ligands, computed with MDPath.^47,48^ (A–C) Full views of KOR bound to MP1104 (A) (PDB ID: 6B73 ^15^), **KB07** (B) (docked into PDB ID: 6B73 ^15^), and nalfurafine (**C**) (PDB ID: 7YIT^16^). Dominant paths are shown in red and blue; ligands in yellow. (D) **KB07**-bound KOR: TM4 paths originate from ECL2 via TM3/4, with TM3 paths merging at W183^4.50^–Y140^3.34^. (E) Nalfurafine-bound KOR: paths originate from the TM2/3 pocket near the furan moiety, reaching TM4 via Y140^3.34^ and N141^3.35^ to W183^4.50^. (F) MP1104-bound KOR: ICL2 paths emerge from TM3, passing M151^3.45^–F169^ICL2^; ICL2 lacks full helical structure. (G) **KB07**-bound KOR: ICL2 adopts a helical conformation, stabilized by paths from TM4, especially I178^4.45^ to F169^ICL2^. (H) Nalfurafine-bound KOR: ICL2 helix stabilized by TM4-originating paths, with key paths between K174^4.41^ and D168^ICL2^ near F169^ICL2^.

Notably, in all the biased ligand-bound KOR simulations, allosteric paths from TM4 ultimately converged in the ICL2, a region previously deemed important for G protein recognition.^16,49^ Across our analysis, ICL2 appeared to be modulated via F169^ICL2^, which acted as a key receiver of upstream allosteric communication paths. In the case of the unbiased ligand MP1104, paths toward ICL2 primarily originated from TM3, specifically via M151^3.45^–F169^ICL2^. In contrast, biased ligands predominantly displayed TM4-based paths: **KB** series channeled paths through I178^4.45^–F169^ICL2^, while nalfurafine routed its influence via K174^4.41^–D168^ICL2^, adjacent to F169^ICL2^. Moreover, the ICL2 region adopted a more pronounced helical secondary structure in the biased ligand-bound states, in contrast to the less ordered conformation observed with MP1104. These findings highlight the pivotal role of TM4-derived movements and ICL2 structural stabilization in mediating G protein-biased signaling in the analyzed protein ligand complexes.

To further investigate the discoveries on TM4 in a structural context, we revisited the experimentally determined structures of the MP1104-bound KOR (PDB ID: 6B73 ^15^) and the nalfurafine-bound KOR (PDB ID: 7YIT^16^). Given that TM4 had previously received limited attention in GPCR structural analyses, we aimed to determine whether intrinsic differences in TM4 were already present in these resolved structures. While most TM4 residues adopt similar positions, a striking difference can be observed in the orientation of W183^4.50^. In the nalfurafine-bound structure, W183^4.50^ is oriented toward TM2 and TM3, whereas in the MP1104-bound structure, it is positioned outward, away from the receptor core. This further solidifies the importance of W183^4.50^ as an allosteric switch for G protein biased signaling in the observed analyzed protein–ligand systems.

Ultimately, our findings indicate that G protein-biased signaling at the KOR is modulated through at least two distinct mechanisms. First, G protein biased signaling appears to be driven by an allosteric communication path involving TM4, which in turn influences the conformation of ICL2. This mechanism contrasts with the TM3-regulated ICL2 paths observed for the unbiased ligand MP1104. Second, the movement of ICL3, a critical region shaping the intracellular binding pocket of the GPCR, appears to be governed by interactions at the extracellular ends of TM5 and TM6, particularly the ionic lock formed between K227^5.39^ and E297^6.58^. These conclusions, supported by both Dynophore analysis^42–46^ and allosteric path mapping via MDPath^47,48^ highlight that G protein bias at the KOR is a multifaceted process, involving coordinated conformational changes and interactions across several receptor domains.

As the **KB** series not only exhibit bias but also display partial agonism at the KOR, we further investigated two representative ligands: **KB05**, which shows strong partial agonism with 41.1% G protein recruitment, and **KB03**,a more full agonist with 82.7% G protein activation. These were compared to the full agonist MP1104, the ligand-free KOR (apo), and the inverse agonist JDTic-bound KOR. To understand the effects induced by different ligand types, we explored the free energy landscapes of the receptor using enhanced sampling methods along the A100 score^50^ a metric that quantifies GPCR activation.

When comparing the agonists to the inverse agonist, we observed that the agonists energy minima are localized in regions with positive A100 scores, indicative of active conformational states, while the JDTic-bound KOR exhibited minima in regions with negative A100 scores, corresponding to inactive states. Interestingly, the ligand-free (apo) KOR also displayed a primary minimum in the active region. This observation is consistent with the reported high basal activity of the KOR.^51^ However, a local minimum was also present in the inactive state (A100 < 0). The same inactive state minimum was observed for the MP1104-bound receptor, however with a significantly higher free energy profile compared to the apo receptor, as expected for a full agonist stabilizing the active state (Figure 5).

**Figure 5:**
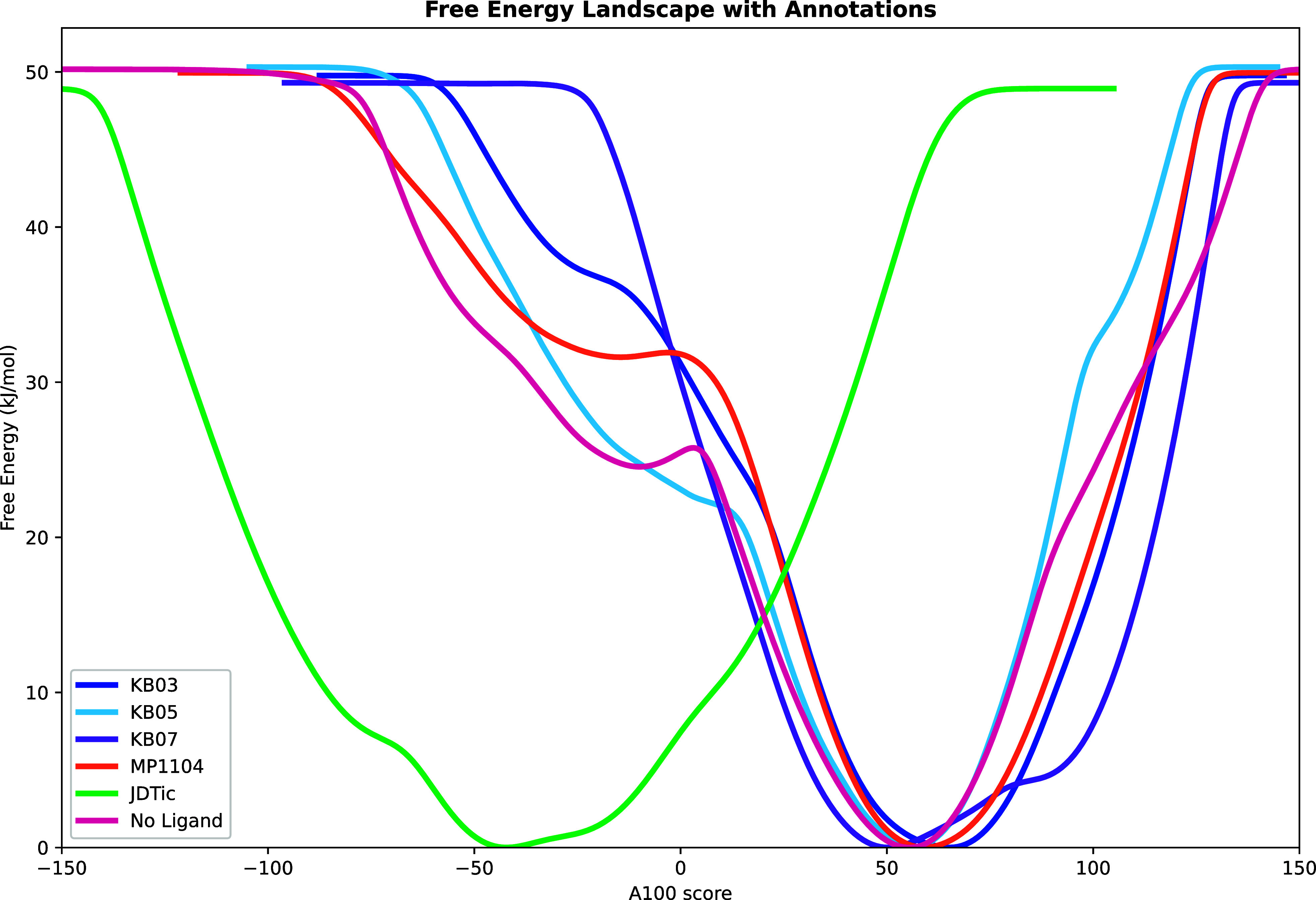
Free energy landscapes of the KOR bound to different ligands projected along the A100 activation score. The plots illustrate distinct conformational preferences induced by ligand binding. Agonists MP1104 (orange), **KB03** (dark blue), **KB05** (light blue), and **KB07** (purple) favor active states (A100 > 0), while the inverse agonist JDTic (green) stabilizes inactive conformations (A100 < 0). The apo receptor (red) also predominantly samples active states, consistent with its high basal activity.^51^ The **KB** series, exhibiting partial agonism, display a more continuous energy profile across the A100 score, lacking distinct barriers in the transition toward inactive conformations.

In the case of the **KB** series, the free energy landscapes revealed a more continuous transition between active and inactive states, lacking a distinct local minimum in the inactive region or a prominent energy maximum. This indicates that the conformational shift between these states does not require crossing a substantial energy barrier, which may explain the partial agonist activity of these ligands. Notably, **KB03**, which induces a higher degree of G protein recruitment, presents a free energy profile where the inactive state is considerably less accessible compared to **KB05**, as evidenced by elevated energy levels in those regions. Furthermore, **KB05** does not access the highly active conformational states reached by **KB03**, MP1104, or even the apo receptor, as indicated by the steep initial slope of its energy landscape toward higher A100 scores (Figure 5). This suggests that **KB05** not only fails to stabilize highly active receptor states but may also impede the receptor from adopting “hyper” active conformations.

An interesting phenomenon arises with **KB07**: the ligand appears locked against adopting fully inactive conformations, yet its free energy minimum lies in a less active region compared to other **KB** series analogs. This shift likely underlies its “partial activation” profile. Notably, we also detect a secondary local minimum toward more active conformations, resembling a metastable state that the **KB07**-bound KOR can adopt. This intermediate state may contribute not only to its partial agonism, but also to a potential bias profile, as it may influence signaling in a unique manner as well. Together, these observations illustrate how subtle scaffold modifications can reshape the conformational energy landscape, yielding distinct activation patterns.

The observed energetic differences between **KB03** and **KB05** may be mechanistically explained by the previous dynophore analysis. **KB05** interacted with a broader region of the extracellular binding pocket (Figure 3B), with a more spatially distributed dynophore involving interactions with E209^ECL2^, D223^5.35^, and E297^6.58^. This extended interaction network could impair the conformational changes necessary for full receptor activation. If the GPCR is conceptualized as a mechanical lever or scissor, **KB05** may act as a physical obstruction, limiting its range of motion and thereby preventing full engagement of the active state. In contrast, **KB03** occupies a more confined position near E297^6.38^ (Figure 3A), potentially reducing steric hindrance during receptor closure above the orthosteric pocket and enabling stabilization of active and “hyper” active conformations.

During ligand design we primarily focused on the KOR. However, given the scaffold, our ligands act as dual KOR/MOR agonists. This outcome was anticipated, since morphinans are known to engage all three classical opioid receptors,^52^ and the introduced basic functionality also complements the MOR extended binding site through interactions with E231^5.35^ on TM5 and D218^*ECL2*^ on ECL2. All compounds in the **KB** series displayed partial agonism and G protein bias at the MOR. To better understand their effects in this receptor, we analyzed the allosteric paths of **KB07** docked to the MOR.

Dynophore analysis revealed MOR binding patterns closely resembling those observed in the KOR: the morphinan core remained largely stable, while the charged substituent within the extended binding pocket engaged in spaced out interactions with TM5 and ECL2 through the positive charge. Notably, the TM4-based allosteric communication path identified in the KOR also appeared in the **KB07**-bound MOR (Figure 6A), suggesting that TM4 may contribute more generally to ECL2-mediated G protein bias across opioid receptors. An additional distinction was the emergence of multiple TM2-based communication paths modulating TM3 and TM4 (Figure 6A), reminiscent of the signaling paths observed in the nalfurafine-bound KOR (Figure 4C). Furthermore, the distribution of extended binding pocket interactions in the **KB07**-bound MOR Dynophore analysis were highly spread out (Figure 6B), resembling the **KB** series in the KOR (Figure 3), which may help explain the observed partial agonism at the MOR, as receptor closure appears similarly hindered.

**Figure 6:**
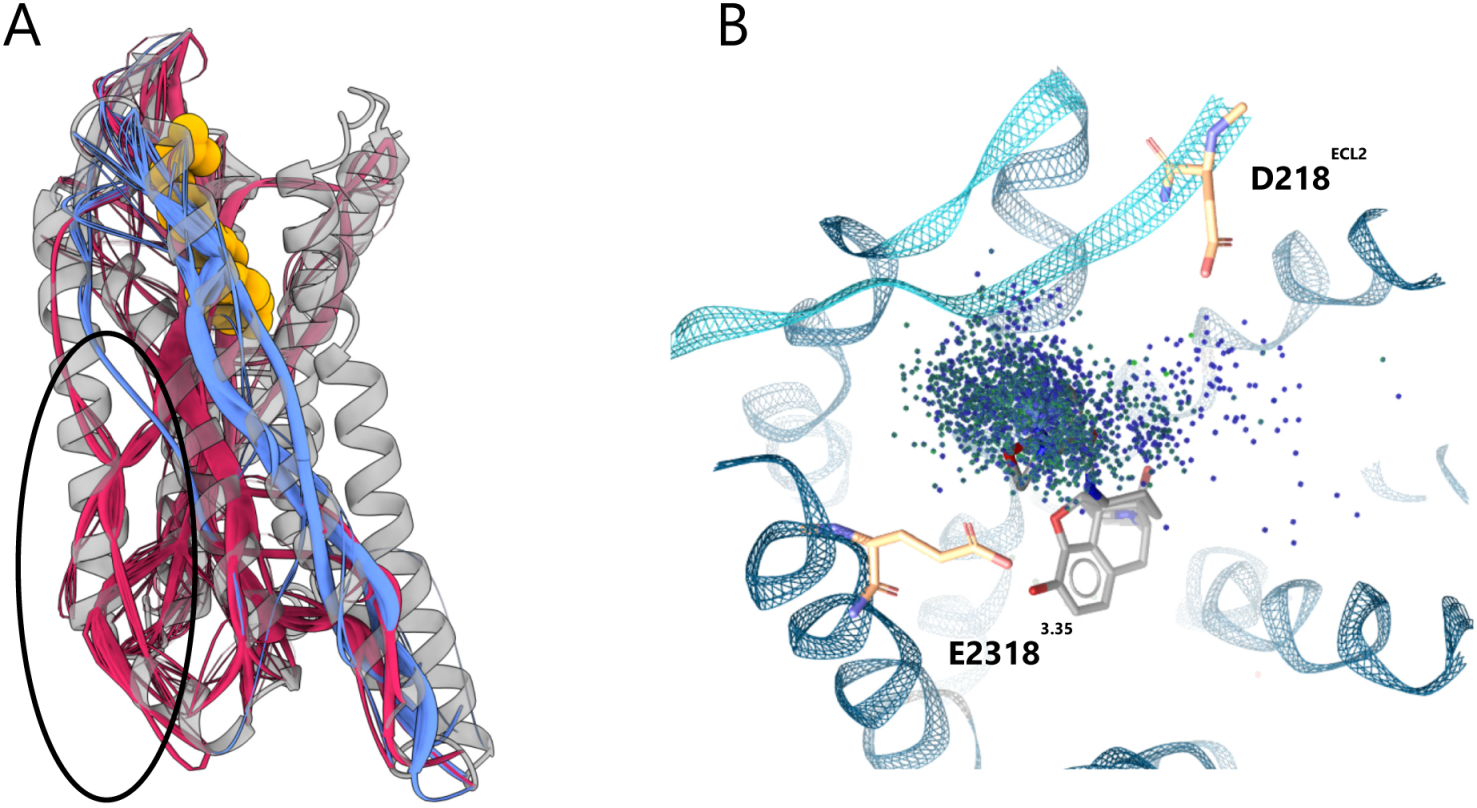
Analysis of **KB07**-bound MOR. (A) Overall view of the MOR in complex with **KB07** (PDB ID: 8EF6 ^53^). Allosteric communication paths are highlighted in red and blue. TM4- and TM2-based paths, which may contribute to G protein bias, are circled. (B) Dynophore representation of **KB07** in the MOR binding pocket. The distribution of key interactions is shown: blue dots indicate positive ionizable contacts, and green dots denote hydrogen bond donor interactions.

## Discussion and Conclusion

In this study, we present the rational design and exploration of G protein-biased KOR ligands using a novel series of dual-charged naltrexamine amides, marking a significant shift from traditional serendipitous discovery of biased ligands. Through a structure-based approach, we successfully identified eight ligands that preferentially activate G protein signaling pathways while minimizing ß-arrestin2 recruitment, a pharmacological profile associated with improved therapeutic properties. Beyond identifying new biased agonists, we also developed a molecular rationalization for KOR G protein-bias by integrating data from our novel ligand series with insights from the previously characterized ligands nalfurafine and MP1104. Our findings reveal distinct allosteric communication paths that inform the molecular mechanisms of functional selectivity. Furthermore, we propose underlying mechanisms contributing to the partial agonism observed in our ligand series, providing valuable insights into the modulation of receptor activity and further advancing our understanding of KOR pharmacology.

While our study primarily focuses on the **KB** series presented in Scheme 2, it is important to contextualize these compounds within the broader landscape of previously reported 6ß-naltrexamine amide analogs. Notably, several of our new analogs, including **KB04**, **KB07**, and**KB08**, are structurally related to prior scaffolds,^54^ with the key modification being the introduction of a basic functionality intended to modulate receptor interactions and potentially influence signaling bias. In our initial screening, ligand design focused on exploring G protein bias at KOR through this basic motifs interacting with the extended binding pocket. Although the designed ligands did not display KOR selectivity, as also observed with similar compounds,^14,54^ they enabled us to effectively probe the ECL2 and TM5/6 regions as potential sites for bias modulation. The observation that all of our ligands exhibited bias supports the notion that this approach can be a valuable route for designing biased ligands. However, the naltrexamine-derived scaffold is likely suboptimal for developing KOR-selective biased ligands, given its high affinity for both KOR and MOR. Moreover, all of our compounds acted as partial agonists, likely reflecting impaired conformational changes required for full receptor activation. Importantly, partial agonism can be advantageous in certain contexts, as such ligands have shown promise in the development of safer opioid drugs.^55^ For strategies aiming to generate fully biased full agonists, alternative mechanisms, such as those exploited by nalfurafine, may be preferable. Notably, there is also evidence suggesting a potential link between partial agonism and bias, further highlighting the relevance of our design approach.^24,56^

From a broader perspective, this compound series exemplifies four key strategies for developing safer opioid therapeutics: (I) G protein bias, to selectively activate desirable signaling pathways while minimizing adverse effects; (II) peripheral restriction, potentially facilitated by the presence of two positive charges, although this property has yet to be quantitatively assessed; (III) partial agonism, which may allow for more nuanced modulation of receptor activity and reduced side effects; (IV) a bifunctional approach targeting both the MOR and KOR, which could diminish the reward and addiction liability commonly associated with classical MOR agonists. Alternatively, selective KOR agonism may be pursued by optimizing ligands within this series to enhance the KOR selectivity, particularly for indications where the MOR activity is not desirable.

In the future, several avenues can be pursued to further advance the development of biased KOR ligands. One promising strategy involves replacing charged groups with hydrogen bond donors or acceptors to improve the bioavailability of the **KB** series, which currently serves primarily as a tool for studying signaling bias. Additionally, alternative strategies to mimic TM4-mediated signaling warrant further exploration, as they may offer novel insights into receptor regulation and open avenues for the development of next-generation pain therapeutics.

## Experimental Section

### General Procedures and Analytical Data for the Synthesized Compounds

All chemicals and solvents were purchased from commercial suppliers and used as received unless otherwise stated.

**Thin Layer Chromatography (TLC)** was carried out on TLC-plates from Merck (Silica gel 60, fluorescence indicator F254, layer thickness = 0.25 mm).

**LC-MS** was performed on an Agilent 1260 series HPLC system employing a DAD detector (at 300, 254, and 220 nm) and ELSD detector equipped with Agilent Technologies 6120 Quadrupole LC/MS in electrospray positive mode (ESI+). A Thermo Accucore RP-MS (30 × 2.1 mm, 2.6 μm) column was used with a flow rate 0.8 mL/min in combination with the following separation conditions: 0.1% formic acid in water (solvent A); 0.1% formic acid in ACN (solvent B); 5% B for 0.6 min, from 5 to 95% B in 6 min, 95% B for 1.4 min (stop point at 8 min). Data analysis was performed with ChemStation software (version 2.156.0.0) assisted by manual integration.

**Purification** of the compounds was performed with silica gel chromatography using a Biotage Isolera One apparatus with RediSepRf Columns from Teledyne Isco. Otherwise, preparative HPLC was performed on a Gilson PLC 2250 with a Macherey-Nagel VP 250/21 Nucleodur 100–7 C18Ec column (30 mL/min flow) or a Macherey-Nagel VP 250/10 Nucleodur 100–5 C18Ec column (5 mL/min flow).

^**1**^ **H NMR and** ^**13**^ **C NMR** spectra were recorded either on AV 300 MHz (295 K, 300 MHz, 75 MHz) or AV 600 MHz (300 K, 600 MHz, 151 MHz) spectrometers from Bruker. Chemical shifts are reported in ppm (δ) referenced to TMS (δ = 0.00 ppm), chemical shifts are reported in ppm (δ) are referenced to the residual nondeuterated solvent peak such as DMSO (^1^H NMR: 2.50 ppm) and CHCl_3_ (^1^H NMR: 7.26 ppm). NMR data were analyzed with MestReNova 14.2.2 software. Spin multiplicities were described as singlet (s), doublet (d), triplet (t), quartet (q), multiplet (m). Coupling constant (*J*) are given in Hz.

**HRMS** analyses were carried out using an Agilent Technologies 6530 Accurate Mass Q-ToF LC/MS spectrometer linked to Agilent Technologies HPLC 1260 Infinity II equipped with the column: Thermo Accucore RP-MS; particle size: 2.6 μm; dimension: 30 × 2.1 mm and using following gradient: eluent A: water with 0.1% FA; eluent B: acetonitrile with 0.1% FA. 0.00 min 95% A, 0.1 min 95% A, 1.0 min % A, 3.5 min stoptime, 1.3 min posttime; flow rate: 0.8 mlmin^–1^.

Purity and characterization of all final compounds was established by a combination of LC-MS, LC-HRMS and NMR analytical techniques. All tested compounds were found to be >95% purity by a combination of either LC-MS, LC-HRMS and ^1^H NMR.

#### Synthesis of 6ß-Naltrexamine

Naltrexone **1** (1 equiv), NH_2_OH–HCl (1.5 equiv), and NaOAc (2.5 equiv) were dissolved in absolute ethanol and the mixture was heated at reflux for 2.5 h and then concentrated to dryness under reduced pressure. Water was added and the mixture was made basic with K_2_CO_3_ and extracted with CHCl_3_. The organic layer was washed with brine, dried over Na_2_SO_4_, filtered and concentrated to give the intermediate **2** as a white solid (95%): LC-MS (ESI): *m*/*z* = 357 (MH+). Intermediate **2** (1 equiv) was dissolved in THF and transferred by cannula over 10 min to a solution of BH_3_.THF (1 M solution in THF, 18 equiv) held at 10 °C. A white precipitate formed and then slowly dissolved as the reaction was heated at reflux for 48 h. The solution was cooled to room temperature and water and 1 N KOH was added slowly. The solution was then reheated at reflux for 2 h. The solution was acidified with 2 M HCl to pH 2.5 and the solution was heated at reflux for an additional 2 h. The THF was removed under vacuum and the aqueous solution was basified (pH 8–9) by addition of K_2_CO_3_. The mixture was extracted with CHCl_3_ and the unified layers were dried over Na_2_SO_4_, filtered and concentrated. The resulting oil was purified by flash chromatography on SiO_2_ (elution with CH_3_CN/MeOH/NH_4_OH, 25/5/1, v/v/v) providing 6ß-naltrexamine **3** (diastereomer) as a colorless amorphous solid.^29^ The title compound was synthesized according to the procedure discribed above from commercially available naltrexone hydrochloride (200 mg, 0.529 mmol, 1 equiv) as a white-yellow solid (100 mg, 57%): *R*_*f*_ = 0.17; ^1^H NMR (CDCl_3_ with two drops of CD_3_OD) d 6.61 (d, *J* = 8.1 Hz, 1H), 6.49 (d, *J* = 8.1 Hz, 1H), 4.17 (d, *J* = 7.5 Hz, 1H), 3.39–0.45 (20 H); LC-MS (ESI): *m*/*z* = 343.2 (M+H)+.

#### General Procedure^57^

The respective carboxylic acid derivative (2 equiv) was dissolved in anhydrous dimethylformamide (DMF). The mixture was then cooled at 0 °C and 1-ethyl-3­(3-(dimethylamino)­propyl)­carbodiimide hydrochloride (EDCI, 3 equiv), 1-hydroxy-7azabenzotriazole (HOAt, 3 equiv), 4 Å molecular sieves, and triethylamine (TEA, 8.0 equiv) under N_2_ atmosphere were added. The mixture was kept at 0 °C for 15 min and then a solution of 6β-naltrexamine hydrochloride **3** (1 equiv) in DMF was added dropwise. The resulting mixture was warmed up to ambient temperature and the reaction was stirred for 5 h. Then, the reaction mixture was concentrated to remove DMF under reduced pressure to yield the crude intermediate **4**. The crude **4** was dissolved in MeOH and K_2_CO_3_ (3 equiv) was added. The mixture was stirred for 16 h at room temperature and then concentrated under reduced pressure to remove methanol. The crude product was purified by reversed-phase preparative HPLC using a gradient of 5% to 99% acetonitrile with 0.1% TFA (5% ACN + TFA 0.1% for 5 min, gradient 5–99% ACN + TFA 0.1% for 35 min, 99% ACN + TFA 0.1% for 5 min, stop point at 45 min) to obtain the final compounds **KB01-KB08**.

#### **KB01**: *N*-((4r,4as,7r,7ar,12bs)-3-(Cyclopropylmethyl)-4a,9-dihydroxy-2,3,4,4a,5,6,7,7a-octahydro-1H-4,12-methanobenzofuro­[3,2-e]­isoquinolin-7-yl)-3-((dimethylamino)­methyl)­thiophene-2-carboxamide

The title compound was synthesized according to the general procedure 2. The synthesized 6β-naltrexamine (10 mg, 0.024 mmol, 1 equiv) was combined with the commercially available 3-((dimethylamino)­methyl)­thiophene-2-carboxylic acid (8.92 mg, 0.048 mmol, 2 equiv), EDCl (13.85 mg, 0.072 mmol, 3 equiv), HOAt (9.83 mg, 0.072 mmol, 3 equiv) and TEA (19.49 mg, 0.192 mmol, 8 equiv) in DMF (1 mL) followed by basic hydrolysis with K_2_CO_3_ (9.98 mg, 0.072 mmol, 3 equiv) in MeOH (3 mL) to give the title compound as a white solid (1.6 mg, 13%). The crude product was purified by reversed-phase preparative HPLC (C18 column, 5–99% ACN/water with 0.1% TFA). LC-MS (8 min 5% to 95% acetonitrile with 0.1% FA run – ESI) retention time (*t*_*R*_) 2.035 min; *m*/*z* = 510.2 (M+H)^+^; m/2z = 255.7 (M+2H)^2+^. ^1^H NMR (300 MHz, CD_3_OD) δ 7.89 (d, *J* = 5.1 Hz, 1H), 7.28 (d, *J* = 5.0 Hz, 1H), 6.77 (m, 2H), 4.75 (d, *J* = 8.0 Hz, 1H), 4.62 (s, 2H), 4.53–4.36 (m, 2H), 3.98 (d, *J* = 4.7 Hz, 1H), 3.46–3.31 (m, 2H), 3.26–3.05 (m, 2H), 2.92 (s, 6H), 2.80–2.52 (m, 2H), 2.16–1.96 (m, 1H), 1.88–1.66 (m, 2H), 1.62 (t, *J* = 12.1 Hz, 2H), 1.11 (q, *J* = 5.2 Hz, 1H), 0.84 (t, *J* = 6.2 Hz, 1H), 0.80–0.68 (m, 1H), 0.53 (p, *J* = 5.0 Hz, 2H). ^13^C NMR (151 MHz, CD_3_OD) δ 163.02, 142.34, 136.80, 135.19, 132.65, 131.90, 131.22, 129.11, 120.53, 119.63, 118.08, 69.95, 62.96, 57.39, 54.79, 53.97, 51.84, 46.27, 42.31, 41.84, 29.92, 27.39, 23.07, 5.42, 4.74, 1.99. HRMS (ESI): *m*/*z* calc = 510, 2421 found 510.2412 (M+H)^+^.

#### **KB02**: *N*-((4R,4aS,7R,7aR,12bS)-3-(Cyclopropylmethyl)-4a,9-dihydroxy-2,3,4,4a,5,6,7,7a,11,12-decahydro-1*H*-4,12-methanobenzofuro­[3,2-e]­isoquinolin-7-yl)-5-(Pyrrolidin-1-ylmethyl)­furan-2-carboxamide

The title compound was synthesized according to the general procedure 2. The synthesized 6β-naltrexamine (10 mg, 0.024 mmol, 1 equiv) was combined with the commercially available 5-(pyrrolidin-1-ylmethyl)­furan-2-carboxylic acid (9.40 mg, 0.048 mmol, 2 equiv), EDCl (13.85 mg, 0.072 mmol, 3 equiv), HOAt (9.83 mg, 0.072 mmol, 3 equiv), and TEA (19.49 mg, 0.192 mmol, 8 equiv) in DMF (1 mL) followed by basic hydrolysis with K_2_CO_3_ (9.98 mg, 0.072 mmol, 3 equiv) in MeOH (3 mL) to give the title compound as a white solid (3.4 mg, 27%). The crude product was purified by reversed-phase preparative HPLC (C18 column, 5–99% ACN/water with 0.1% TFA). LC-MS (2.5 min, 5–95% acetonitrile with 0.1% FA run – ESI) retention time (*t*_*R*_) 0.902 min; *m*/*z* = 520.3 (M+H)^+^; *m*/2*z* = 260.7 (M+2H)^2+^. ^1^H NMR (300 MHz, CD_3_OD) δ 7.29–7.11 (m, 1H), 6.84 (d, *J* = 3.5 Hz, 1H), 6.79–6.66 (m, 2H), 4.84–4.75 (m, 1H), 4.54 (s, *J* = 2.2 Hz, 2H), 3.95 (d, *J* = 5.7 Hz, 1H), 3.90–3.77 (m, 2H), 3.62 (t, *J* = 6.8 Hz, 2H), 3.46–3.39 (m, 1H), 3.32 (t, 2H), 3.22 (d, *J* = 6.0 Hz, 1H), 3.18–3.03 (m, 1H), 3.01–2.82 (m, 1H), 2.81–2.56 (m, 2H), 2.16–2.01 (m, 4H), 2.00–1.91 (m, 1H), 1.85–1.71 (m, 1H), 1.69–1.55 (m, 2H), 1.29 (s, 1H), 1.09 (m, *J* = 12.5 Hz, 1H), 0.82 (m, 1H), 0.81–0.66 (m, 1H), 0.53 (p, *J* = 5.1 Hz, 2H). ^13^C NMR (151 MHz, CD_3_OD) δ 165.80, 156.40, 147.16, 129.34, 120.44, 119.60, 118.27, 116.52, 114.88, 114.60, 90.40, 69.92, 62.99, 57.36, 56.07, 53.66, 51.35, 49.34, 46.25, 29.76, 27.49, 23.29, 23.04, 22.65, 5.42, 4.75, 1.96. HRMS (ESI): *m*/*z* calc = 520.2806, found 520.2807 (M+H)^+^.

#### **KB03**: *N*-((4R,4aS,7R,7aR,12bS)-3-(Cyclopropylmethyl)-4a,9-dihydroxy-2,3,4,4a,5,6,7,7a-octahydro-1*H*-4,12-methanobenzofuro­[3,2-e]­isoquinolin-7-yl)-3-((dimethylamino)­methyl)­furan-2-carboxamide

The title compound was synthesized according to the general procedure 2. The synthesized 6β-naltrexamine (10 mg, 0.024 mmol, 1 equiv) was combined with the commercially available 3-((dimethylamino)­methyl)­furan-2-carboxylic acid (8.15 mg, 0.048 mmol, 2 equiv), EDCl (13.85 mg, 0.072 mmol, 3 equiv), HOAt (9.83 mg, 0.072 mmol, 3 equiv), and TEA (19.49 mg, 0.192 mmol, 8 equiv) in DMF (1 mL) followed by basic hydrolysis with K_2_CO_3_ (9.98 mg, 0.072 mmol, 3 equiv) in MeOH (3 mL) to give the title compound as a white-grayish solid (3.1 mg, 26%). The crude product was purified by reversed-phase preparative HPLC (C18 column, 5–99% ACN/water with 0.1% TFA). LC-MS (8 min, 5–95% acetonitrile with 0.1% FA run – ESI) retention time (*t*_*R*_) 2.385 min; *m*/*z* = 494.2 (M+H)^+^; *m*/2*z* = 247.7 (M+2H)^2+^. ^1^H NMR (300 MHz, CD_3_OD) δ 7.79 (d, *J* = 1.8 Hz, 1H), 6.77 (d, *J* = 2.1 Hz, 3H), 4.76 (d, *J* = 8.0 Hz, 1H), 4.58–4.33 (m, 2H), 4.04–3.82 (m, 2H), 3.42 (d, *J* = 6.1 Hz, 1H), 3.40–3.34 (m, 1H), 3.22 (d, *J* = 5.9 Hz, 1H), 3.18–3.04 (m, 2H), 2.93 (d, *J* = 4.1 Hz, 1H), 2.90 (d, *J* = 5.4 Hz, 6H), 2.68 (pd, *J* = 12.9, 3.9 Hz, 2H), 2.16–1.93 (m, 1H), 1.81 (d, *J* = 14.1 Hz, 1H), 1.76–1.56 (m, 2H), 1.35 (dt, *J* = 14.5, 7.3 Hz, 1H), 1.11 (td, *J* = 7.1, 3.7 Hz, 1H), 0.92–0.79 (m, 1H), 0.79–0.65 (m, 1H), 0.53 (p, *J* = 5.0 Hz, 2H). ^13^C NMR (151 MHz, CD_3_OD) δ 159.21, 145.11, 144.96, 142.34, 141.79, 120.81, 120.52, 119.63, 118.11, 114.12, 90.61, 69.94, 62.95, 57.38, 52.18, 50.84, 46.27, 42.03, 41.78, 29.89, 27.40, 23.28, 23.05, 5.42, 4.75, 1.98. HRMS (ESI): *m*/*z* calc = 494.2649, found 494.2649 (M+H)^+^.

#### **KB04**: 4-((Benzyl­(methyl)­amino)­methyl)-*N*-((4R,4aS,7R,7aR,12bS)-3-(cyclopropylmethyl)-4a,9-dihydroxy-2,3,4,4a,5,6,7,7a-octahydro- 1*H*-4,12-methanobenzofuro­[3,2-e]­isoquinolin-7-yl)­benzamide

The title compound was synthesized according to the general procedure 2. The synthesized 6β-naltrexamine (10 mg, 0.024 mmol, 1 equiv) was combined with the commercially available 4-((benzyl­(methyl)­amino)­methyl)­benzoic acid (12.29 mg, 0.048 mmol, 2 equiv), EDCl (13.85 mg, 0.072 mmol, 3 equiv), HOAt (9.83 mg, 0.072 mmol, 3 equiv), and TEA (19.49 mg, 0.192 mmol, 8 equiv) in DMF (1 mL) followed by basic hydrolysis with K_2_CO_3_ (9.98 mg, 0.072 mmol, 3 equiv) in MeOH (3 mL) to give the title compound as a white-yellowish solid (1.8 mg, 12.90%). The crude product was purified by reversed-phase preparative HPLC (C18 column, 5–99% ACN/water with 0.1% TFA). LC-MS (8 min 5% to 95% acetonitrile with 0.1% FA run – ESI) retention time (*t*_*R*_) 2.806 min; *m*/2*z = 580.3* (M+H)^+^; *m*/2*z* = 290.7 (M+2H)^2+^. ^1^H NMR (600 MHz, CD_3_OD) δ 8.08–7.98 (m, 2H), 7.81–7.60 (m, 3H), 7.57 (d, *J* = 7.8 Hz, 2H), 7.53–7.45 (m, 2H), 6.84–6.72 (m, 2H), 4.03–3.95 (m, 1H), 3.92 (ddd, *J* = 12.8, 8.3, 4.7 Hz, 1H), 3.67 (d, *J* = 3.6 Hz, 1H), 3.46–3.36 (m, 1H), 3.26–3.19 (m, 1H), 3.17–3.11 (m, 1H), 2.98–2.88 (m, 1H), 2.76 (s, 4H), 2.68 (dd, *J* = 13.1, 4.4 Hz, 1H), 2.13–2.00 (m, 2H), 1.80 (td, *J* = 14.1, 3.9 Hz, 2H), 1.72–1.63 (m, 2H), 1.31 (s, 3H), 1.18–1.09 (m, 1H), 0.96–0.90 (m, 1H), 0.90–0.83 (m, 1H), 0.78 (dt, *J* = 8.0, 4.8 Hz, 1H), 0.55 (tp, *J* = 9.4, 4.9 Hz, 2H). ^13^C NMR (151 MHz, CD_3_OD) δ 167.22, 161.93, 156.90, 142.73, 134.07, 130.97, 130.22, 129.52, 129.40, 129.23, 90.62, 70.11, 63.12, 59.91, 59.28, 57.49, 56.19, 52.09, 46.39, 29.91, 29.49, 27.65, 23.18, 16.00, 5.56, 4.89, 2.10. HRMS (ESI): *m*/*z* calc = 580.3170, found 580.3194 (M+H)^+^.

#### **KB05**: *N*-((4R,4aS,7R,7aR,12bS)-3-(Cyclopropylmethyl)-4a,9-dihydroxy-2,3,4,4a,5,6,7,7a-octahydro-1*H*-4,12-methanobenzofuro­[3,2-e]­isoquinolin-7-yl)-2-((4-methylpiperazin-1-yl)­methyl)­thiazole-4-carboxamide

The title compound was synthesized according to the general procedure 2. The synthesized 6β-naltrexamine (10 mg, 0.024 mmol, 1 equiv) was combined with the commercially available 2-((4-methylpiperazin-1-yl)­methyl)­thiazole-4-carboxylic acid (11.62 mg, 0.048 mmol, 2 equiv), EDCl (13.85 mg, 0.072 mmol, 3 equiv), HOAt (9.83 mg, 0.072 mmol, 3 equiv) and TEA (19.49 mg, 0.192 mmol, 8 equiv) in DMF (1 mL) followed by basic hydrolysis with K_2_CO_3_ (9.98 mg, 0.072 mmol, 3 equiv) in MeOH (3 mL) to give the title compound as a white-yellowish solid (5.9 mg, 43%). The crude product was purified by reversed-phase preparative HPLC (C18 column, 5–99% ACN/water with 0.1% TFA). LC-MS (8 min 5–95% acetonitrile with 0.1% FA run – ESI) retention time (*t*_*R*_) 2.418 min; *m*/*z* = 566.3 (M+H)^+^; *m*/2*z* = 283.7 (M+2H)^2+^. ^1^H NMR 600 MHz, MeOD) δ 8.18 (s, 1H), 6.80– 6.68 (m, 2H), 4.81 (dd, *J* = 7.9, 1.1 Hz, 1H), 4.04 (s, 2H), 3.95 (d, *J* = 5.9 Hz, 2H), 3.52 (s, 2H), 3.43–3.33 (m, 2H), 3.24–3.09 (m, 4H), 3.09 (s, 1H), 2.99–2.86 (m, 3H), 2.73 (td, *J* = 12.8, 3.9 Hz, 1H), 2.68–2.60 (m, 4H), 2.13–2.03 (m, 1H), 1.78 (dt, *J* = 13.9, 3.2 Hz, 1H), 1.72 (dt, *J* = 12.3, 4.1 Hz, 1H), 1.64 (ddd, *J* = 20.3, 13.5, 3.5 Hz, 2H), 1.11 (ddt, *J* = 12.8, 6.0, 3.8 Hz, 1H), 0.84 (dtd, *J* = 9.8, 5.2, 2.8 Hz, 1H), 0.79–0.71 (m, 1H), 0.57–0.47 (m, 2H). ^13^C NMR (151 MHz, CD_3_OD) δ 171.13, 163.33, 150.81, 143.84, 143.14, 130.77, 126.13, 121.86, 120.96, 119.75, 91.98, 71.40, 64.37, 59.02, 58.78, 54.91, 52.77, 51.05, 47.67, 43.51, 31.21, 28.91, 24.70, 24.44, 6.83, 6.15, 3.38. HRMS (ESI): *m*/*z* calc = 566.2796, found 566.2819 (M+H)^+^.

#### **KB06**: *N*-((4R,4aS,7R,7aR,12bS)-3-(Cyclopropylmethyl)-4a,9-dihydroxy-2,3,4,4a,5,6,7,7a-octahydro-1*H*-4,12-methanobenzofuro­[3,2-e]­isoquinolin-7-yl)-3-(2-(dimethylamino)­ethyl)-2-oxo-2,3-dihydro-1*H*-benzo­[d]­imidazole-5-carboxamide

The title compound was synthesized according to the general procedure 2. The synthesized 6β-naltrexamine (10 mg, 0.024 mmol, 1 equiv) was combined with the commercially available 3-(2-(dimethylamino)­ethyl)-2-oxo-2,3-dihydro-1H-benzo­[d]­imidazole-5-carboxylic acid (12 mg, 0.048 mmol, 2 equiv), EDCl (13.85 mg, 0.072 mmol, 3 equiv), HOAt (9.83 mg, 0.072 mmol, 3 equiv), and TEA (19.49 mg, 0.192 mmol, 8 equiv) in DMF (1 mL) followed by basic hydrolysis with K_2_CO_3_ (9.98 mg, 0.072 mmol, 3 equiv) to give the title compound as a yellowish solid (6.5 mg, 47%). The crude product was purified by reversed-phase preparative HPLC (C18 column, 5–99% ACN/water with 0.1% TFA). LC-MS (8 min 5–95% acetonitrile with 0.1% FA run – ESI) retention time (*t*_*R*_) 0.255 min; *m*/*z* = 574.3 (M+H)^+^; *m*/2*z* = 287.8 (M+2H)^2+^. ^1^H NMR (600 MHz, CD_3_OD) δ 7.76 (d, *J* = 1.6 Hz, 1H), 7.71 (dd, *J* = 8.2, 1.6 Hz, 1H), 7.20 (d, *J* = 3.1 Hz, 1H), 6.82–6.70 (m, 2H), 3.98 (d, *J* = 5.9 Hz, 1H), 3.93 (s, 6H), 3.64 (t, *J* = 5.9 Hz, 1H), 3.61 (t, *J* = 5.9 Hz, 4H), 3.44–3.35 (m, 2H), 3.26–3.19 (m, 1H), 3.19–3.12 (m, 1H), 2.98–2.88 (m, 2H), 2.75 (td, *J* = 12.7, 3.8 Hz, 1H), 2.68 (td, *J* = 13.0, 4.5 Hz, 1H), 2.14–2.05 (m, 1H), 1.85–1.75 (m, 2H), 1.67 (ddd, *J* = 19.6, 13.5, 3.3 Hz, 2H), 1.14 (td, *J* = 7.9, 4.1 Hz, 1H), 0.89–0.82 (m, 1H), 0.77 (ddt, *J* = 12.7, 8.2, 4.2 Hz, 1H), 0.60–0.49 (m, 2H). ^13^C NMR (151 MHz, CD_3_OD) δ 168.37, 167.12, 161.23, 160.99, 155.91, 142.46, 141.74, 132.88, 129.50, 124.40, 123.45, 115.59, 108.77, 107.08, 90.68, 70.00, 63.00, 57.37, 55.59, 51.91, 51.20, 46.26, 42.56, 35.94, 29.78, 27.54, 23.37, 23.06, 5.43, 4.75, 1.98. HRMS (ESI): *m*/*z* calc = 574.3024, found 574.3034 (M+H)^+^.

#### **KB07**: *N*-((4R,4aS,7R,7aR,12bS)-3-(Cyclopropylmethyl)-4a,9-dihydroxy-2,3,4,4a,5,6,7,7a-octahydro-1*H*-4,12-methanobenzofuro­[3,2-e]­isoquinolin-7-yl)-2-(2-(dimethylamino)­ethoxy)­isonicotinamide

The title compound was synthesized according to the general procedure 2. The synthesized 6β-naltrexamine (10 mg, 0.024 mmol, 1 equiv) was combined with the commercially available 2-(2-(dimethylamino)­ethoxy)­isonicotinic acid (10.12 mg, 0.048 mmol, 2 equiv), EDCl (13.85 mg, 0.072 mmol, 3 equiv), HOAt (9.83 mg, 0.072 mmol, 3 equiv), and TEA (19.49 mg, 0.192 mmol, 8 equiv) in DMF (1 mL) followed by basic hydrolysis with K_2_CO_3_ (9.98 mg, 0.072 mmol, 3 equiv) in MeOH (3 mL) to give the title compound as a pale yellow solid (7.4 mg, 57%). The crude product was purified by reversed-phase preparative HPLC (C18 column, 5–99% ACN/water with 0.1% TFA). LC-MS (8 min, 5–95% acetonitrile with 0.1% FA run – ESI) retention time (*t*_*R*_) 2.831 min; *m*/*z* = 535.5 (M+H)^+^; *m*/2*z* = 268.2 (M+2H)^2+^. ^1^H NMR (600 MHz, MeOD) δ 8.32 (ddd, *J* = 14.8, 5.3, 0.8 Hz, 1H), 7.42–7.39 (m, 1H), 7.29–7.27 (m, 1H), 6.80–6.72 (m, 2H), 4.79 (d, *J* = 8.0 Hz, 1H), 4.74–4.70 (m, 2H), 3.95 (d, *J* = 6.0 Hz, 1H), 3.94 (s, 1H), 3.87 (ddd, *J* = 12.8, 7.9, 4.9 Hz, 1H), 3.65–3.60 (m, 2H), 3.42–3.34 (m, 2H), 3.19 (dd, *J* = 19.6, 6.2 Hz, 1H), 3.12 (dd, *J* = 12.3, 5.6 Hz, 1H), 3.00 (d, *J* = 3.7 Hz, 6H), 2.93–2.86 (m, 1H), 2.73 (td, *J* = 12.8, 3.9 Hz, 1H), 2.65 (td, *J* = 13.0, 4.7 Hz, 1H), 2.07 (qd, *J* = 13.1, 2.7 Hz, 1H), 1.80 (dt, *J* = 13.7, 3.1 Hz, 1H), 1.77–1.71 (m, 1H), 1.65 (td, *J* = 14.0, 3.1 Hz, 2H), 1.16–1.07 (m, 1H), 0.84 (dtd, *J* = 9.8, 5.1, 2.7 Hz, 1H), 0.75 (ddt, *J* = 12.5, 8.1, 4.4 Hz, 1H), 0.56–0.47 (m, 2H). ^13^C NMR (151 MHz, MeOD) δ 167.37, 164.46, 148.72, 146.60, 143.62, 143.01, 130.59, 120.88, 119.54, 116.39, 110.42, 91.61, 71.22, 64.24, 61.33, 58.63, 57.74, 53.27, 43.84, 30.99, 28.76, 24.37, 24.31, 6.69, 6.01. HRMS (ESI): *m*/*z* calc = 535.2815, found 535.2877 (M+H)^+^.

#### **KB08**: 5-(((4R,4aS,7R,7aR,12bS)-3-(Cyclopropylmethyl)-4a,9-dihydroxy-2,3,4,4a,5,6,7,7a-octahydro-1*H*-4,12-methanobenzofuro­[3,2-e]­isoquinolin-7-yl)­carbamoyl)-1,2,3,4-tetrahydroisoquinolin-2-ium Triflate

The Boc-protected precursor of the title compound was synthesized according to the general procedure 2. The synthesized 6β-naltrexamine (10 mg, 0.024 mmol, 1 equiv) was combined with the commercially available 2-(tert-butoxycarbonyl)-1,2,3,4-tetrahydroisoquinoline-5-carboxylic acid (13.35 mg, 0.048 mmol, 2 equiv), EDCl (13.85 mg, 0.072 mmol, 3 equiv), HOAt (9.83 mg, 0.072 mmol, 3 equiv) and TEA (19.49 mg, 0.192 mmol, 8 equiv) in DMF (1 mL) followed by basic hydrolysis with K_2_CO_3_ (9.98 mg, 0.072 mmol, 3 equiv) in MeOH (3 mL) to give the Boc-protected desired molecule. The final title compound was obtained by performing a Boc-deprotection reaction, stirring the obtained Boc-protected intermediate in a mixture of DCM/TFA (3:1) to give the title compound as a white solid (6.72 mg, 55%). The crude product was purified by reversed-phase preparative HPLC (C18 column, 5–99% ACN/water with 0.1% TFA). LC-MS (8 min 5% to 95% acetonitrile with 0.1% FA run – ESI) retention time (*t*_*R*_) 0.261 min; *m*/*z* = 502.3 (*M* + *H*)^+^; *m*/2*z* = 251.7 (*M* + 2*H*)^2+^. ^1^H NMR (300 MHz, CD_3_OD) δ 7.46 (d, *J* = 7.0 Hz, 1H), 7.38 (t, *J* = 7.8 Hz, 1H), 7.35–7.31 (m, 1H), 7.17 (dq, *J* = 16.3, 8.0 Hz, 2H), 6.77 (d, *J* = 2.0 Hz, 2H), 4.68 (d, *J* = 8.0 Hz, 1H), 4.41 (s, 2H), 3.95 (d, *J* = 5.7 Hz, 1H), 3.84 (dt, *J* = 12.7, 6.7 Hz, 1H), 3.49 (t, *J* = 6.3 Hz, 2H), 3.43–3.33 (m, 1H), 3.29–3.05 (m, 2H), 2.89 (dd, *J* = 13.5, 7.5 Hz, 1H), 2.80–2.54 (m, 2H), 2.32 (s, 1H), 2.00 (t, *J* = 12.2 Hz, 1H), 1.80 (d, *J* = 11.4 Hz, 2H), 1.65 (t, *J* = 13.2 Hz, 2H), 0.95–0.70 (m, 2H), 0.52 (d, *J* = 5.1 Hz, 2H). ^13^C NMR (75 MHz, CD_3_OD) δ 170.09, 142.41, 141.82, 136.26, 129.58, 129.40, 128.98, 128.53, 128.39, 127.82, 126.81, 126.49, 124.92, 120.49, 119.61, 118.19, 90.47, 69.96, 62.88, 57.32, 51.61, 46.24, 44.36, 41.21, 29.82, 27.44, 23.34, 23.04, 22.78, 5.42, 4.77, 1.96. HRMS (ESI): *m*/*z* calc = 502.2700, found 502.2730 (*M* + *H*)^+^.

### Pharmacology

#### Drugs and Chemicals

Radioligands [^3^ *H*]­U69,593, [^3^ *H*]­DAMGO, [^3^ *H*]­DPDPE, and [^35^ *S*]­GTPγS were purchased from PerkinElmer (Boston, MA, USA) or Revvity (Waltham, MA, USA). Guanosine diphosphate (GDP), GTPγS, U69,593, DPDPE, DAMGO, naltrindole, 6β-naltrexamine, tris­(hydroxymethyl) aminomethane (Tris), 2-[4-(2-hydroxyethyl)­piperazin-1-yl]­ethanesulfonic acid (HEPES), bovine serum albumin (BSA), phosphate buffered saline (PBS), and cell culture media and supplements were obtained from Sigma-Aldrich Chemicals (St. Louis, MO, USA). Morphine hydrochloride was obtained from Gatt-Koller GmbH (Innsbruck, Austria). Nalfurafine was obtained from MedChemExpress (Sollentuna, Sweden). HS665 and HS666 were provided by Dr. Helmut Schmidhammer, and were prepared as previously described (Spetea et al., 2012). PZM21 was obtained from TargetMol EU GmbH (Linz, Austria). TRV130 was obtained from InvivoChem (Libertyville, IL, USA). All other chemicals were of analytical grade and obtained from standard commercial sources. Test compounds (**KB01-KB08**), as salts, were prepared as 10 mM stocks in water, and further diluted to working concentrations in the appropriate medium.

#### Cell Culture and Cell Membrane Preparation

CHO cells stably expressing the human opioid receptors, KOR, MOR, or DOR (CHO-hKOR, CHO-hMOR and CHO-hMOR cell lines), were kindly provided by Dr. Lawrence Toll (SRI International, Menlo Park, CA). U2OS cells stably transfected with human KOR and ß-arrestin2 genes (U2OS-hKOR-ß-arrestin2) and U2OS cells stably transfected with human MOR and β-arrestin2 genes (U2OS-hMOR-β-arrestin2) were purchased from DiscoveRx, Birmingham, UK. The CHO-hKOR cell line was maintained in DMEM supplemented with 10% FBS, 0.1% penicillin/streptomycin, 2 mM l-glutamine FBS (10%), and Geneticin (400 μg/mL). The CHO-hMOR and CHO-hDOR cell lines were maintained in DMEM)/Ham’s F-12 medium supplemented with 10% FBS, 0.1% penicillin/streptomycin, 2 mM l-glutamine and Geneticin (400 μg/mL). U2OS cells stably coexpressing the human opioid receptors and the enzyme acceptor (EA) tagged β-arrestin2 fusion protein were cultured in Minimum Essential Medium (MEM) culture medium supplemented with 10% FBS, 0.1% penicillin/streptomycin, 2 mM l-glutamine, Geneticin (500 μg/mL), and hygromycine (250 μg/mL). All cell cultures were maintained at 37 °C in 5% CO_2_ humidified air.

Membranes from CHO-hOR cells were prepared as previously described.^30^ Briefly, cells grown at confluence were removed from the culture plates by scraping, homogenized in 50 mM Tris-HCl buffer (pH 7.7) using a Dounce glass homogenizer, and then centrifuged once and washed by an additional centrifugation at 27,000g for 15 min at 4 °C. The final pellet was resuspended in 50 mM Tris-HCl buffer (pH 7.7), and stored at −80 °C until use. Protein content of cell membrane preparations was determined by the method of Bradford using BSA as the standard.^58^

#### Radioligand Competitive Binding Assays

Competitive binding assays were conducted on human opioid receptors stably transfected into CHO cells according to the published procedures.^30^ Binding assays were performed using [^3^ *H*]­U69,593 (1 nM), [^3^ *H*]­DAMGO (1 nM) or [^3^ *H*]­DPDPE (1 nM), for labeling KOR, MOR, or DOR, respectively. Nonspecific binding was determined using 10 μM of the unlabeled counterpart of each radioligand. Assays were performed in 50 mM Tris-HCl buffer (pH 7.4) in a final volume of 1 mL. Cell membranes (15–20 μg) were incubated with test compounds and the appropriate radioligand for 60 min at 25 °C. After incubation, reactions were terminated by rapid filtration through Whatman GF/C glass fiber filters. Filters were washed three times with 5 mL of ice-cold 50 mM Tris-HCl buffer (pH 7.4) using a Brandel M24R cell harvester (Gaithersburg, MD, USA). Radioactivity retained on the filters was counted by liquid scintillation counting using a Beckman Coulter LS6500 (Beckman Coulter Inc., Fullerton, CA, USA). All experiments were performed in duplicate, and repeated at least three times with independently prepared samples.

#### [^35^ *S*]­GTPγS Functional Assay

Binding of [^35^ *S*]­GTPγS to membranes from CHO cells stably expressing the human KOR or MOR was conducted according to the published procedures.^30,38^ Cell membranes (10–15 μg) in Buffer A (20 mM HEPES, 10 mM MgCl_2_ and 100 mM NaCl, pH 7.4) were incubated with 0.05 nM [^35^ *S*]­GTPγS, 10 μM GDP and test compounds in a final volume of 1 mL, for 60 min at 25 °C. Nonspecific binding was determined using 10 μM GTPγS, and the basal binding was determined in the absence of test compound. Samples were filtered over Whatman GF/B glass fiber filters using a Brandel M24R cell harvester (Brandel, Gaithersburg, MD, USA). Radioactivity retained on the filters was counted by liquid scintillation counting using a Beckman Coulter LS6500 (Beckman Coulter Inc., Fullerton, CA, USA). All experiments were performed in duplicate, and repeated at least three times with independently prepared samples.

#### β-Arrestin2 Recruitment Assay

The measurement of KOR and MOR stimulated β-arrestin2 recruitment was performed using the PathHunterβ-arrestin2 assay (DiscoveRx, Birmingham, UK) according to the published procedures.^30,33^ U2OS cells that stably coexpressed the human KOR or MOR and the enzyme acceptor (EA) tagged β-arrestin2 fusion protein (U2OS-hKOR-β-arrestin2 or U2OS-hMOR-β-arrestin2 cells) were seeded in cell plating medium into 384-well white plates (Greiner Bio-One, Kremsmünster, Austria) at a density of 2000 or 5000 cells, respectively in 20 μL per well, and maintained at 37 °C for 48 h (KOR assay) or 24 h (MOR assay). After incubation with various concentrations of the test compounds for 180 min (KOR assay) or 90 min (MOR assay) at 37 °C, the detection mix was added, and incubation was continued for an additional 60 min at room temperature. Chemiluminescence was measured with the Varioscan Lux microplate reader (Thermo Scientific, Waltham, MA, USA). All experiments were performed in duplicate, and repeated at least three times with independently prepared samples.

#### Data Analysis

Experimental data were analyzed and graphically processed using the GraphPad Prism 9.0 Software (GraphPad Prism Software Inc., San Diego, CA). Data are presented as means ± SEM. The inhibition constant (*K*_*i*_, nM), potency (*EC*_50_, nM), and efficacy (*E*_*max*_ %) values were determined from concentration–response curves by nonlinear regression analysis. The *K*_*i*_ values were determined by the method of Cheng and Prusoff.^59^ In the [^35^ *S*]­GTPγS functional and β-arrestin2 recruitment assays, concentration–response data were normalized to the maximal stimulation of the reference KOR or MOR agonists, U69,593 or DAMGO, respectively. Biased factors were calculated based on the Black and Leff operational method^40^ and as described.^38^

### Computational Methods

#### Protein Structure Preparation

The models of the MP1104 and nalfurafine-bound active state KOR and active state MOR were initially retrieved from the protein data bank (PDB)^60^ under the accession codes 6B73 ^15^ 7YIT^16^ 8EF6.^53^ They were subsequently loaded and processed in MOE 2020.02.^61^ The receptor and ligand were isolated by removing additional structures such as nanobodys and G proteins including the worse resolved dimer chain of 6B73 (chain B). The structures were restored to the human wild-type sequence by mutating resdiues according to the UniProt-Databank^62^ entry P41145 and P35372. Using the loop modeler within MOE^61^ missing structures were reconstructed (6B73: ECL2, ECL3, ICL3; 7YIT: ECL2, ECL3, ICL1, ICL3) and missing atoms were added. A water molecule was added between TM5 and TM6 by placed according to a solved water molecule in the active state MOR (PDB: 5C1M^63^), as this water molecule has shown significance within the KOR-ligand binding.^18^ The geometric properties of the receptor structures were optimized by careful energy minimization of atom clashes and Ramachandran outliers in MOE.^61^ Finally, structures were protonated at 300 K and a pH of 7.0 using Protonate3D^64^ in MOE^61^ and aligned according to the OPM database.^65^

#### Protein–Ligand Docking

Small molecule docking experiments of 6β-naltrexamine derivatives, MP1208, and 6’-GNTI to the KOR model (PDB ID: 6B73 ^15^) and of the 6β-naltrexamine derivatives to the MOR (PDB ID: 8EF6 ^53^) were conducted in Gold v5.2.^66^ The receptor model was prepared as described above. The binding site of the KOR was defined by a 30Å sphere around the carboxylic carbon atom of the side chain of D138^3.32^ (γC atom) and restricted to solvent accessible surface. A binding site for the MOR was defined similarly around D149^3.32^. The modeled water molecule was kept for docking. For each ligand 15 docking poses were generated. To promote the correct morphinan placement a constraint was applied to ensure an ionic interaction between the carboxylic side chain carbon atom of D138^3.32^ and the morphinan scaffold amine group nitrogen (maximum distance of 5.5 Å). Diverse docking solutions were obtained to consider for the flexibility of the substituents, i.e., the minimum Root mean square deviation (RMSD) between two docking poses of the same ligand need to be 1.5 Å. The search efficiency was increased to 200% as recommended for flexible ligands. Pyramidal nitrogen atoms were allowed to flip during the pose generation process. GoldScore^66^ was applied for generation and ranking of the docking poses. The docking poses were energy-minimized within the protein–ligand complex using the MMFF94 force filed,^67–73^ implemented in LigandScout 4.4 ^25^ before evaluation.

#### Pharmacophore Virtual Screening

The 3D pharmacophore was derived from the generated docking pose of MP1208 ^14^ in the prepared KOR structure (PDB ID: 6B73 ^15^) using LigandScout 4.4.^25^ The pharmacophore was used to screen a database of amino acids generated by filtering Enamine REAL reagents (version 2021, retrieved from https://enamine.net/) consisting of ∼126*k* compounds. The filtering process was conducted in KNIME v4.5.2 ^27^ using the RDKit^28^ nodes. The library was then prepared using idbgen implemented in LigandScout 4.4 ^25^ with base settings. Subsequently, the 3D pharmacophore virtual screening of the database was conducted using iScreen implemented in LigandScout 4.4.^25^

#### Molecular Dynamics Simulations

MD simulations were prepared using CHARMM-GUI^74^ and run using OpenMM.^75^ The receptor termini were capped, pH was set to 7.0, and the disulfide bridge between C131^3.25^ and C210^*ECL2*^ was specified. Ligand force fields were generated based on SDF files isolated from the respective structure/docking pose and generated with the CHARMM general force field.^76^ The receptor was placed in a POPC lipid membrane placed according to the OPM database^65^ and the system was solvated in a TIP3P water box with 10 Å padding. Charges were neutralized with 0.15 M NaCl. MD simulations were computed on the GPUs of the computer cluster at the molecular design lab at the Institute of Pharmacy at Freie Universität Berlin. Simulations were run using CHARMM36m force fields.^77^ The systems were minimized and equilibrated according to the CHARMM-GUI^74^ equilibration protocol. Production runs were conducted in an NPγT ensemble with a surface tension of 0 dyn/cm, a pressure of 1 bar and a temperature of 303.15 K with a Monte Carlo barostat and a 0.002 ps integrator. All systems were simulated in triplicates. The MP1104 and nalfurafine bound KOR systems were simulated for 200 ns per replicate comprising 1000 frames. The MP1208, **KB03**, **KB05** and **KB07**-bound KOR were simulated for 400 ns (2000 frames) per replica, as the systems contain docked ligands and the receptor was meant to adjust accordingly for adequate path analysis.

#### MD Trajectory Analysis

Captured MD trajectories were initially loaded into VMD^78^ for visual analysis, and exported without water, ions and lipids for further analysis. Additionally, truncated trajectories of the **KB03**, **KB05**, and **KB07**-bound KOR were exported containing only the last 1000 frames to ensure that the system was able to adapt to the docked ligand. Protein–ligand interactions over the course of the full MD trajectories were analyzed using Dynophores.^42–46^ Allosteric communication paths were analyzed using MDPath.^47,48^ All trajectories were analyzed with MDPath^47,48^ base setting, and additional ligand-based path analysis was conducted based on interactions identified with Dynophores^42–46^ (Table S1). The **KB03**, **KB05**, and **KB07**-bound KOR trajectories were analyzed in their total and truncated versions.

#### Metadynamics Simulations

MD simulations were prepared using CHARMM-GUI^74^ and run with GROMACS 2024.3 ^79^ using OPES^80^ Metadynamics within the PLUMED library^81^ version 2.9.3.^82^ The **KB03**, **KB05**, and MP1104-bound systems were prepared as described for unbiased MD simulations. The unliganded (apo) KOR system was prepared by removing MP1104 from the prepared structure (PDB ID: 6B73 ^15^). The JDTic-bound inactive KOR structure was prepared from the PDB entry 6VI4.^83^ A receptor monomer was isolated by removing all structures except chain A in MOE 2020.2.^61^ The rest of the preparation was done as for described for the other systems. Systems were minimized and equilibrated according to the protocols provided by CHARMM-GUI.^74^ For the production runs six walkers were run with an 0.002 ps integrator for 100 ns resulting in a total simulation time of 600 ns. OPES Metadynamics were biased using the A100 Score^50^ as a colective variable. The BARRIER parameter was set to 50 kJ/mol and the deposition PACE was set to 500 simulation steps. Energy calculations and reweighting were performed based on Invernizzi et al. (2020)^84^ with the free energy derived from the system’s temperature and the probability density function obtained from the reweighted biases of the metadynamics simulation.

### Coding and Writing

Coding assistance was provided by *ChatGPT* [OpenAI, L.L.C., San Francisco, CA], *Claude* [Anthropic PBC, San Francisco, CA], and *GitHub Copilot* [GitHub Inc., San Francisco, CA]. Grammar, spelling, punctuation, and tone were edited with *ChatGPT* [OpenAI, L.L.C., San Francisco, CA], *Claude* [Anthropic PBC,San Francisco, CA], and *DeepL* [DeepL SE, Cologne, Germany].

## Supplementary Material













## Data Availability

PDB structures are available from the Protein Data Bank (RCSB.org):^60^ 6B73,^15^ 6VI4,^83^ and 7YIT.^16^

## Notes

Supporting Information:concentration–response curves (KOR and MOR); KOR concentration-dependent G protein and ß-arrestin2 activation; MOR concentration-dependent G protein and ß-arrestin2 activation; binding of **KB** series and reference ligands at the human opioid receptors; dynophore interaction analysis; analysis of **MP1208**; analytical characterization of active compounds **KB01**–**KB08** (PDF)
KOR in modeled complex with **KB03** (PDB)
KOR in modeled complex with **KB05** (PDB)
KOR in modeled complex with **KB07** (PDB)
KOR in modeled complex with **MP1208** (PDB)
Molecular Formula Strings (CSV)

## Abbreviations

DAMGO: [d-Ala2,N-Me-Phe4,Gly-ol5]­enkephalin
DOR: δ-opioid receptors
DPDP: [D-Pen2,D-Pen5]­encephalin
EA: enzyme acceptor
ECL: extracellular loop
GPCR: G protein-coupled receptor
ICL: intracellular loop
KOR: κ-opioid receptor
MD: molecular dynamics
MOR: μ-opioid receptor
PDB: protein data bank (rcsb.org)
SAR: structure–activity relationships
TM: transmembrane
U69,593: *N*-methyl-2-phenyl-*N*-[(5R,7S,8S)-7-(pyrrolidin-1-yl)-1-oxaspiro­[4.5]­dec-8-yl]­acetamide
[^35^ *S*]­GTPγS: guanosine-5′-O-(3-[^35^ *S*]­thio)-triphosphate
