# Supplementary material for: Mechanistic Insights into G Protein-Biased κ‑Opioid Receptor Signaling Using Dual-Charged Naltrexamine Amides
Source: J Med Chem. 2026 Feb 5;69(4):3833–51. doi: 10.1021/acs.jmedchem.5c02135 (PMC12951437; doi:10.1021/acs.jmedchem.5c02135)

Supporting Information
Mechanistic Insights into G Protein-Biased κ-Opioid Receptor Signaling Using Dual-Charged Naltrexamine Amides

Niklas Piet Doering,[†,‡] Kristina Puls,[†,‡] Marta Diceglie,[¶] Anja Meraner,[§] Axel Hentsch,[¶] Siriwat Hongnak,[§] Armin Wurzer,[§] Helmut Schmidhammer,[§] Mariana Spetea,[*,§] Marc Nazare,[*,¶] and Gerhard Wolber[*,‡]

†*equally contributed to this work*

‡*Department of Biology, Chemistry and Pharmacy, Institute of Pharmacy, Molecular Design Group, Königin-Luisestr. 2+4, 14195 Berlin, Germany*

¶*Medicinal Chemistry, Leibniz-Forschungsinstitut für Molekulare Pharmakologie (FMP), Campus Berlin Buch, Robert-Roessle-Str. 10, 13125 Berlin, Germany*

§*Department of Pharmaceutical Chemistry, Institute of Pharmacy and Center for Molecular Biosciences Innsbruck (CMBI), University of Innsbruck, Innrain 80-82, 6020 Innsbruck, Austria*

E-mail: mariana.spetea@uibk.ac.at; nazare@fmp-berlin.de; gerhard.wolber@fu-berlin.de

Phone: +43 512 507 58277; +49 30 94793 581; +49 30 838 52686. Fax: +43 512 507 58299; +49 30 94063 084; +49 30 838 452686

Contents

Concentration-Response Curves (KOR and MOR) S3

KOR Concentration Dependent G Protein and ß-Arrestin2 Activation S4

MOR Concentration Dependent G Protein and ß-Arrestin2 Activation S5

Binding of KB series and reference ligands at the human opioid receptors S6

Dynophore Interaction Analysis S7

Analysis of MP1208 .... S8

Analytical Characterization of Active Compounds **KB01-KB08** S9

Concentration-Response Curves (KOR and MOR)

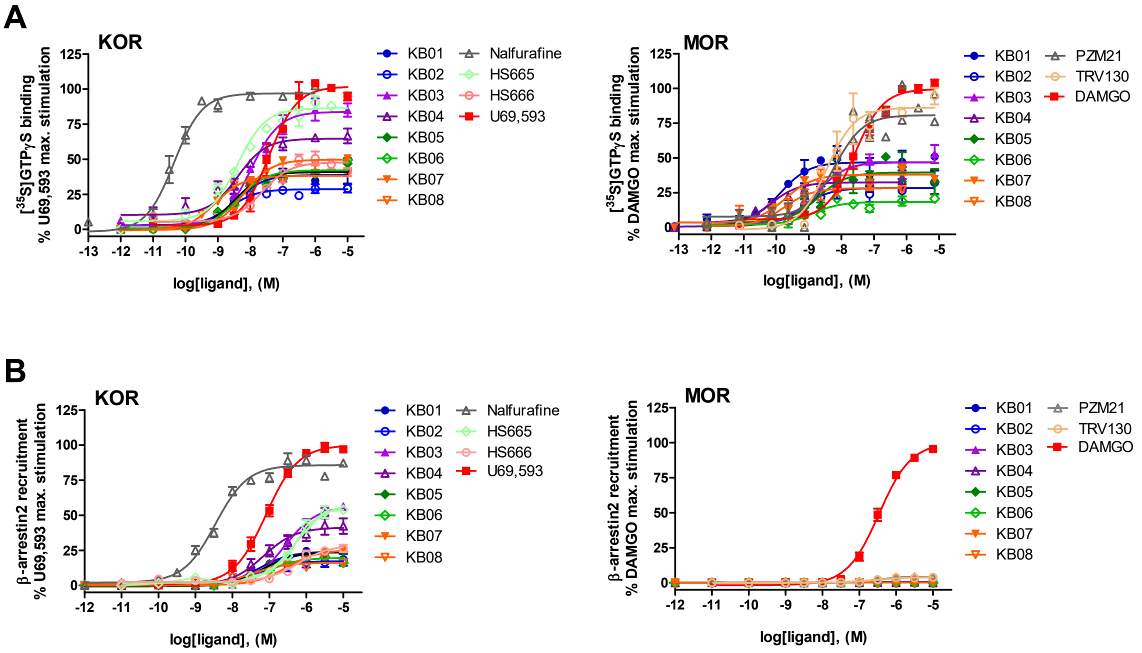

Figure S1: Concentration-response curves of (A) G protein activation (the [35S]GTPγS binding assay) and (B) β-arrestin2 recruitment (the PathHunter β-arrestin2 recruitment assay) at the human KOR and MOR by **KB01 - KB08**, reference KOR agonists nalfurafine, HS665, HS665 and U69,593 (KOR), and reference MOR agonists PZM21,TRV130 and DAMGO. Percentage stimulation is presented relative to the maximum stimulation of U69,593 and DAMGO, as reference KOR and MOR agonists, respectively. Values represent means ± SEM of at least three independent experiments.

KOR Concentration Dependent G Protein and ß-Arrestin2 Activation

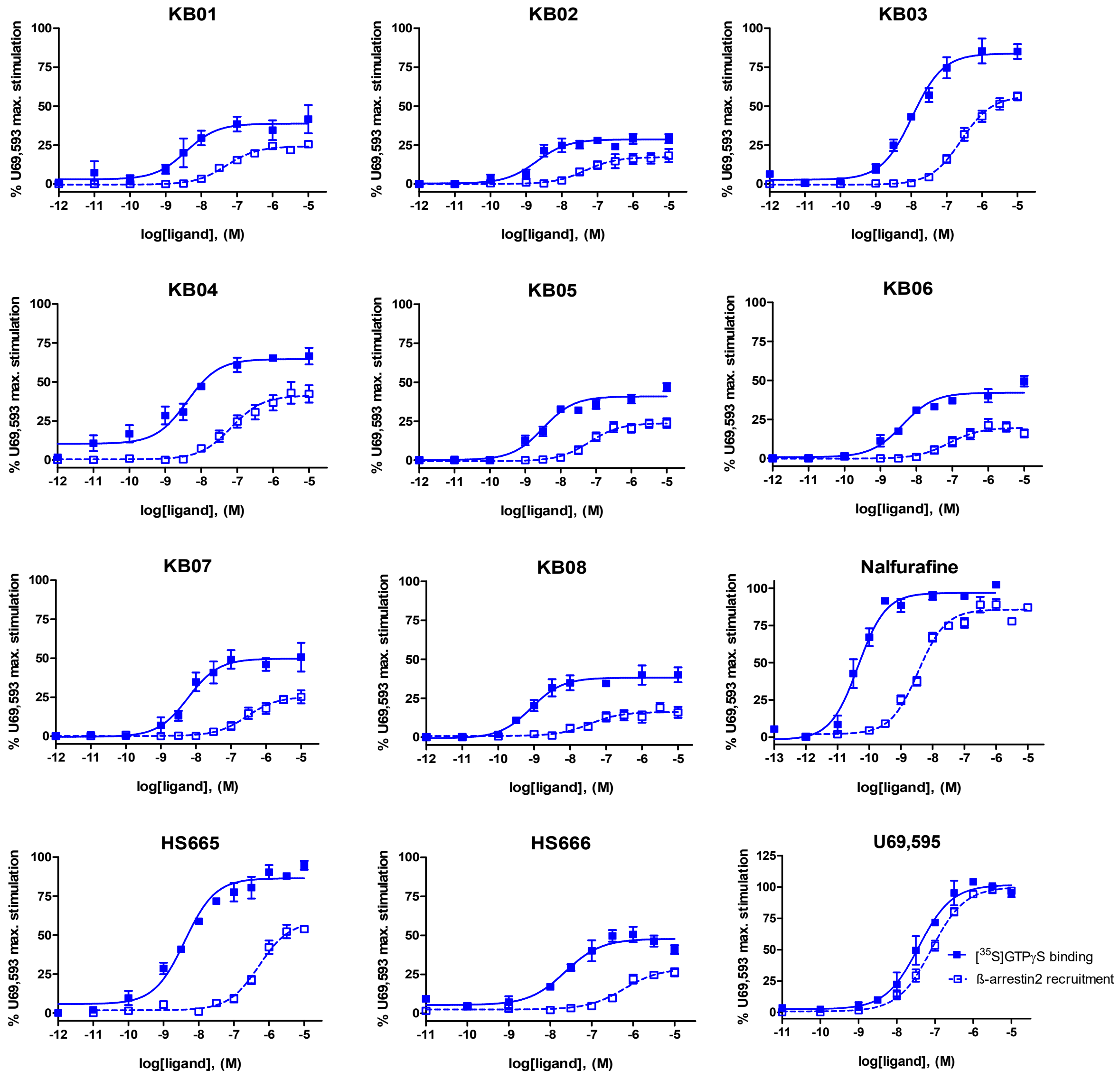

Figure S2: Concentration-dependent curves of G protein activation (the [35S]GTPγS binding assay) and β-arrestin2 recruitment (the PathHunter β-arrestin2 recruitment assay) at the human KOR by **KB01 - KB08**, and the reference KOR ligands, nalfurafine, HS665, HS666 and U69,593. Percentage stimulation is presented relative to maximum stimulation of U69,593. Values represent means ± SEM of at least three independent experiments.

MOR Concentration Dependent G Protein and ß-Arrestin2 Activation

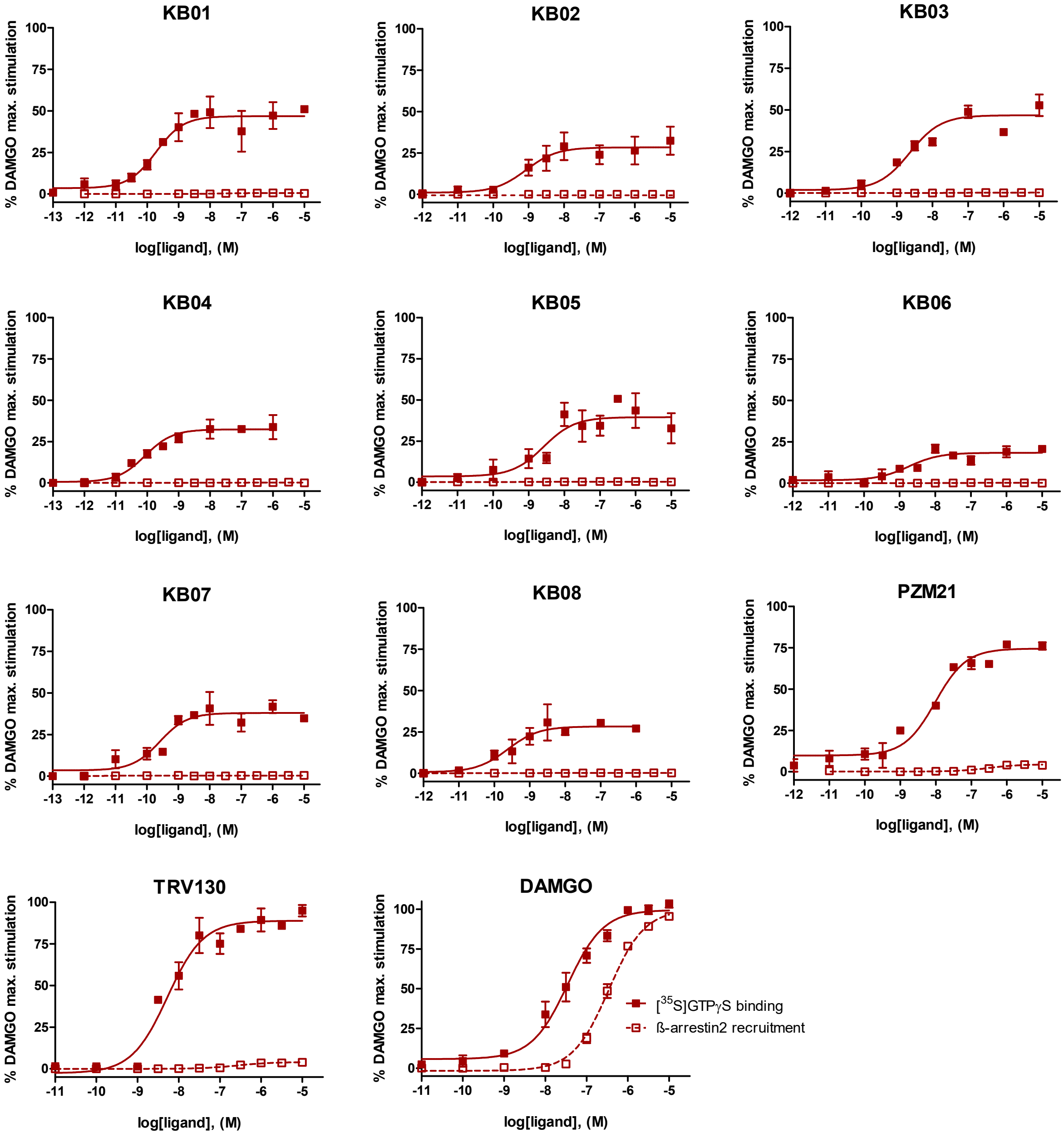

Figure S3: Concentration-dependent curves of G protein activation (the [35S]GTPγS binding assay) and β-arrestin2 recruitment (the PathHunter β-arrestin2 recruitment assay) at the human MOR by **KB01 - KB08**, and the reference MOR ligands, PZM21, TRV130 and DAMGO. Percentage stimulation is presented relative to maximum stimulation of DAMGO. Values represent means ± SEM of at least three independent experiments.

Binding of KB series and reference ligands at the human opioid receptors

Table S1: Binding of KB series and reference ligands at the human opioid receptors

	pIC_{50}[a]		
**Ligand**	**KOR**	**MOR**	**DOR**
**KB01**	9.13 ± 0.47	9.98 ± 0.06	7.58 ± 0.15
**KB02**	8.74 ± 0.25	9.07 ± 0.01	7.03 ± 0.10
**KB03**	8.77 ± 0.15	8.67 ± 0.04	6.55 ± 0.29
**KB04**	8.91 ± 0.18	9.88 ± 0.17	7.44 ± 0.15
**KB05**	9.06 ± 0.10	9.25 ± 0.06	6.99 ± 0.17
**KB06**	8.70 ± 0.11	9.62 ± 0.07	7.31 ± 0.14
**KB07**	8.79 ± 0.04	9.14 ± 0.05	6.92 ± 0.23
**KB08**	9.19 ± 0.20	9.52 ± 0.07	6.84 ± 0.17
**6ß-Naltrexamine**	8.75 ± 0.14	8.71 ± 0.08	6.64 ± 0.12
**Nalfurafine**	9.53 ± 0.18	-	-
**Morphine**	-	8.26 ± 0.02	-
**Naltrindole**	-	-	9.43 ± 0.10

[a] Determined in radioligand competitive binding assays with CHO cell membranes stably expressing the one of the recombinant human opioid receptors. - denotes not tested. Values are means ± SEM of at least three independent experiments.

Dynophore Interaction Analysis

Table S2: Interaction residues used for MDPath ligand-based path tracking.[a]

Ligand	Hydrophobic	HBA	HBD	PI	Aromatic
**MP1104**	108, 124, 135, 212, 287, 316, 320	139	138	138	-
**Nalfurafine**	108, 124, 134, 135, 142, 230, 287, 294, 316, 320	139, 211	138, 139, 291	138	-
**KB03**	108, 142, 214, 287, 290, 294, 316, 320	139, 312	138, 139, 291, 297	138, 209, 297	227
**KB05**	108, 142, 214, 212, 230, 287, 294, 316, 320	139, 227	138, 139	138, 209, 223, 297	227
**KB07**	108, 142, 212, 214, 226, 287, 290, 294, 312, 316, 320	139, 211, 212, 227, 312, 320	138, 209, 211, 212, 291, 297	138, 209, 223, 297	227

[a] Interactions were determined with Dynophores built in LigandScout. Only interactions that occured in min 10% of frames were taken into account for ligand-based path tracking.

Analysis of MP1208

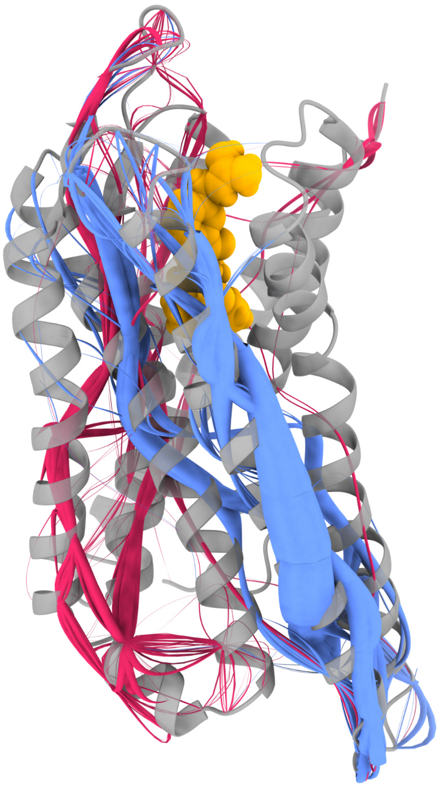

Figure S4: Allosteric path analysis of the MP1208-bound (docked) KOR (PDB ID: 6B73). Allosteric paths were analyzed using MDPath based on the last 1000 frames to ensure adaptation of the receptor to the docked ligand.

The allosteric communication path analysis of the MP1208-bound KOR shows similar patters to the **KB series** compounds, with the unique bias path on TM4. Interestingly the signal transduction on TM4 does not originate at the top of TM4 but goes from the ECL2 through TM3 then into the switch residue W183$^{4.50}$ finaly down through TM4 and into the ICL2.

Analytical Characterization of Active Compounds KB01-KB08

HPLC purity

HPLC traces of final probes measured on an Agilent 1260 series HPLC system employing a DAD detector (300, 254 and 200 nm) and an ELSD detector equipped with an Agilent Technologies 6120 Quadrupole LC/MS in electrospray positive and negative ionization modes (ESI-MS). A Thermo Accuore RP-MS (30 $\times$ 2.1 mm, 2.6 µm) column was used with a flow rate 0.8 mL/min in combination with the following separation conditions: 0.1% formic acid in water (solvent A); 0.1% formic acid in ACN (solvent B); System 5% B for 0.2 min, from 5 to 95% B in 0.9 min, 95% B for 1.4 min (stop point at 2.5 min or 8 min). Data analysis was performed with ChemStation software (version 2.156.0.0). The purity of all test compounds was determined to be $> 95\%$.

Compound **KB01** (254 nm trace)

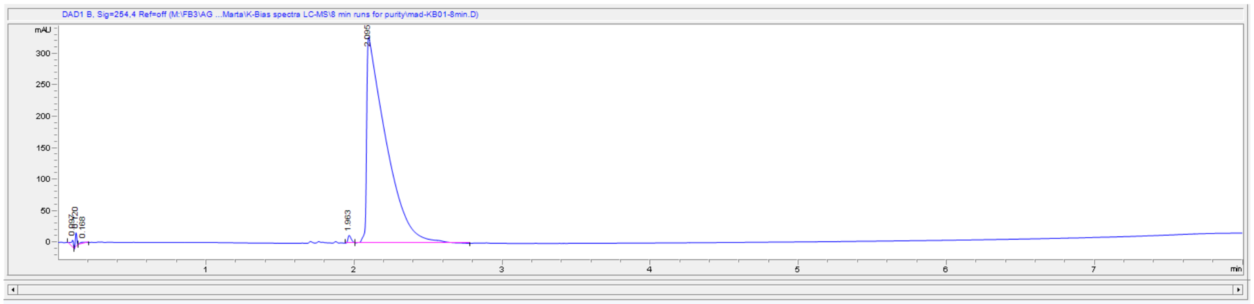

#	Time	Type	Area	Height	Width	Area%	Symmetry
1	9.657E-2	BB	12	11.3	0.0154	0.393	1.843
2	0.12	BB	15.8	22.7	0.0116	0.518	0.991
3	0.168	BB	6.2	2	0.0468	0.202	1.308
4	1.963	BB	19	12.2	0.0237	0.625	0.583
5	2.095	BB	2986.3	328.5	0.1166	98.261	0.11

Compound **KB01** (ELSD trace)

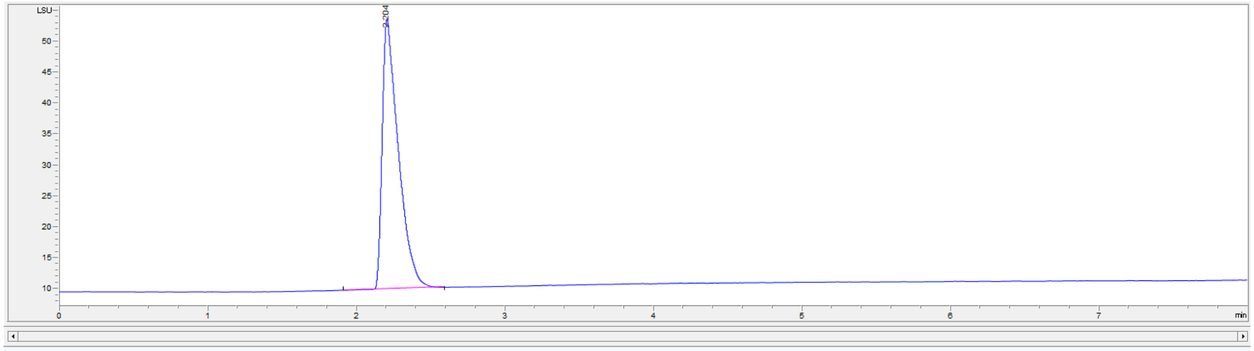

#	Time	Type	Area	Height	Width	Area%	Symmetry
1	2.204	BB	328.2	43.5	0.102	100.000	0.379

Mass spectrum of the peak eluting at 2.095 min

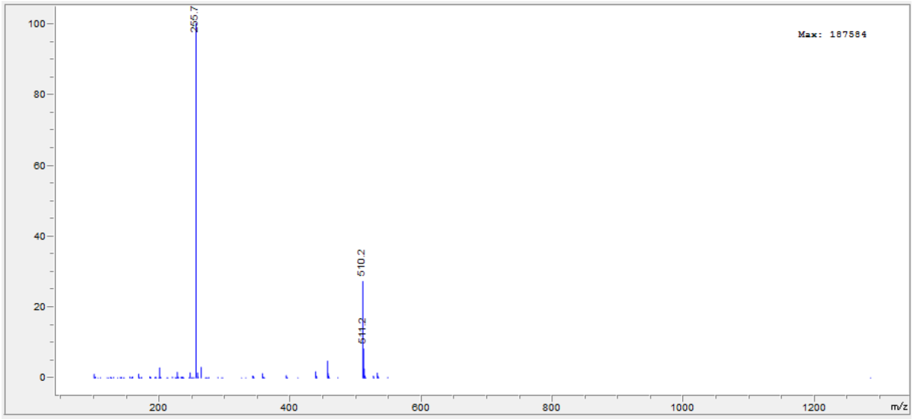

Compound **KB02** (254 nm trace)

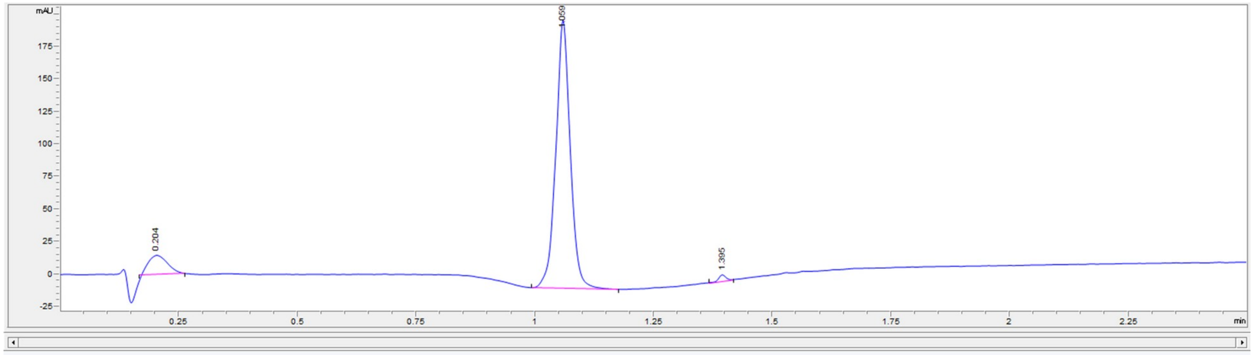

#	Time	Type	Area	Height	Width	Area%	Symmetry
1	0.204	MM	11.2	6.8	0.0275	2.457	1.267
2	1.059	BB	436.4	206	0.0312	95.484	0.941
3	1.395	MM	9.4	5.8	0.0268	2.059	0.805

Compound **KB02** (ELSD trace)

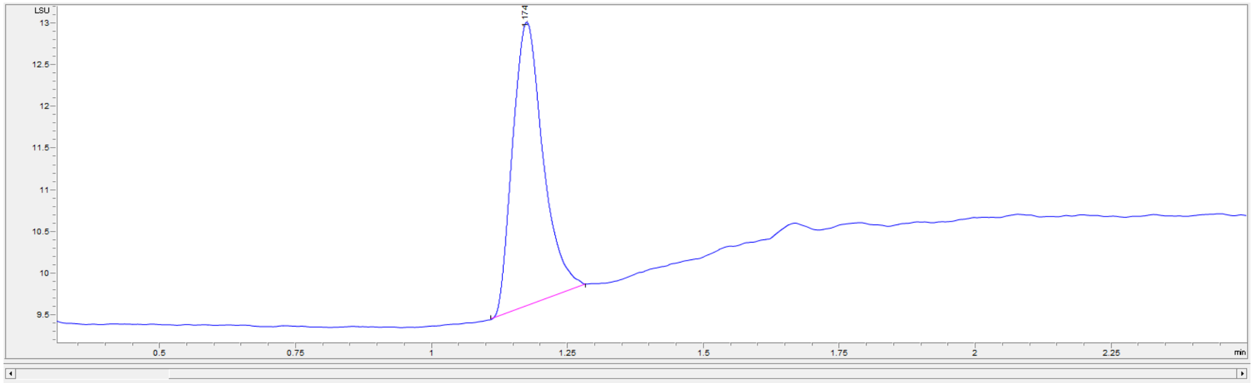

#	Time	Type	Area	Height	Width	Area%	Symmetry
1	1.174	BB	13.3	3.4	0.061	100.000	0.795

Mass spectrum of the peak at 1.059 min

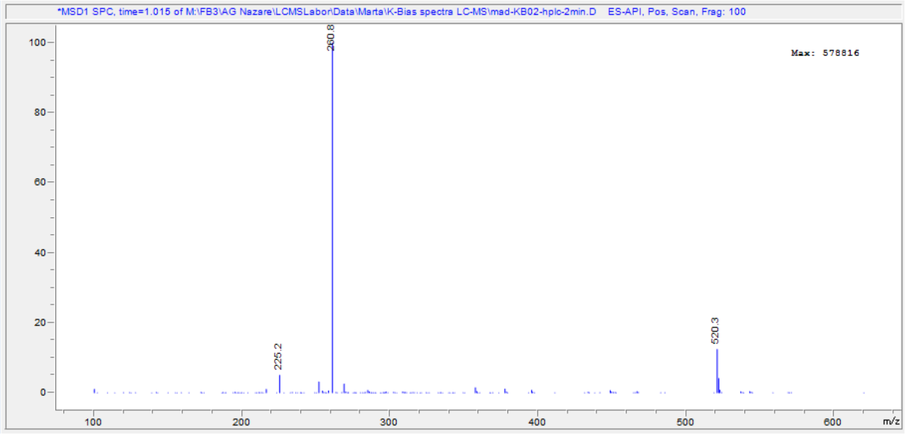

Compound **KB03** (254 nm trace)

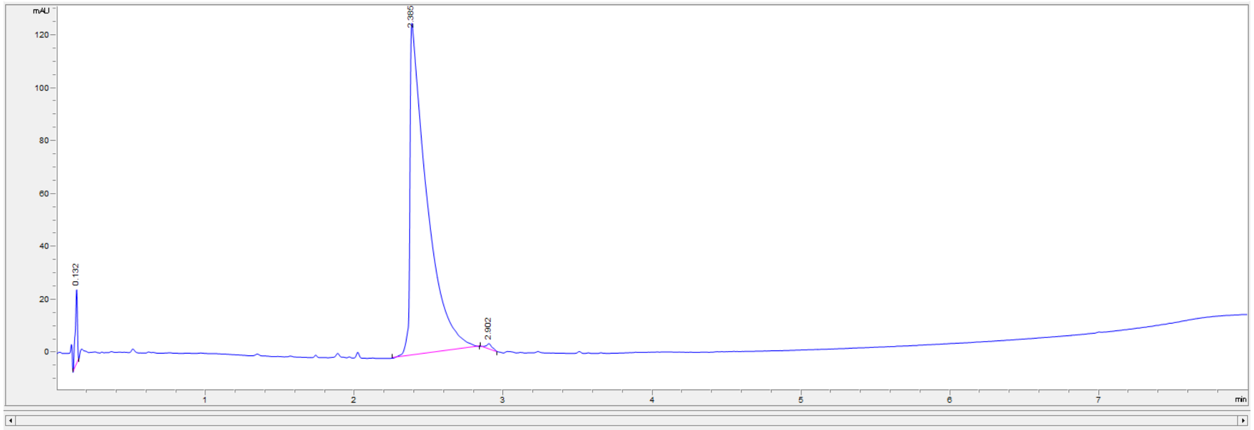

#	Time	Type	Area	Height	Width	Area%	Symmetry
1	0.132	BB	28.5	28.3	0.0148	2.793	1.786
2	2.385	BB	987.5	125.6	0.1013	96.642	0.132
3	2.902	BB	5.8	2.1	0.0387	0.565	0.868

Compound **KB03** (ELSD trace)

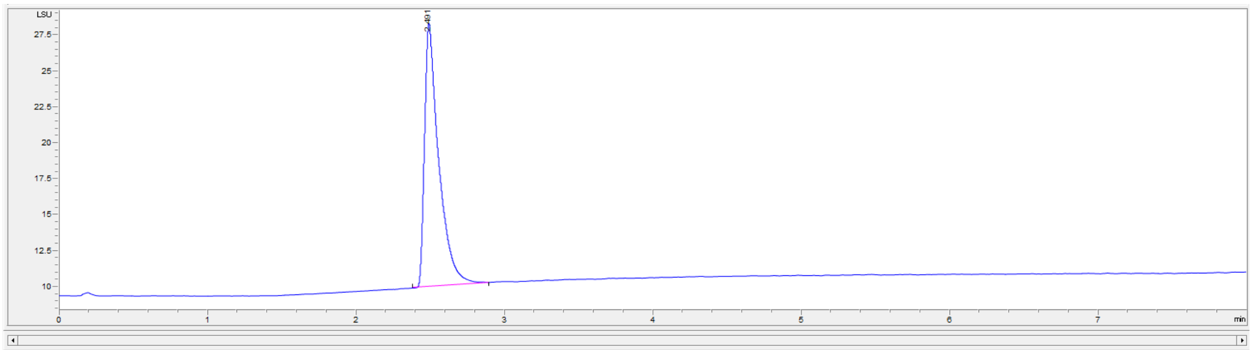

#	Time	Type	Area	Height	Width	Area%	Symmetry
1	2.491	BB	121.4	18.3	0.088	100.000	0.416

Mass spectrum of the peak at 2.385 min

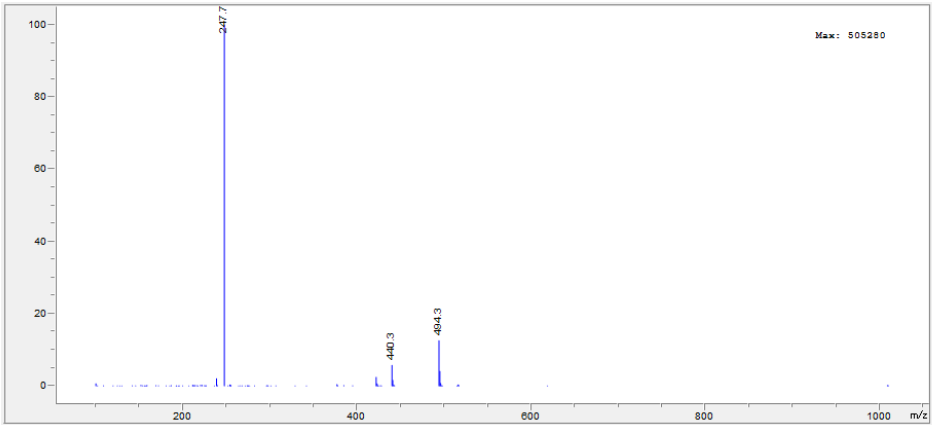

Compound **KB04** (254 nm trace)

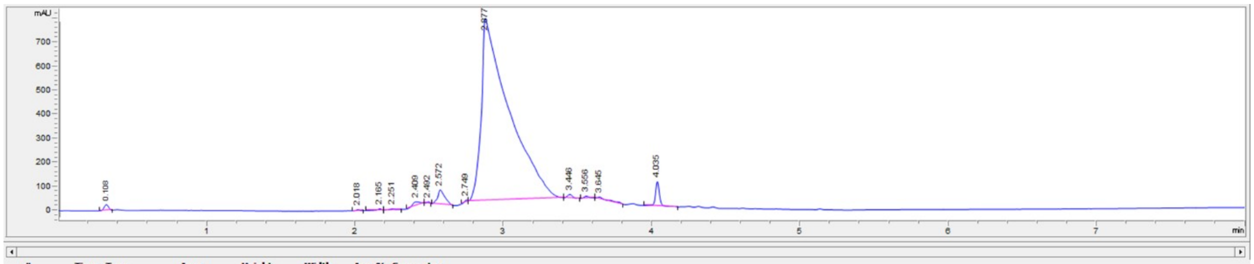

#	Time	Type	Area	Height	Width	Area%	Symmetry
1	0.108	MM	9.3	19.9	7.803E-3	0.091	0
2	2.018	BB	6.4	4.3	0.0234	0.062	0.973
3	2.165	BB	9.1	5.7	0.0243	0.089	1.645
4	2.251	BB	8.7	3	0.0404	0.084	0.8
5	2.409	BB	59.7	17	0.0504	0.580	0.957
6	2.492	BB	8.1	6.4	0.0202	0.079	0.854
7	2.572	BB	207.8	60.2	0.0487	2.018	0.571
8	2.749	MM	12.9	9.1	0.0237	0.125	2.604
9	2.876	MM	9793	757.5	0.2155	95.108	0.183
10	3.446	MM	6.6	5.7	0.0193	0.064	1.241
11	3.556	BB	19	8.2	0.0357	0.185	0.758
12	3.645	BB	39.9	7.7	0.0683	0.388	0.178
13	4.035	MM	116.1	83.1	0.0233	1.128	0.895

Compound **KB04** (ELSD trace)

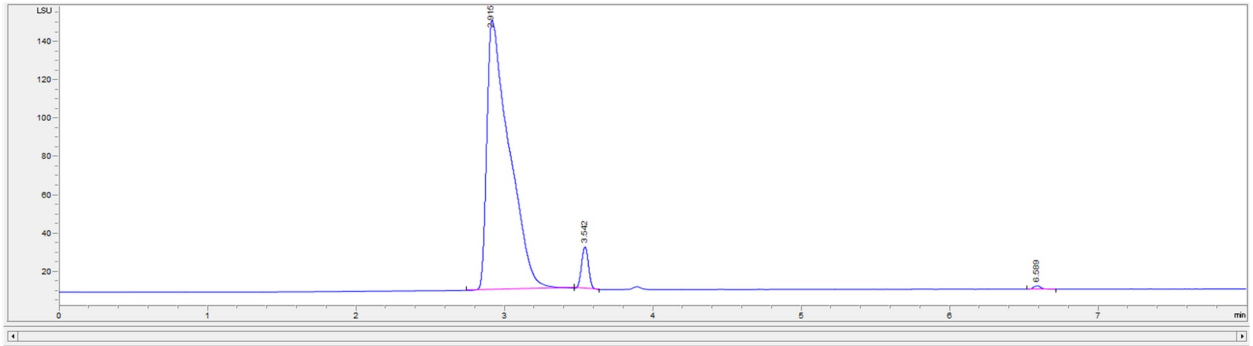

#	Time	Type	Area	Height	Width	Area%	Symmetry
1	2.915	BB	1474.6	140.8	0.1347	95.046	0.279
2	3.542	BB	71.1	21.9	0.0519	4.580	1.004
3	6.589	BB	5.8	1.7	0.0545	0.374	1.034

Mass spectrum of the peak at 2.876 min

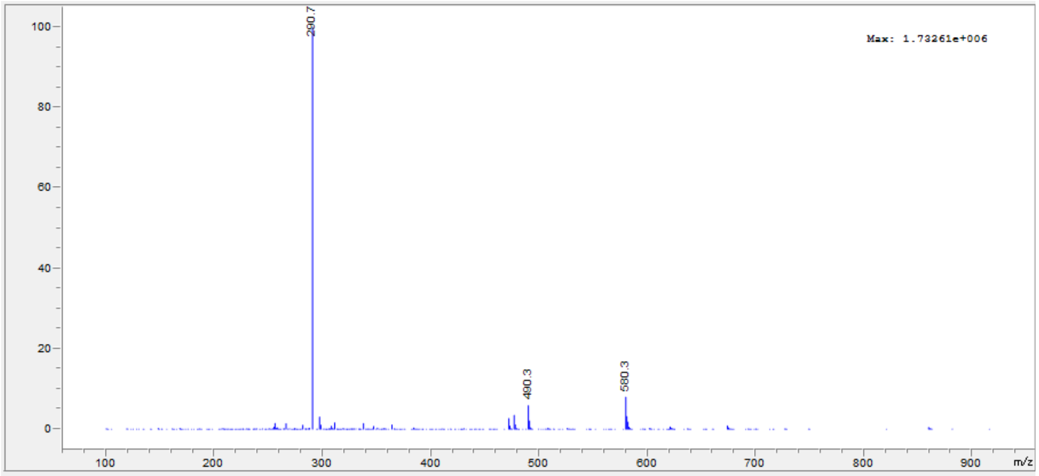

Compound **KB05** (254 nm trace)

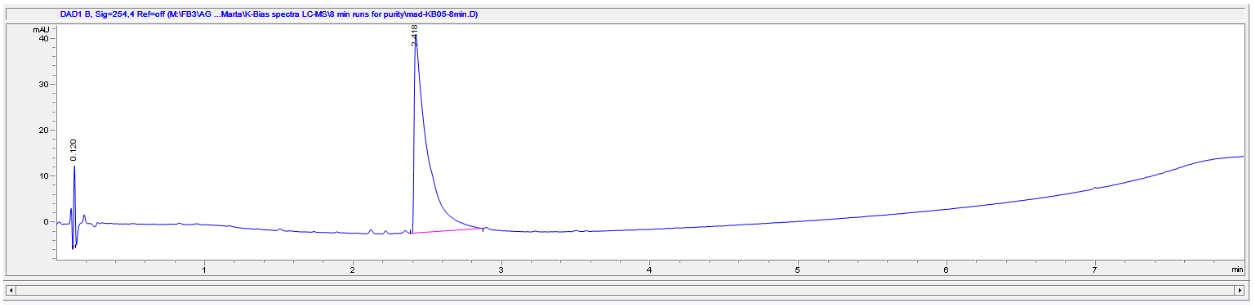

#	Time	Type	Area	Height	Width	Area%	Symmetry
1	0.12	BB	12	17.9	0.0108	4.417	0.948
2	2.418	BB	259.1	42.9	0.0785	95.583	0.13

Compound **KB05** (ELSD trace)

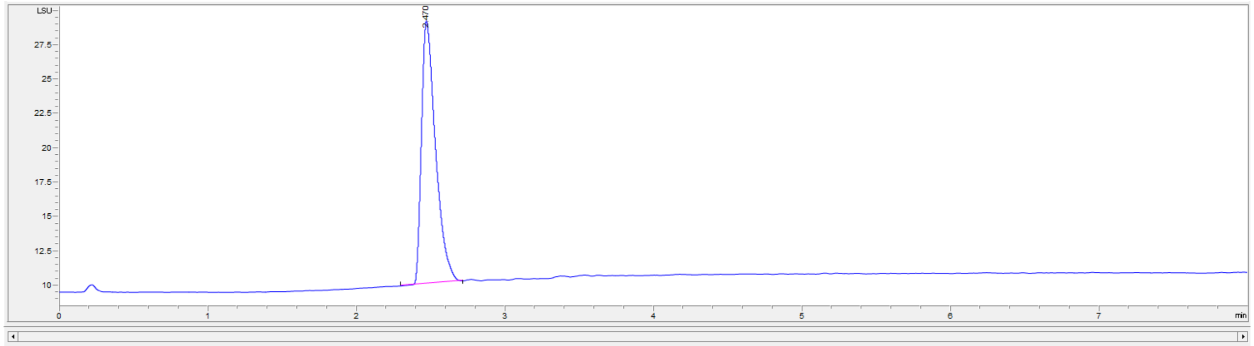

#	Time	Type	Area	Height	Width	Area%	Symmetry
1	2.47	BB	126.5	19.2	0.0933	100.000	0.499

Mass spectrum of the peak at 2.418 min

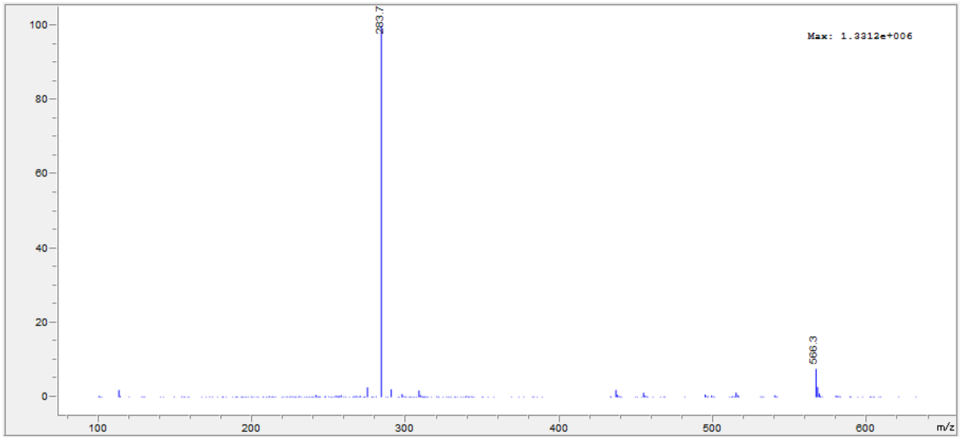

Compound **KB06** (254 nm trace)

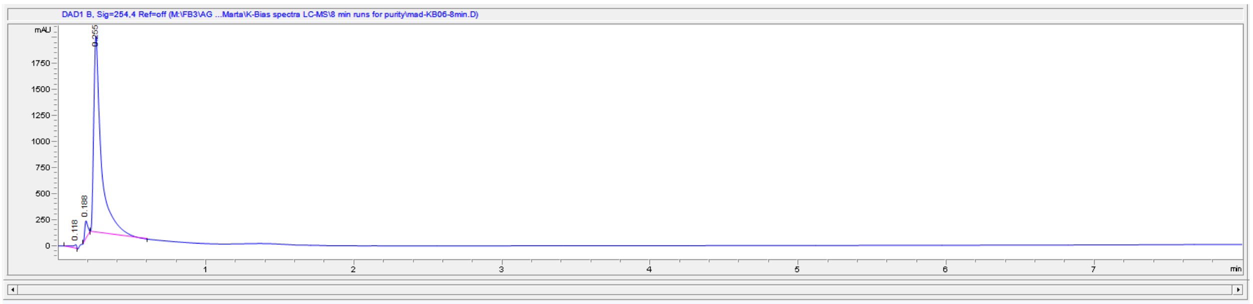

#	Time	Type	Area	Height	Width	Area%	Symmetry
1	0.118	BB	79.3	37.8	0.028	1.133	4.123
2	0.188	BB	242.3	172.9	0.0214	3.463	0.749
3	0.255	BB	6676.6	1884.4	0.0493	95.404	0.368

Compound **KB06** (ELSD trace)

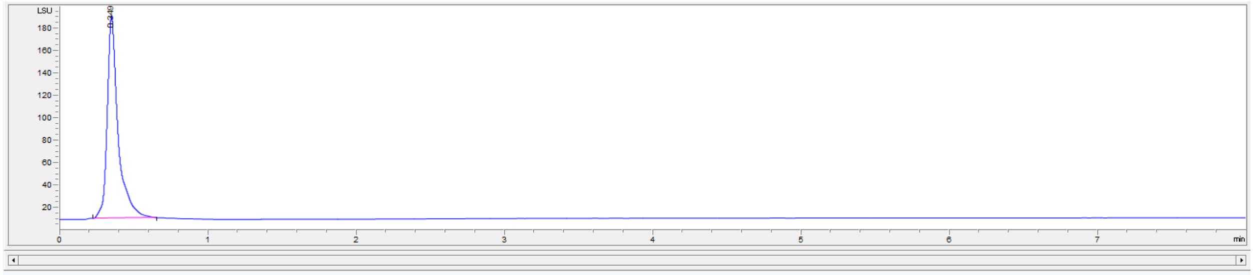

#	Time	Type	Area	Height	Width	Area%	Symmetry
1	0.349	BB	914.9	179.7	0.0739	100.000	0.594

Mass spectrum of the peak at 0.255 min

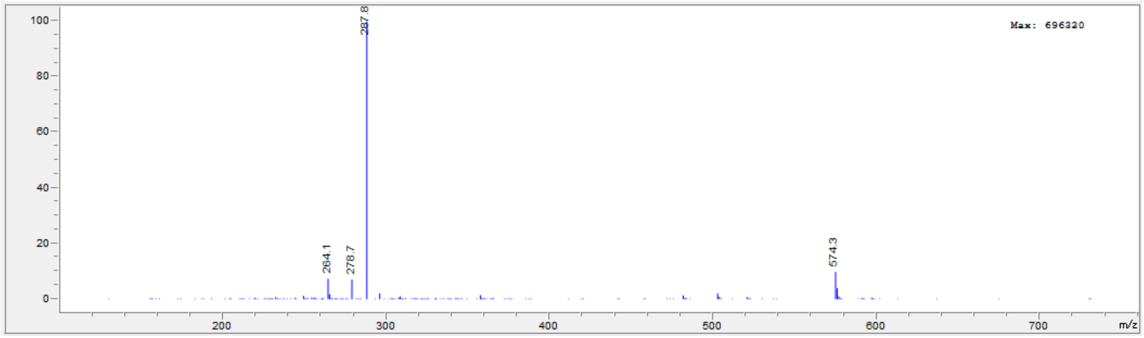

Compound **KB07** (254 nm trace)

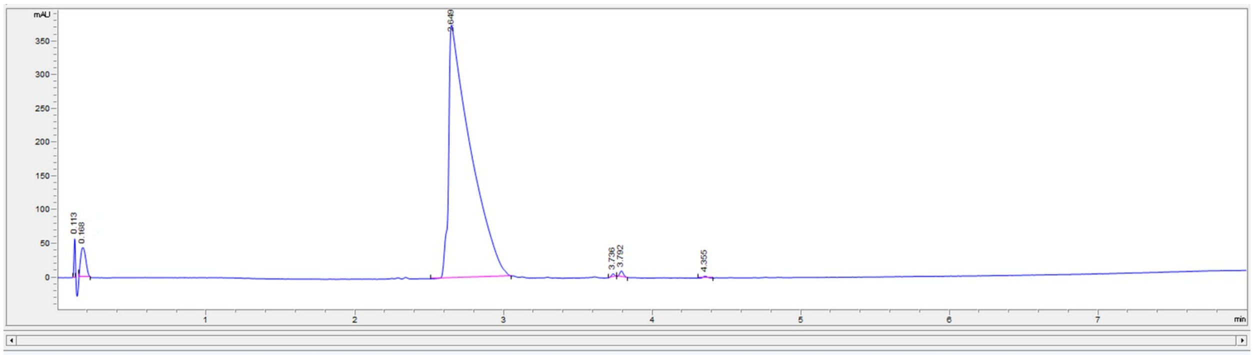

#	Time	Type	Area	Height	Width	Area%	Symmetry
1	0.113	MM	35.8	58.5	0.0102	0.893	1.159
2	0.168	MM	108.5	44.8	0.0404	2.701	0.703
3	2.649	BB	3842.9	376.4	0.1311	95.707	0.152
4	3.736	BB	7.1	4.5	0.0254	0.177	1.037
5	3.792	BB	15.2	8.8	0.0266	0.379	0.787
6	4.355	BB	5.8	2.8	0.0316	0.143	0.697

Compound **KB07** (ELSD trace)

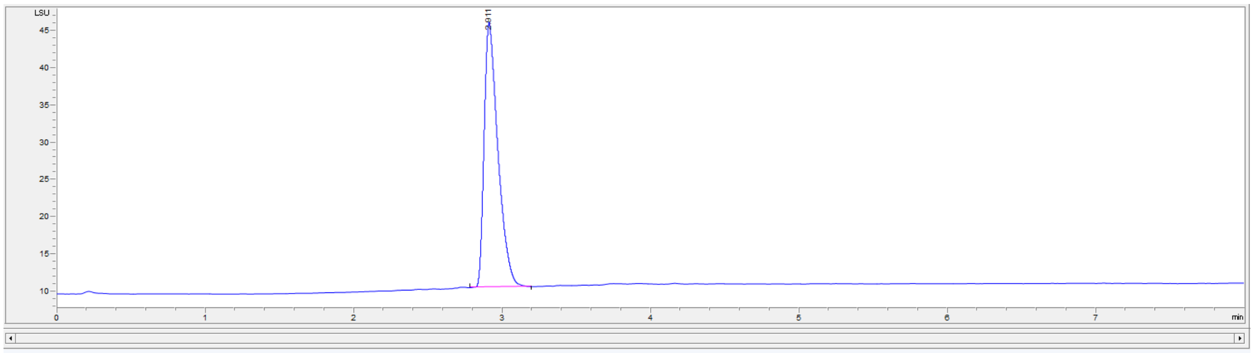

#	Time	Type	Area	Height	Width	Area%	Symmetry
1	2.911	BB	229.3	35.6	0.0922	100.000	0.514

Mass spectrum of the peak at 2.649 min

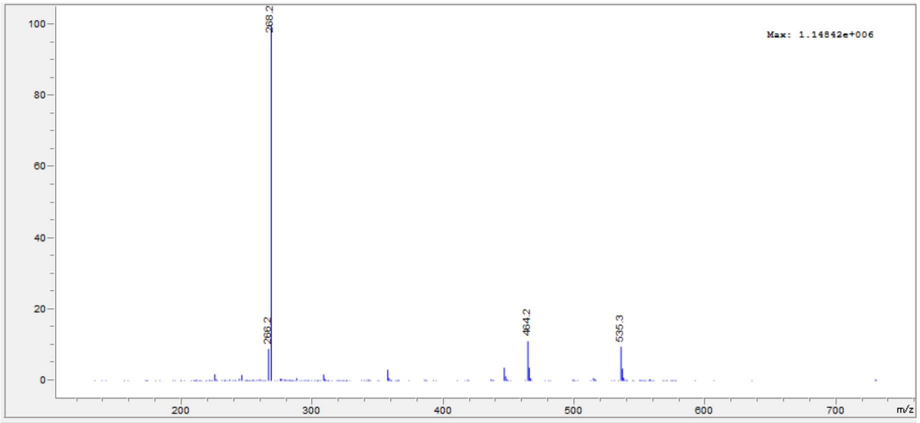

Compound **KB08** (254 nm trace)

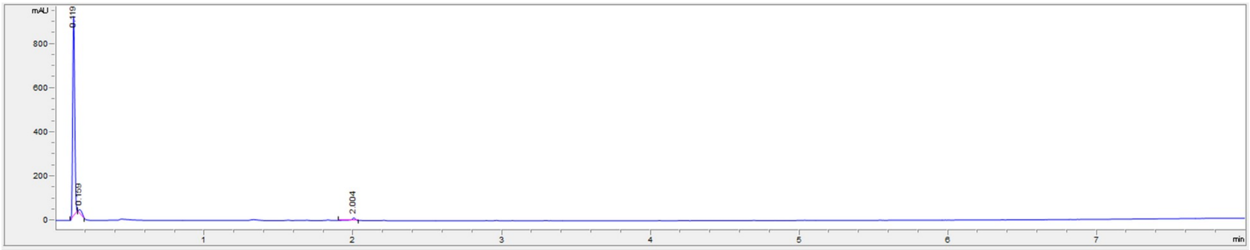

#	Time	Type	Area	Height	Width	Area%	Symmetry
1	0.119	MM	904.5	923.9	0.0163	92.954	0.798
2	0.159	MM	54.5	33	0.0276	5.606	0.722
3	2.004	MM	14	10.9	0.0215	1.440	1.241

Compound **KB08** (ELSD trace)

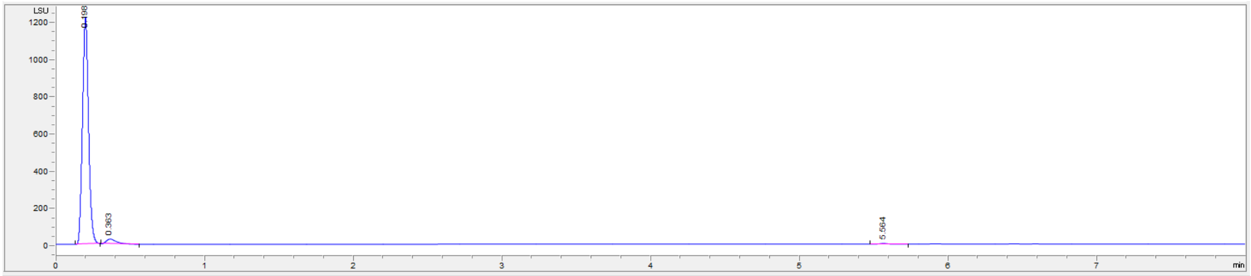

#	Time	Type	Area	Height	Width	Area%	Symmetry
1	0.198	BB	3438.6	1216.2	0.044	96.195	0.938
2	0.363	BB	125.1	26.6	0.0699	3.499	0.555
3	5.564	BB	11	3.3	0.0542	0.307	0.958

Mass spectrum of the peak at 0.119 min

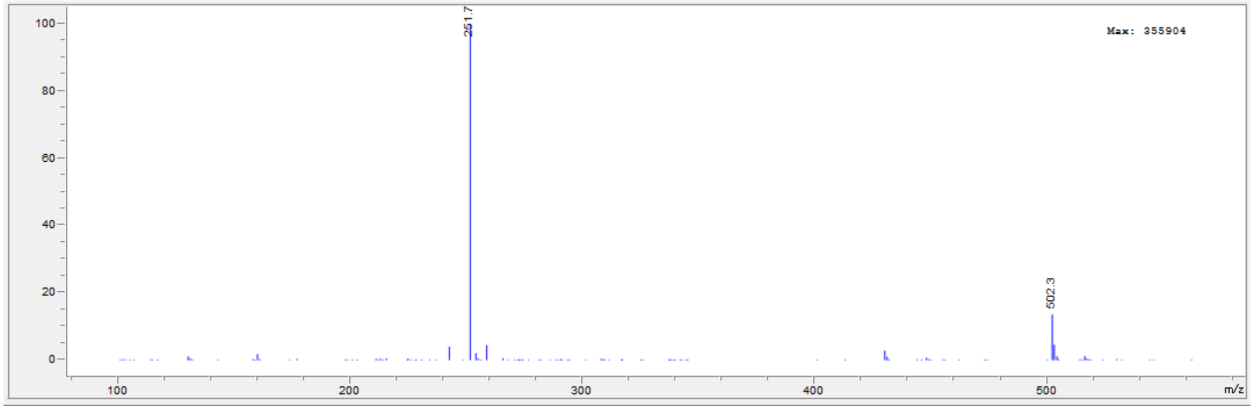

High-Resolution Mass-Spectrometry (HRMS)

Compound **KB01**

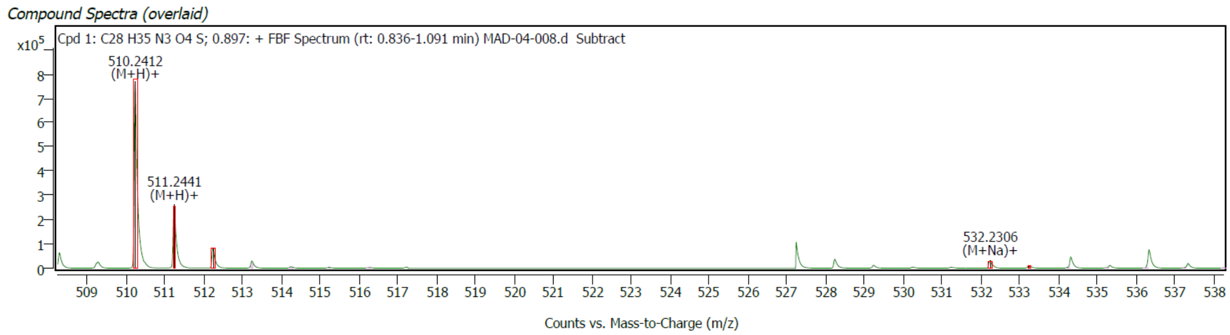

Spectrum Peaks

m/z	m/z (Calc)	Diff (ppm)	Abund	Height %	Height % (Calc)	Ion Species	Z
510.2412	510.2421	-1.83	766560	100.00	100.00	(M+H)+	1
511.2441	511.2452	-2.19	261286	34.09	32.74	(M+H)+	1
512.2431	512.2437	-1.15	80770	10.54	10.49	(M+H)+	1
532.2306	532.2240	12.39	30382	100.00	100.00	(M+Na)+	1
533.2304	533.2271	6.19	11522	37.92	32.72	(M+Na)+	1

Compound **KB02**

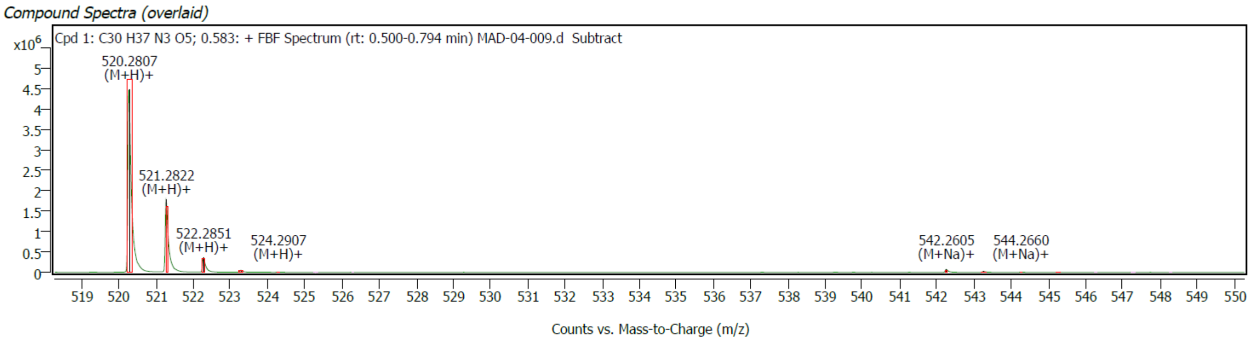

Spectrum Peaks

m/z	m/z (Calc)	Diff (ppm)	Abund	Height %	Height % (Calc)	Ion Species	Z
520.2807	520.2806	0.16	4476886	100.00	100.00	(M+H)+	1
521.2822	521.2838	-3.08	1776648	39.68	34.17	(M+H)+	1
522.2851	522.2867	-2.96	361188	8.07	6.69	(M+H)+	1
523.2879	523.2894	-2.86	53041	1.18	0.96	(M+H)+	1
524.2907	524.2921	-2.67	6669	0.15	0.11	(M+H)+	1
542.2605	542.2625	-3.84	71876	100.00	100.00	(M+Na)+	1
543.2634	543.2657	-4.21	24789	34.49	34.16	(M+Na)+	1
544.2660	544.2686	-4.69	5161	7.18	6.68	(M+Na)+	1
545.2699	545.2713	-2.69	1000	1.39	0.96	(M+Na)+	1

Compound **KB03**

Compound Spectra (overlaid)

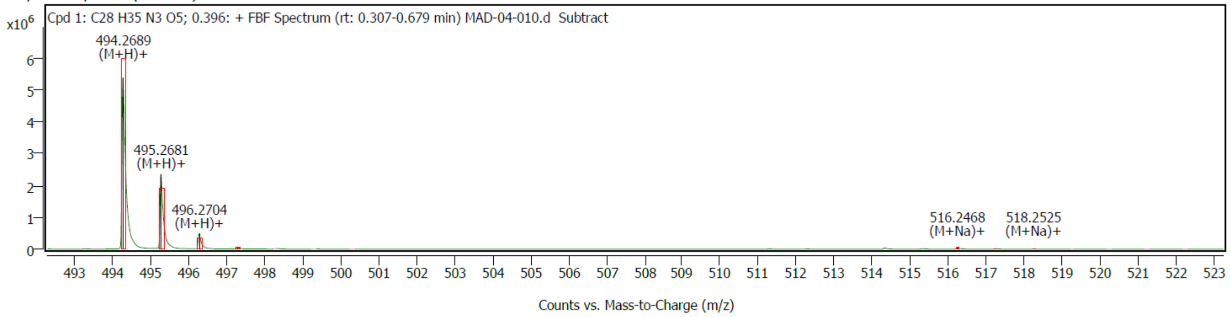

Spectrum Peaks

m/z	m/z (Calc)	Diff (ppm)	Abund	Height %	Height % (Calc)	Ion Species	Z
494.2689	494.2649	7.99	5380334	100.00	100.00	(M+H)+	1
495.2681	495.2681	-0.08	2336337	43.42	31.98	(M+H)+	1
496.2704	496.2709	-1.05	477014	8.87	5.98	(M+H)+	1
497.2735	497.2736	-0.23	67293	1.25	0.82	(M+H)+	1
516.2468	516.2469	-0.10	38224	100.00	100.00	(M+Na)+	1
517.2493	517.2501	-1.51	12142	31.76	31.97	(M+Na)+	1
518.2525	518.2529	-0.69	2599	6.80	5.97	(M+Na)+	1

Compound **KB04**

Compound Spectra (overlaid)

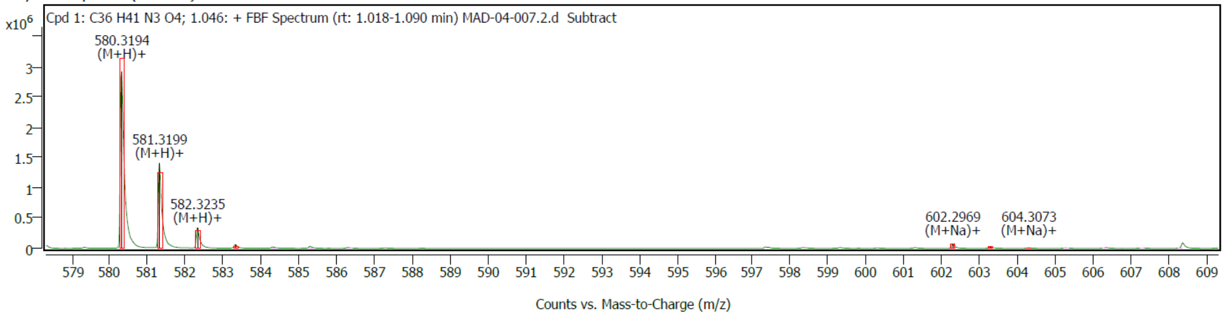

Spectrum Peaks

m/z	m/z (Calc)	Diff (ppm)	Abund	Height %	Height % (Calc)	Ion Species	Z
580.3194	580.3170	4.11	2910031	100.00	100.00	(M+H)+	1
581.3199	581.3202	-0.58	1397542	48.02	40.67	(M+H)+	1
582.3235	582.3232	0.50	336418	11.56	8.88	(M+H)+	1
583.3243	583.3261	-3.01	62008	2.13	1.37	(M+H)+	1
602.2969	602.2989	-3.29	66962	100.00	100.00	(M+Na)+	1
603.2997	603.3021	-4.06	28570	42.67	40.66	(M+Na)+	1
604.3073	604.3052	3.47	8448	12.62	8.87	(M+Na)+	1

Compound **KB05**

Compound Spectra (overlaid)

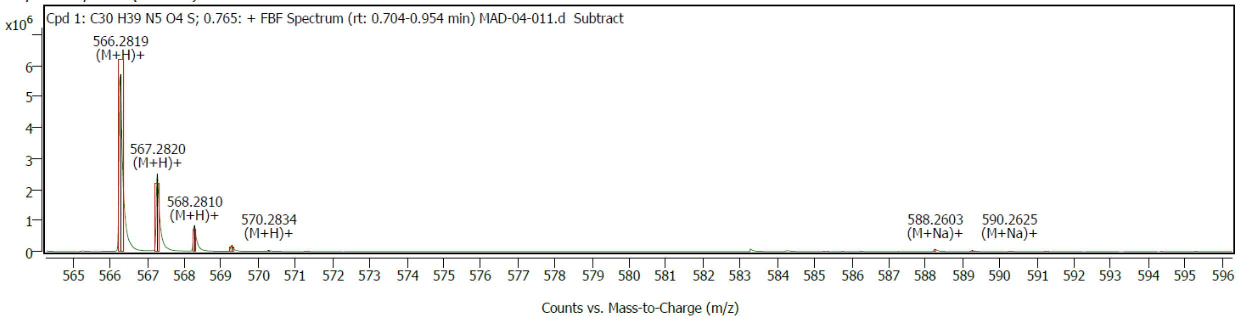

Spectrum Peaks

m/z	m/z (Calc)	Diff (ppm)	Abund	Height %	Height % (Calc)	Ion Species	Z
566.2819	566.2796	4.18	5691455	100.00	100.00	(M+H)+	1
567.2820	567.2825	-0.87	2506240	44.04	35.68	(M+H)+	1
568.2810	568.2814	-0.76	834083	14.65	11.48	(M+H)+	1
569.2822	569.2821	0.11	184681	3.24	2.55	(M+H)+	1
570.2834	570.2835	-0.19	31287	0.55	0.42	(M+H)+	1
571.2843	571.2852	-1.60	4969	0.09	0.06	(M+H)+	1
588.2603	588.2615	-2.08	70500	100.00	100.00	(M+Na)+	1
589.2632	589.2645	-2.22	25007	35.47	35.66	(M+Na)+	1
590.2625	590.2634	-1.44	8086	11.47	11.47	(M+Na)+	1
591.2617	591.2641	-3.93	2045	2.90	2.55	(M+Na)+	1

Compound **KB06**

Compound Spectra (overlaid)

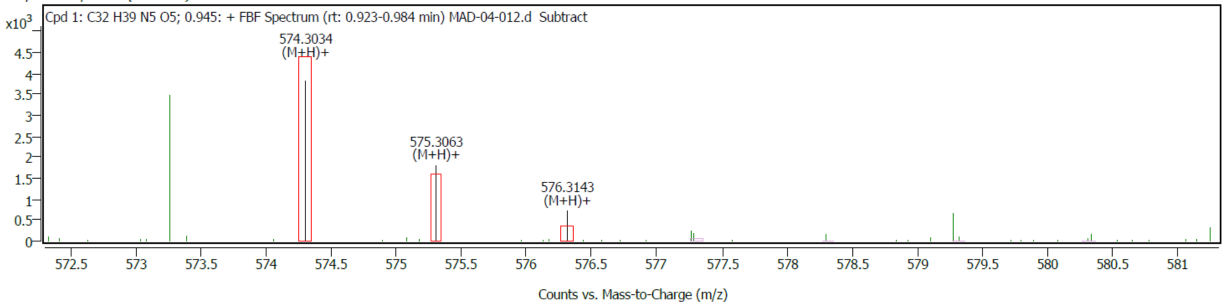

Spectrum Peaks

m/z	m/z (Calc)	Diff (ppm)	Abund	Height %	Height % (Calc)	Ion Species	Z
574.3034	574.3024	1.76	3823	100.00	100.00	(M+H)+	1
575.3063	575.3055	1.38	1811	47.37	37.09	(M+H)+	1
576.3143	576.3083	10.39	714	18.68	7.71	(M+H)+	1

Compound **KB07**

Compound Spectra (overlaid)

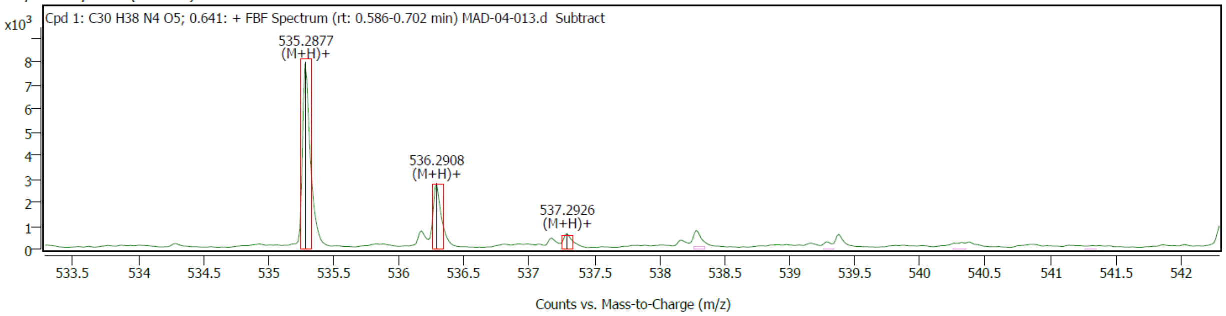

Spectrum Peaks

m/z	m/z (Calc)	Diff (ppm)	Abund	Height %	Height % (Calc)	Ion Species	Z
535.2877	535.2915	-7.14	7954	100.00	100.00	(M+H)+	1
536.2908	536.2946	-7.18	2796	35.15	34.55	(M+H)+	1
537.2926	537.2974	-8.97	649	8.16	6.82	(M+H)+	1

Compound **KB08**

Compound Spectra (overlaid)

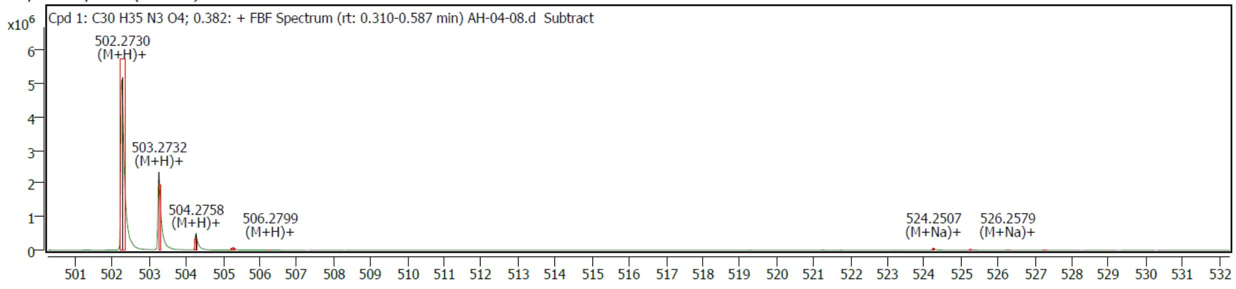

Spectrum Peaks

m/z	m/z (Calc)	Diff (ppm)	Abund	Height %	Height % (Calc)	Ion Species	Z
502.2730	502.2700	5.91	5172500	100.00	100.00	(M+H)+	1
503.2732	503.2732	-0.13	2348747	45.41	34.11	(M+H)+	1
504.2758	504.2761	-0.70	490504	9.48	6.46	(M+H)+	1
505.2789	505.2789	-0.04	68307	1.32	0.88	(M+H)+	1
506.2799	506.2816	-3.47	8124	0.16	0.10	(M+H)+	1
524.2507	524.2520	-2.40	49695	100.00	100.00	(M+Na)+	1
525.2542	525.2552	-1.75	17358	34.93	34.10	(M+Na)+	1
526.2579	526.2581	-0.34	3472	6.99	6.46	(M+Na)+	1
527.2600	527.2609	-1.69	718	1.45	0.88	(M+Na)+	1

NMR spectra (^{1}HNMR and ^{13}CNMR)

KB01 ^{1}H-NMR (MeOD, 300 MHz)

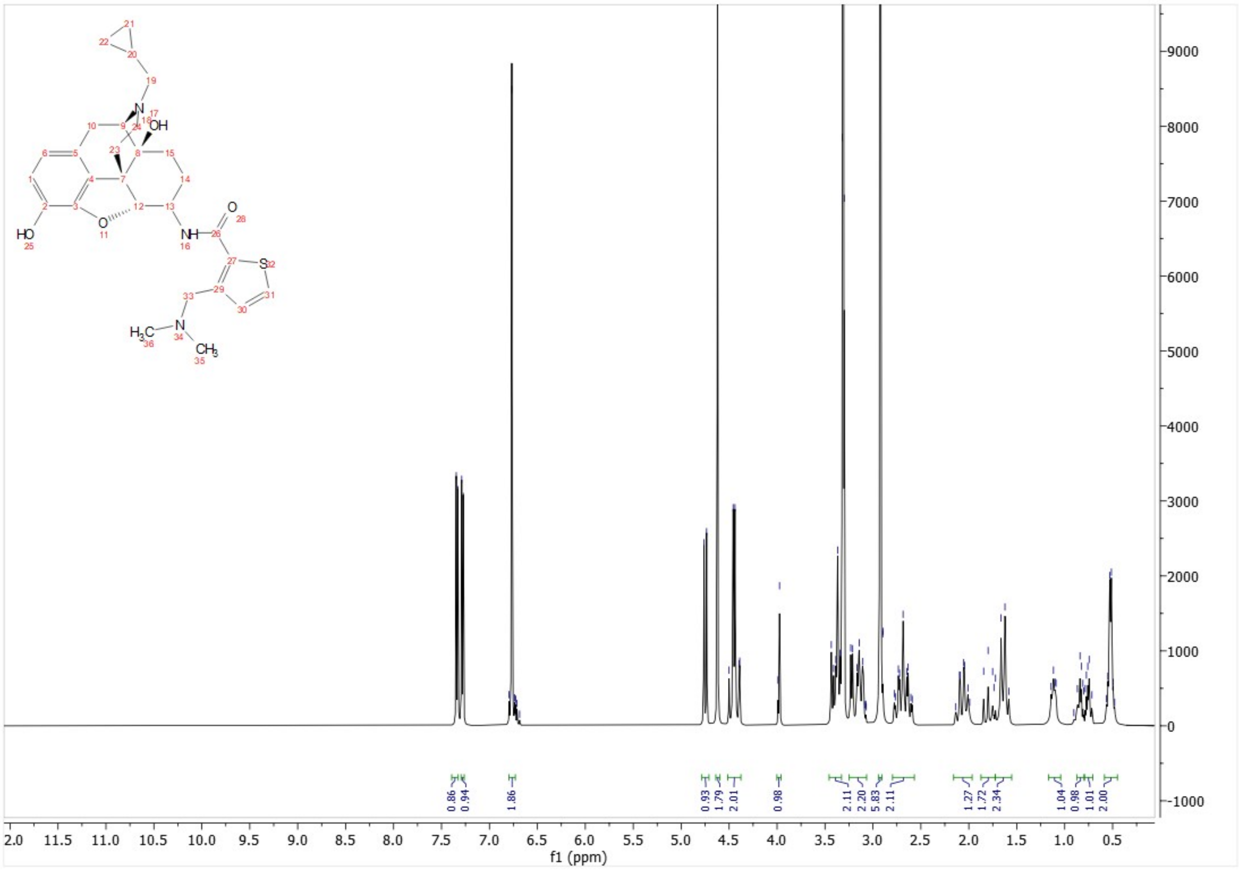

KB01 ^{13}C-NMR (MeOD, 600 MHz)

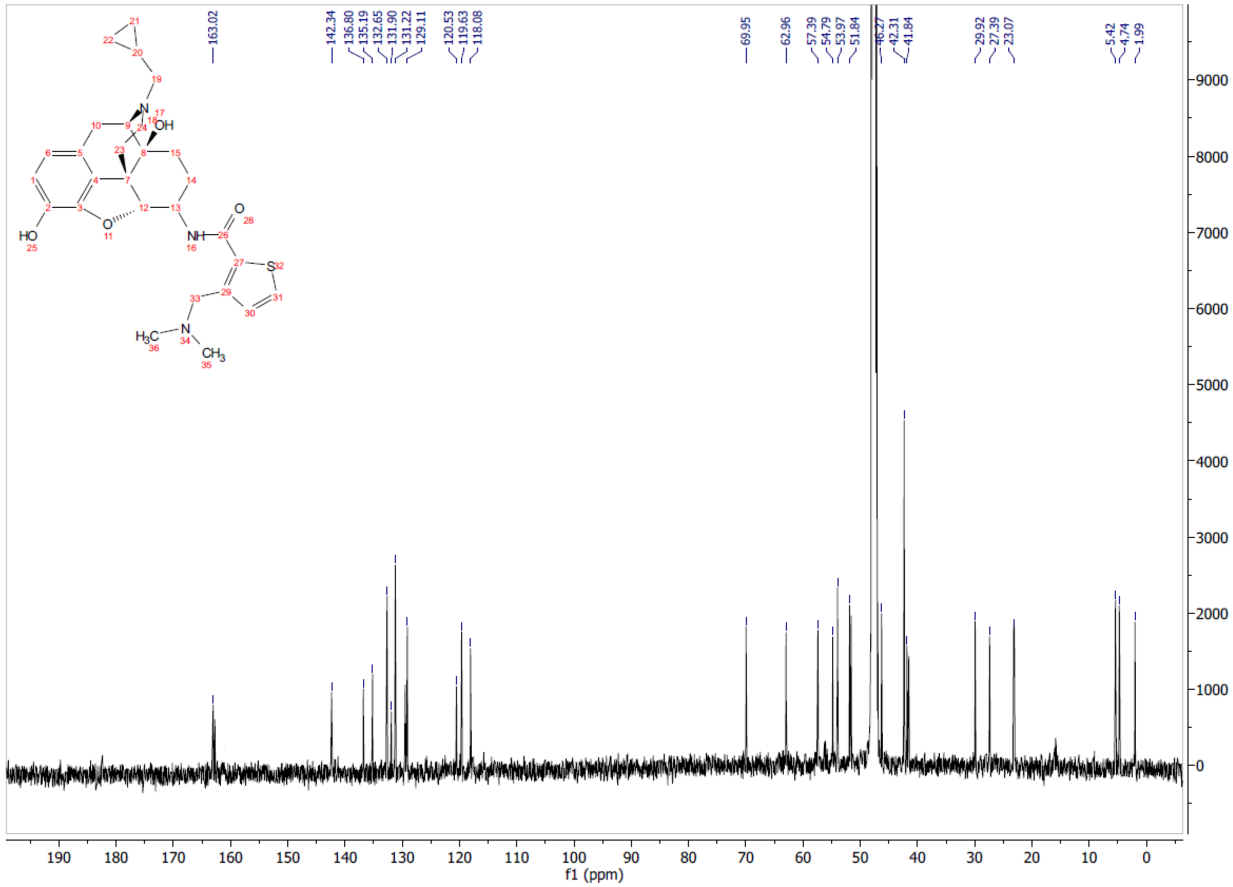

KB02 ^{1}H-NMR (MeOD, 300 MHz)

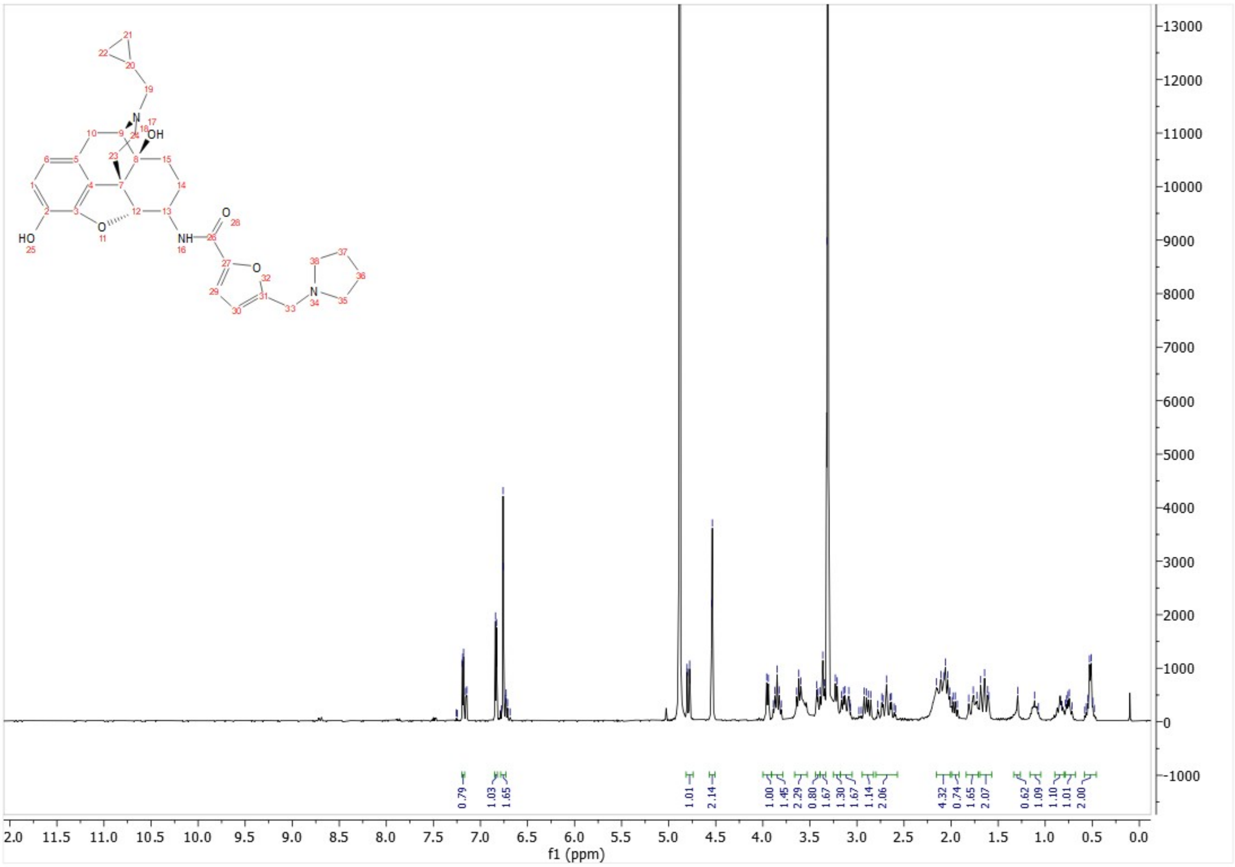

KB02 ^{13}C-NMR (MeOD, 600 MHz)

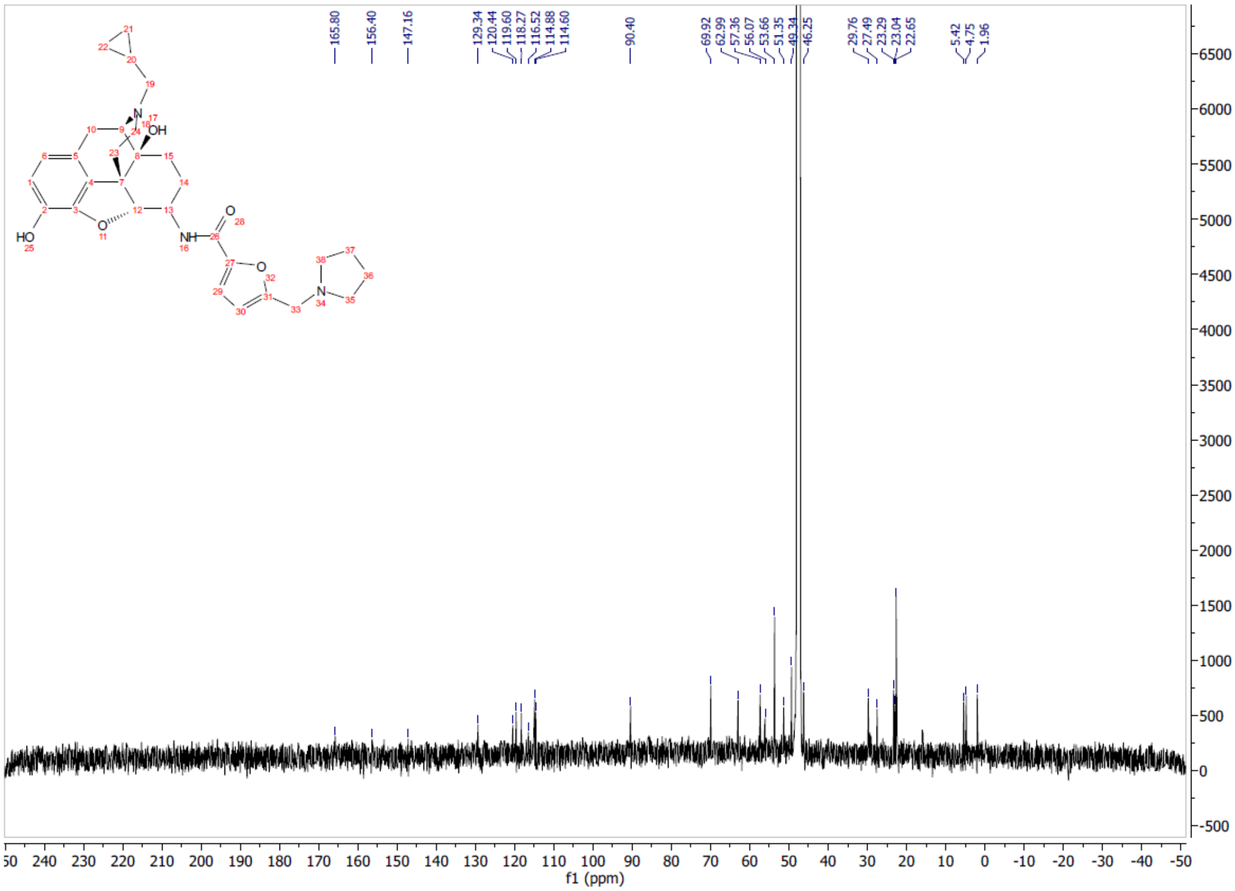

KB03 ^{1}H-NMR (MeOD, 300 MHz)

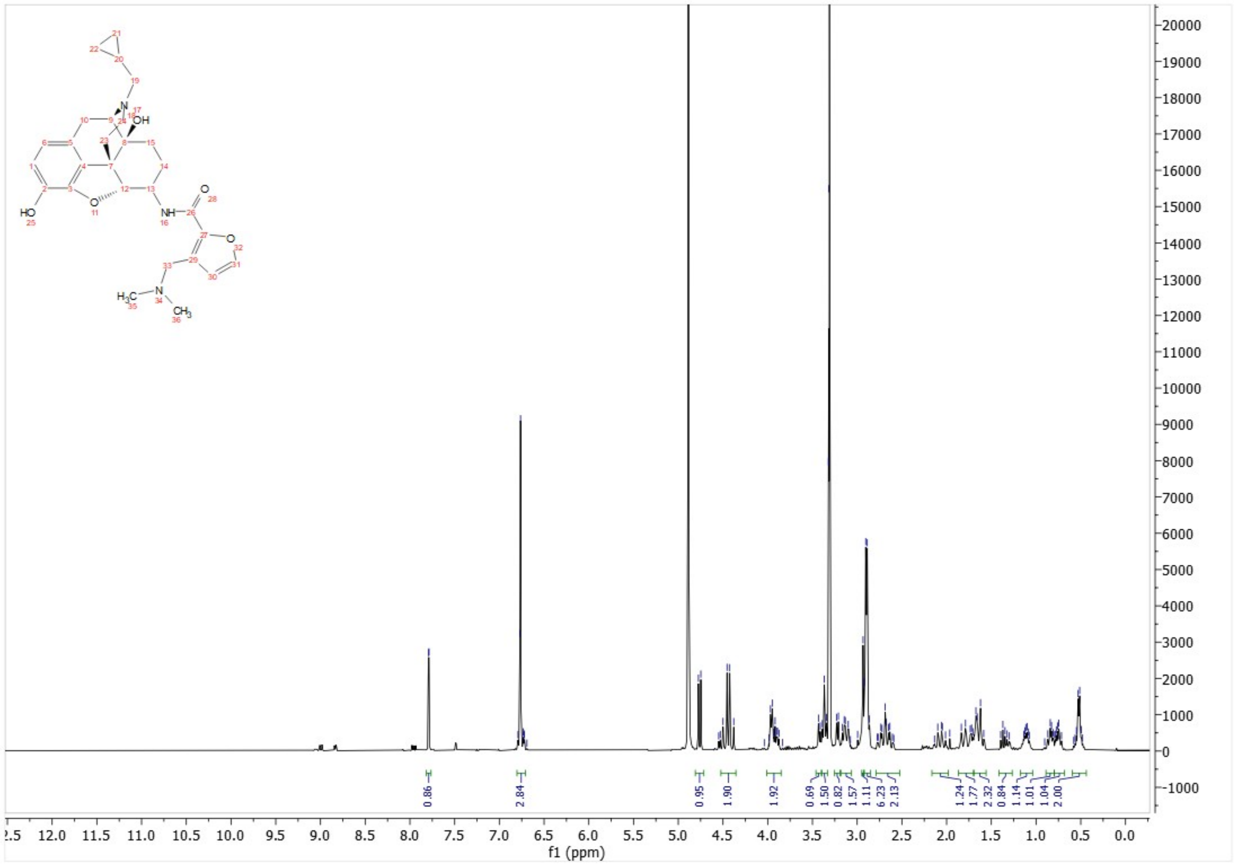

KB03 ^{13}C-NMR (MeOD, 600 MHz)

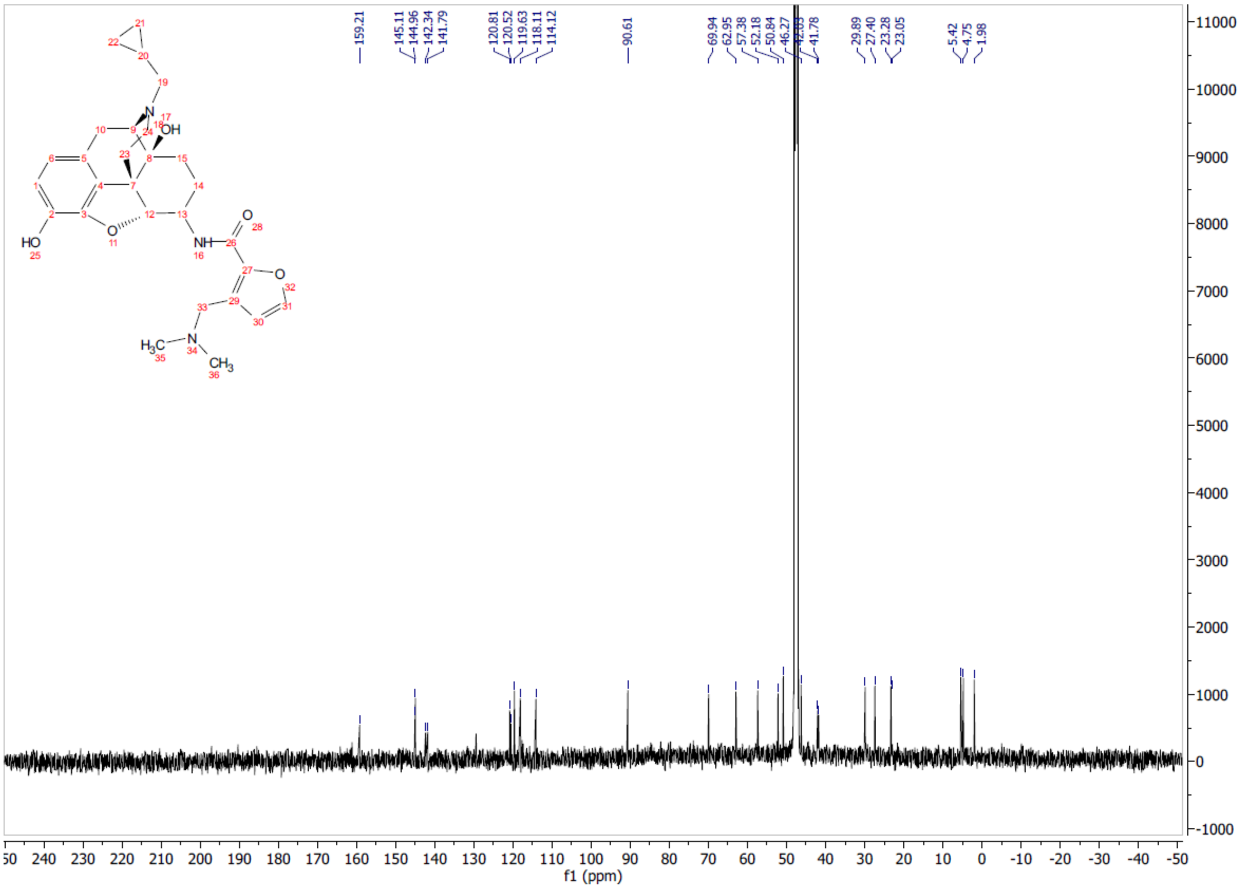

KB04 ^{1}H-NMR (MeOD:$CDCl_3$ 4:1 300 MHz)

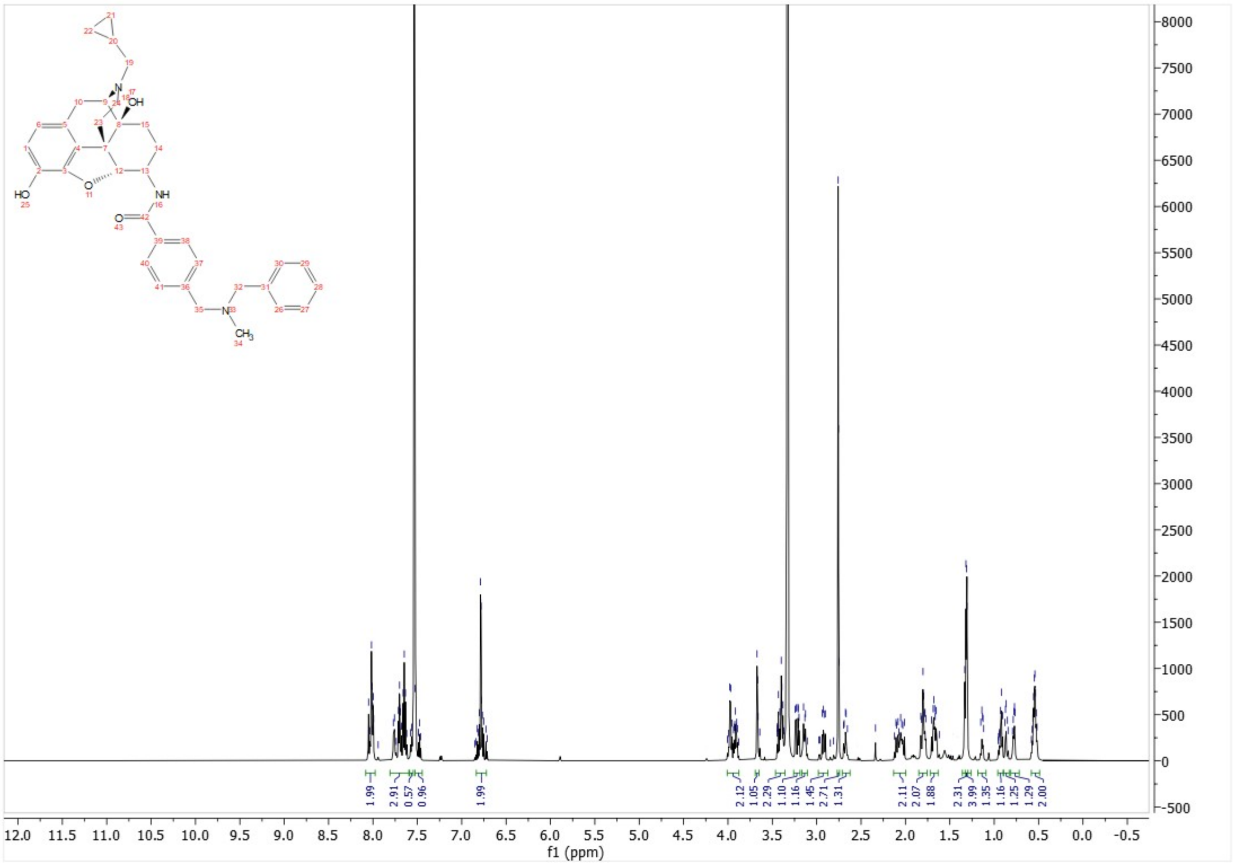

KB04 ^{13}C-NMR (MeOD, 600 MHz)

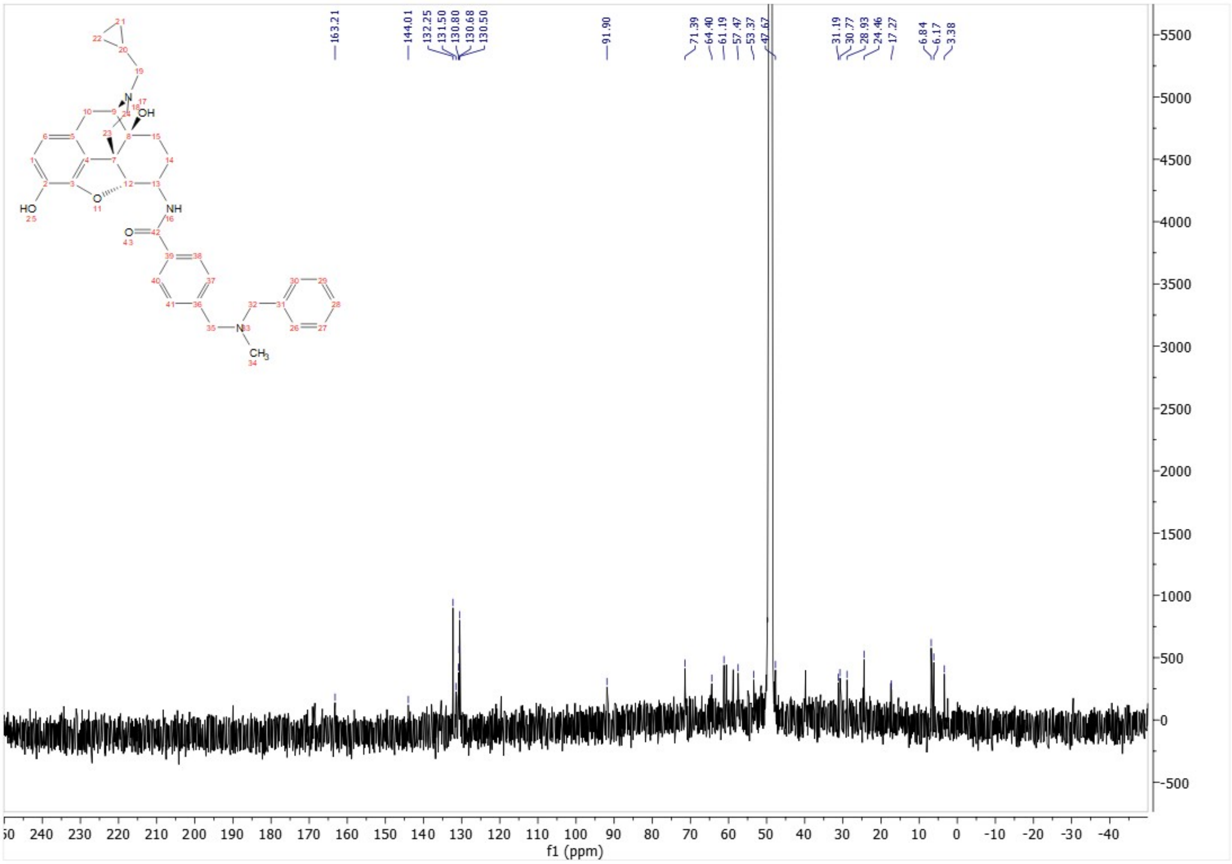

KB05 ^{1}H-NMR (MeOD, 600 MHz)

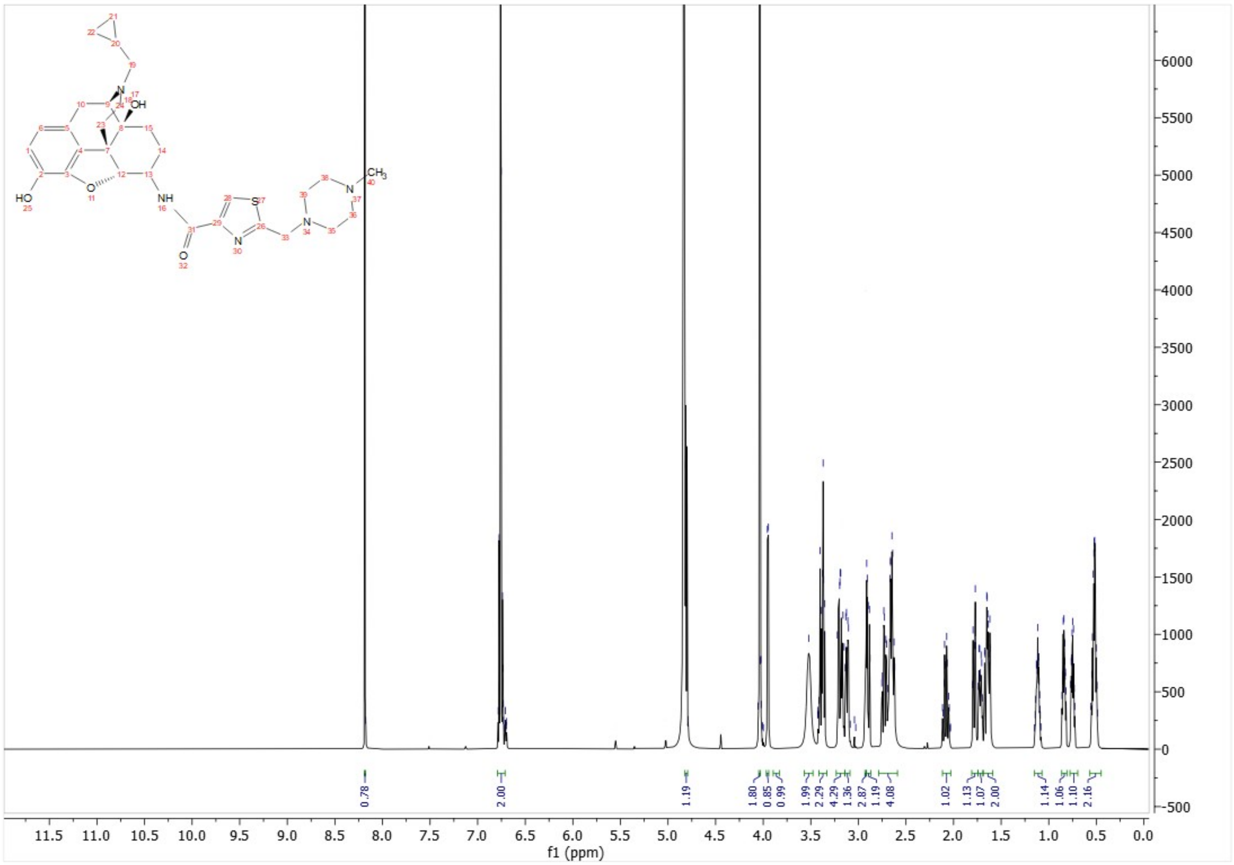

KB05 ^{13}C-NMR (MeOD, 600 MHz)

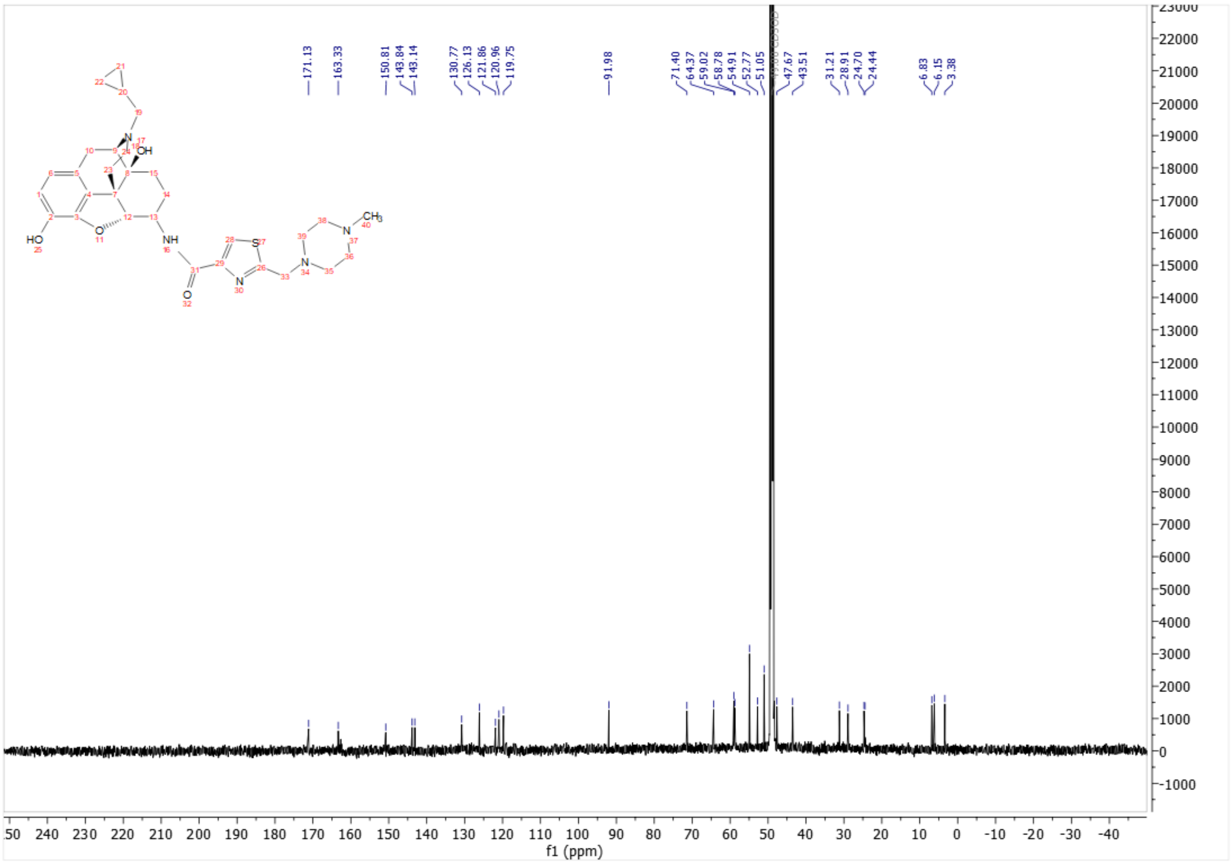

KB06 ^{1}H-NMR (MeOD, 600 MHz)

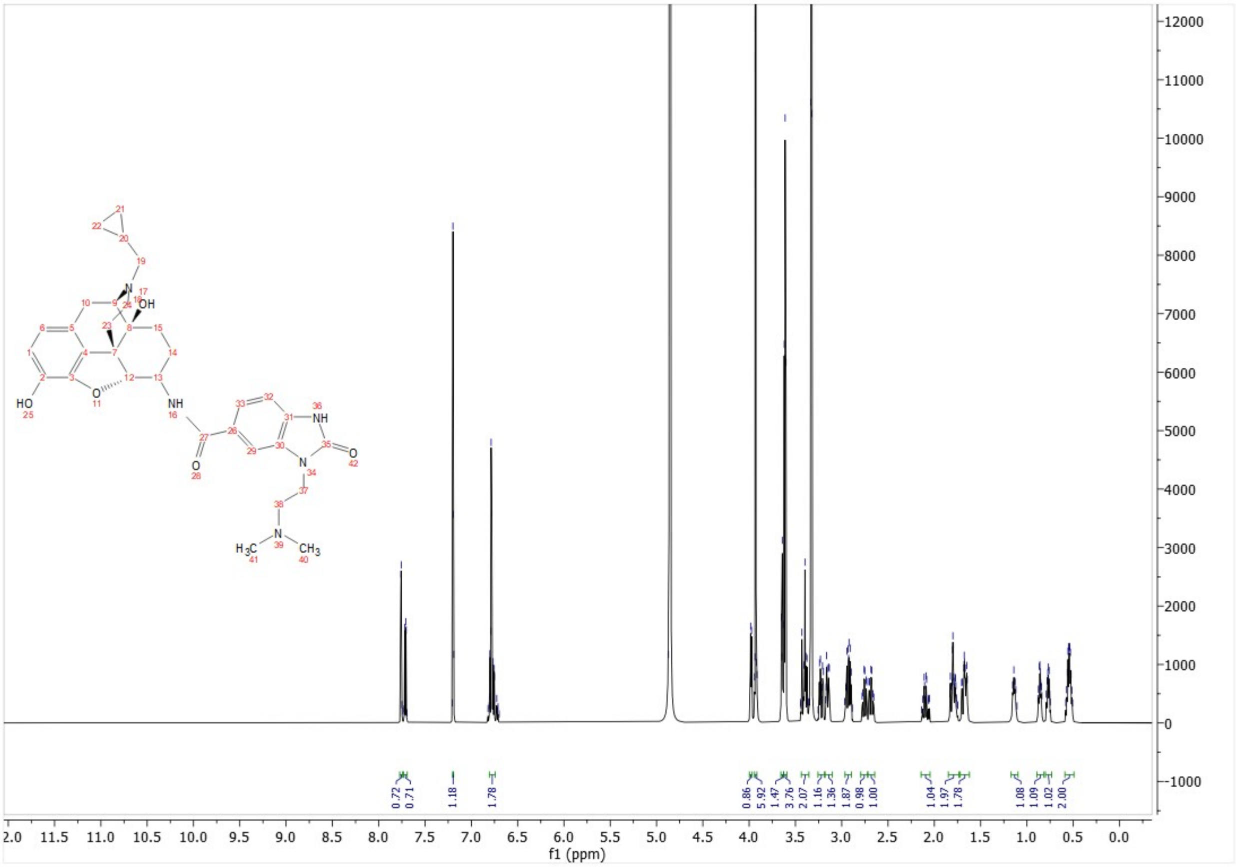

KB06 ^{13}C-NMR (MeOD, 600 MHz)

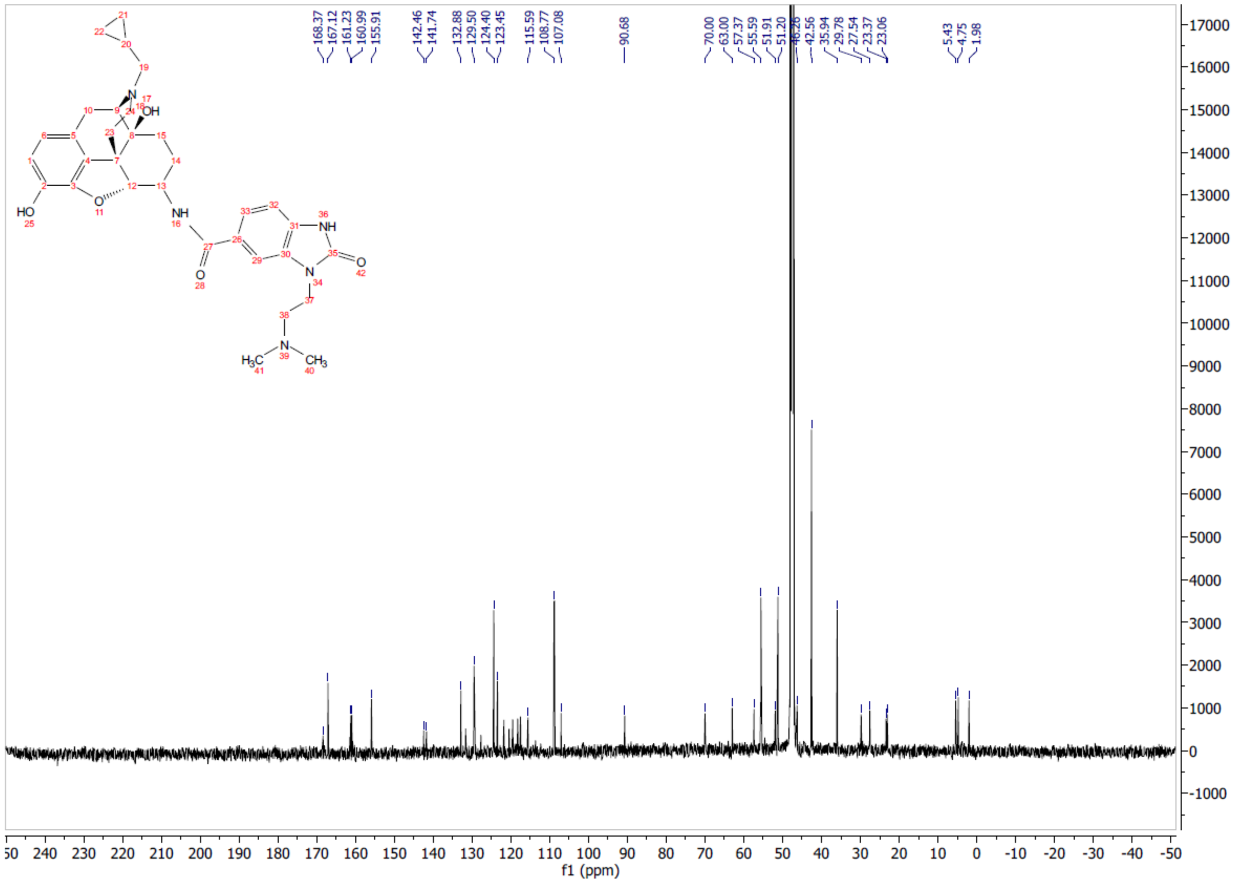

KB07 ^{1}H-NMR (MeOD, 600 MHz)

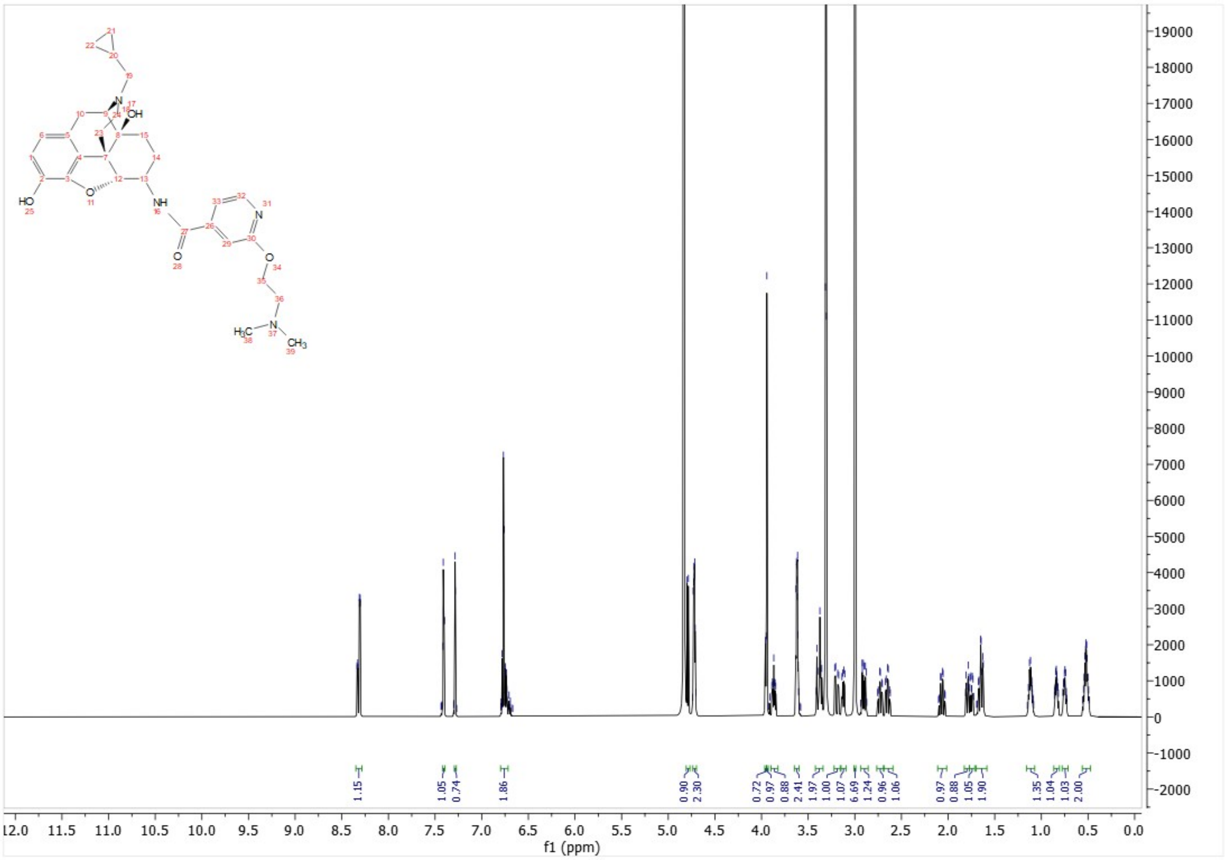

KB07 ^{13}C-NMR (MeOD, 600 MHz)

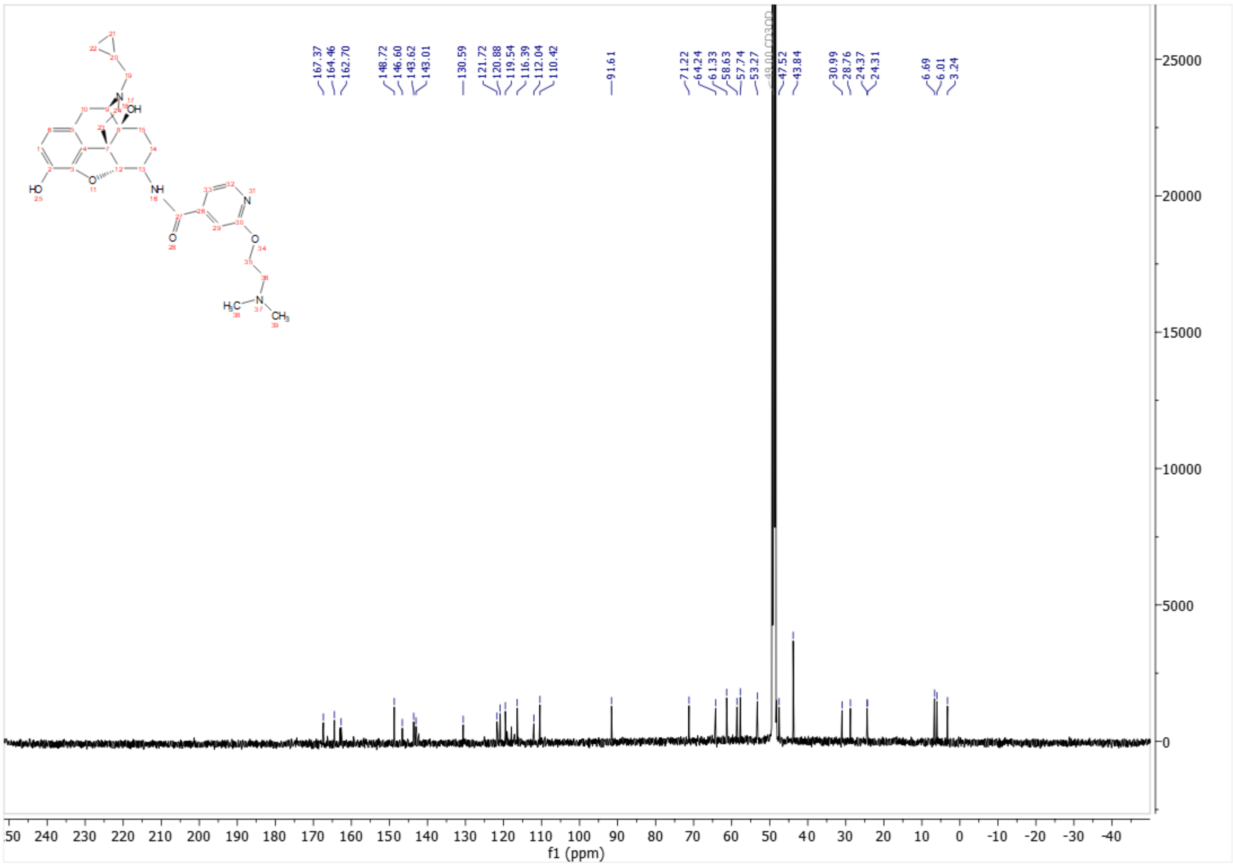

KB08 ^{1}H-NMR (MeOD, 300 MHz)

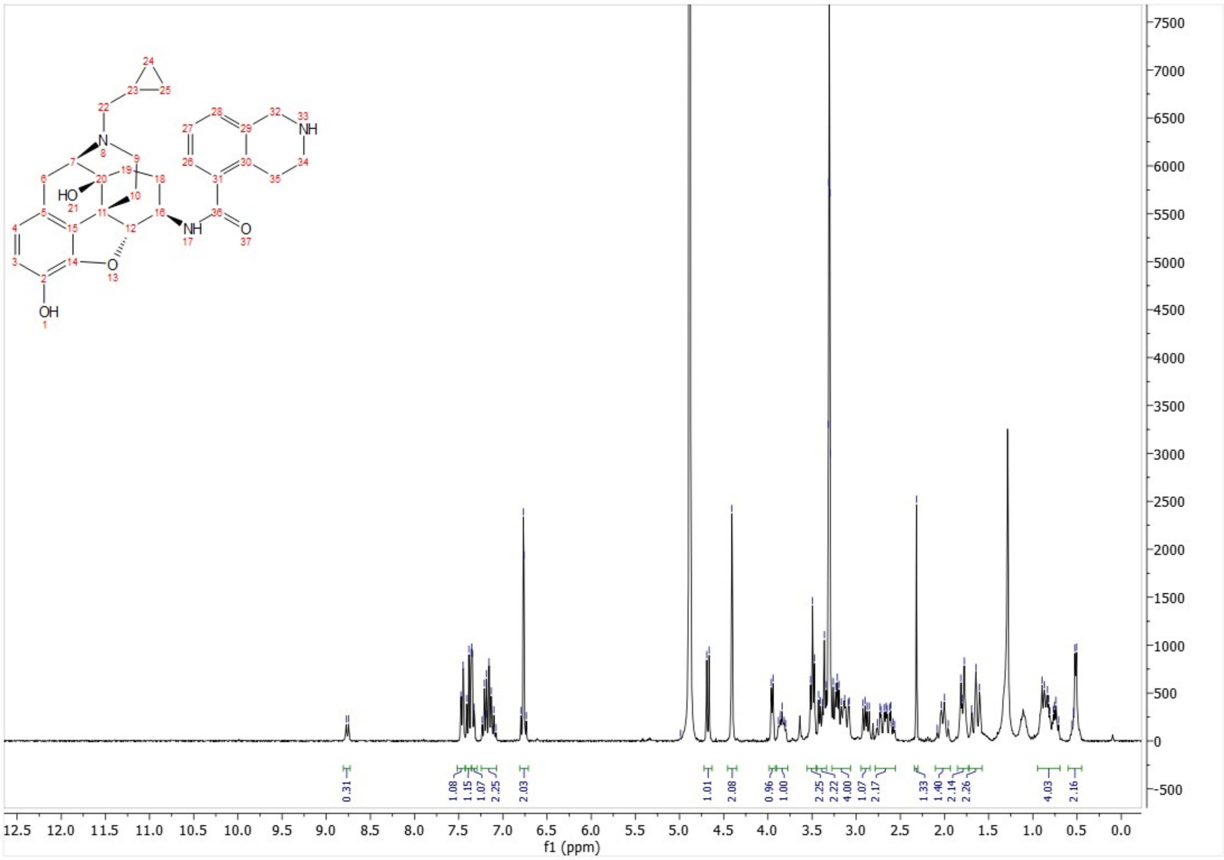

KB08 ^{13}C-NMR (MeOD, 300 MHz)

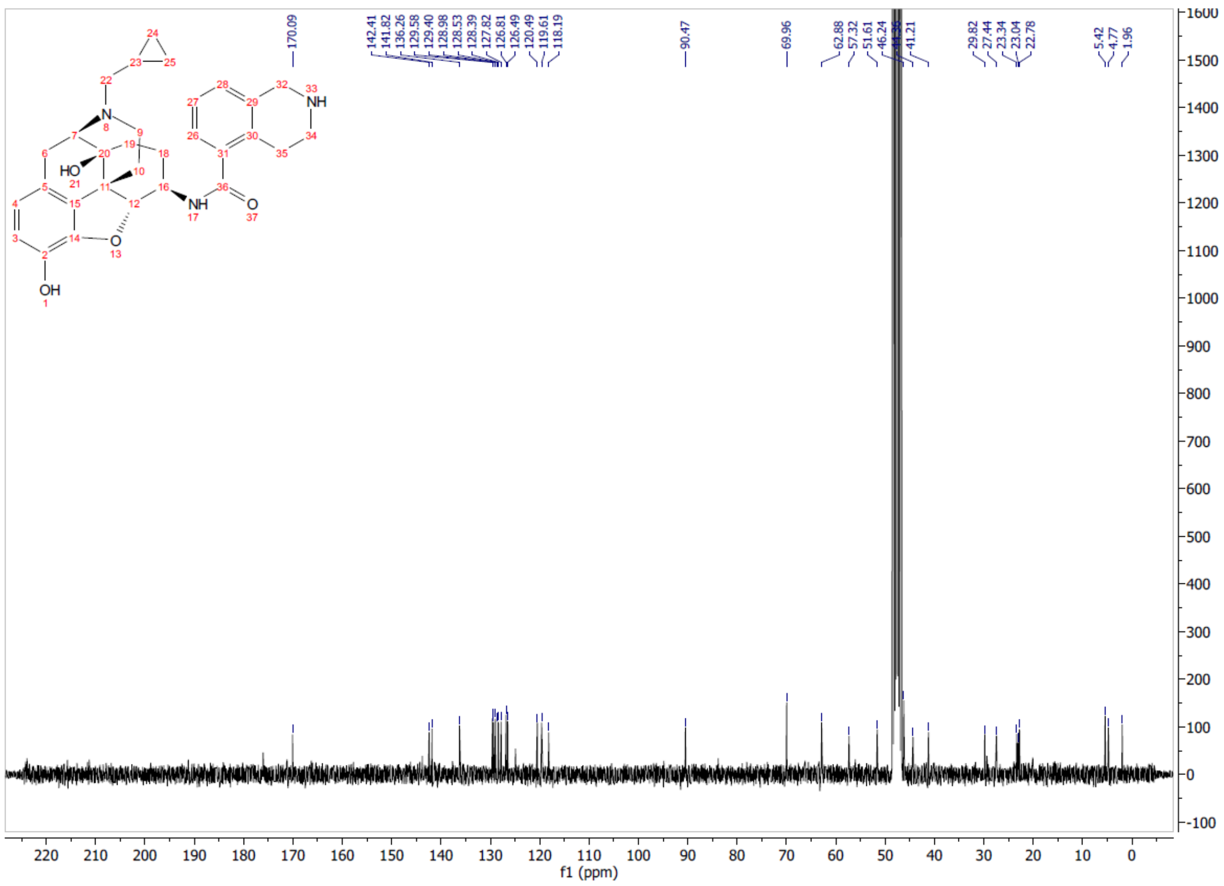

Supplement: Supplementary file 1 [file jm5c02135_si_001.pdf]
